# Global, regional, and national burden of myocarditis in children aged 0–14 years, 1990–2021: analysis for the global burden of disease study 2021

**DOI:** 10.3389/fpubh.2024.1504586

**Published:** 2024-12-20

**Authors:** Yi-Dong Zhang, Nuo Chen, Qiao-Yu Wang, Hao Li, Song-Yue Zhang, Tian-He Xia, Yue-E. He, Xing Rong, Ting-Ting Wu, Rong-Zhou Wu

**Affiliations:** Second Affiliated Hospital and Yuying Children’s Hospital of Wenzhou Medical University, Wenzhou, China

**Keywords:** children, myocarditis, global burden of disease, disability-adjusted life years, estimated annual percentage changes

## Abstract

**Objective:**

There are limited epidemiological data on myocarditis in children aged 0–14 years. This study aims to investigate the trends in incidence, mortality, disability-adjusted life years (DALYs), and corresponding estimated annual percentage change (EAPC) of myocarditis in children aged 0–14 years from 1990 to 2021.

**Methods:**

We utilized the 2021 Global Burden of Disease, Injuries, and Risk Factors Study (GBD) analytical tools to examine the incidence, mortality, and DALYs of myocarditis in children aged 0–14 years, considering factors such as age, sex, region, sociodemographic index (SDI), and data from 204 countries or regions.

**Results:**

In 2021, a total of 155.45/1000 people cases of myocarditis were reported globally in children. The cases of myocarditis in children increased from 143.80/1000 people (95% uncertainty interval [UI], 93.13–214.67) in 1990 to 155.45/1000 people (95% UI, 100.31–232.31) in 2021, increasing by 8.1% (95% UI, 6.04–9.73%). Over 30 years, the global incidence rate decreased from 8.27 (95% UI, 5.35–12.34) to 7.73 (95% UI, 4.99–11.55) per 100,000 population. The myocarditis-associated mortality rate decreased from 0.36 (95% UI, 0.25–0.51) to 0.13 (95% UI, 0.10–0.16) per 100,000 population. In 2021, the highest incidence of myocarditis in children occurred in High SDI regions. Regionally, High-income Asia Pacific had the greatest increase in incidence (EAPC, 0.25; 95% CI, 0.22–0.28). Japan had the highest incidence rate of myocarditis in children, while Haiti reported the highest myocarditis-associated mortality rate and DALYs rate. Globally, environmental/occupational risk, nonoptimal temperature, high temperature, and low temperature were key risk factors for myocarditis-associated mortality in children.

**Conclusion:**

Between 1990 and 2021, myocarditis in children saw declining mortality and DALYs but rising incidence, especially in males. Children under 1 year face higher mortality and DALY rates despite lower incidence, stressing early diagnosis. High SDI regions report higher incidence but lower mortality, while low SDI areas need standardized treatment. Japan had the highest 2021 incidence, and China had the most deaths. Underscoring the urgency for enhanced medical resources, comprehensive research into the disease’s etiology, and improved prevention strategies.

## 1 Introduction

Myocarditis is a significant global public health issue, particularly as a leading cause of sudden cardiac death in young individuals (1). Myocarditis refers to a clinical and histological manifestation of pathological immune processes within the heart, where immune responses result in structural and functional abnormalities of myocardial cells, leading to impaired cardiac contractility and ventricular remodeling (2, 3). While the clinical prognosis of myocarditis is generally favorable, it can result in severe outcomes such as cardiogenic shock, sudden cardiac death, and dilated cardiomyopathy (4). In children, the disease is often more severe than in adults (5). Current reports estimate the global incidence of myocarditis to range between 10.2 and 105.6 cases per 100,000 people, with approximately 1.8 million new cases annually (6, 7). Recent studies highlight an increase in myocarditis incidence and mortality, with 1,265.77/1,000 people cases and 324.49/1,000 people deaths in 2019, reflecting a 62.19% rise in incidence and a 65.40% increase in mortality compared to 1990 (7). From 1990 to 2019, the global burden of myocarditis has steadily grown, underscoring the need for effective prevention and treatment strategies. Despite this, there is limited epidemiological data on myocarditis specifically in children aged 0–14 years. The lack of focused data impedes a comprehensive understanding of the disease’s impact on this vulnerable population and limits evidence-based policy-making.

Epidemiological databases are crucial for assessing disease burden and informing public health interventions. Among the available resources, the Global Burden of Disease (GBD) database stands out as a robust international research initiative. It systematically integrates metrics such as incidence, mortality, and disability-adjusted life years (DALYs) to assess the impact of diseases, injuries, and risk factors across different countries and regions. GBD provides comprehensive health data, enabling policymakers and public health professionals to better understand the burden of diseases and guide resource allocation (8–10). While a few studies have reported on myocarditis, no studies have specifically focused on myocarditis in children aged 0–14 years. Therefore, this study aims to leverage the GBD database to conduct a detailed analysis of myocarditis in children, which could assist policymakers in optimizing healthcare resource allocation and reducing the risk of myocarditis in children.

## 2 Methods

### 2.1 Overview and data sources

This study used the 2021 GBD dataset, which provides comprehensive information on the global and regional burden of 369 diseases and injuries, as well as 88 risk factors across 204 countries and regions from 1990 to 2021 (10). Data on myocarditis in children aged 0–14 years were accessed through the Global Health Data Exchange (GHDx) query tool.^1^ Theglobalburdenof myocarditis in children aged 0–14 years was assessed based on incidence, mortality, DALYs, and their respective rates, stratified by age, sex, region, and Sociodemographic Index (SDI) (The regional division of SDI can be obtained from Institute for Health Metrics and Evaluation)^2^ (11). The SDI is classified according to quintiles of the geometric mean of per capita income, total fertility rate (age < 25 years), and average educational attainment (age ≥ 15 years), reflecting a country’s overall social and economic development (10). DALYs, a composite measure of disease burden, are the sum of years of life lost (YLL) and years lived with disability (YLD) (7). Additionally, In GBD 2021, risk factor exposure data were estimated using spatiotemporal Gaussian process regression or DisMod-MR 2.1 (12). The GBD 2021 risk factor analysis assessed 88 risk factors and their health outcomes using data from 54,561 sources, generating estimates for 631 risk–outcome pairs. These pairs were selected based on data-driven evidence of association and analyzed globally, regionally, and nationally by age, sex, and year. Using a comparative risk assessment framework, the study estimated relative risks (RRs), summary exposure values (SEVs), and theoretical minimum risk exposure levels (TMRELs) for each pair. Population attributable fractions (PAFs) were calculated to measure the proportional health impact of reducing risk factor exposure to TMREL. PAFs, combined with disease burden in disability-adjusted life-years (DALYs), quantified the attributable burden of each risk factor or combination (10, 13). This study, the 2021 GBD database identified 4 risk factors for myocarditis in children: environmental/occupational risks, nonoptimal temperature, high temperature, and low temperature. These risk factors were automatically matched by GBD 2021 when selecting myocarditis. The GBD database does not include participant information on race or ethnicity.

### 2.2 Statistical analysis

The primary indicators used to describe the burden of myocarditis in children were incidence, mortality, DALYs, and corresponding rates. We performed subgroup analyses stratified by age (<1 year, 2–4 years, 5–9 years, and 10–14 years), sex (men and women), SDI (high, high-middle, middle, low-middle, and low SDI categories), 21 GBD regions (South Asia and East Asia, North Africa and Middle East, Western Sub-Saharan Africa, Southeast Asia, Eastern Sub-Saharan Africa, Central Latin America, Tropical Latin America, Central Sub-Saharan Africa, Western Europe, High-income North America, Central Asia, Eastern Europe, Southern Sub-Saharan Africa, High-income Asia Pacific, Central Europe, Andean Latin America, Caribbean, Southern Latin America, Oceania, Australasia) and 204 countries/territories. Each rate was reported per 100,000 individuals along with a 95% uncertainty interval (UI), as calculated using the GBD methodology (14). Linear regression models were employed to calculate mean estimated annual percentage changes (EAPCs) (15). EAPCs were used to assess time trends in the burden of myocarditis in children (16). A negative EAPC and its 95% CI upper bound indicated a declining trend, while a positive EAPC and its 95% CI lower bound indicated an increasing trend (11). Pearson correlation analysis was used to evaluate the relationship between EAPC and incidence, mortality, DALYs, and SDI. We calculated the proportional contribution of each risk factor by myocarditis-associated mortality. All data analyses were conducted using R software (version 4.4.1), with 2-sided *p*-values, and statistical significance was set at *p* < 0.05.

## 3 Results

### 3.1 Myocarditis in children: global trend

#### 3.1.1 Incidence

**Figure 1:**
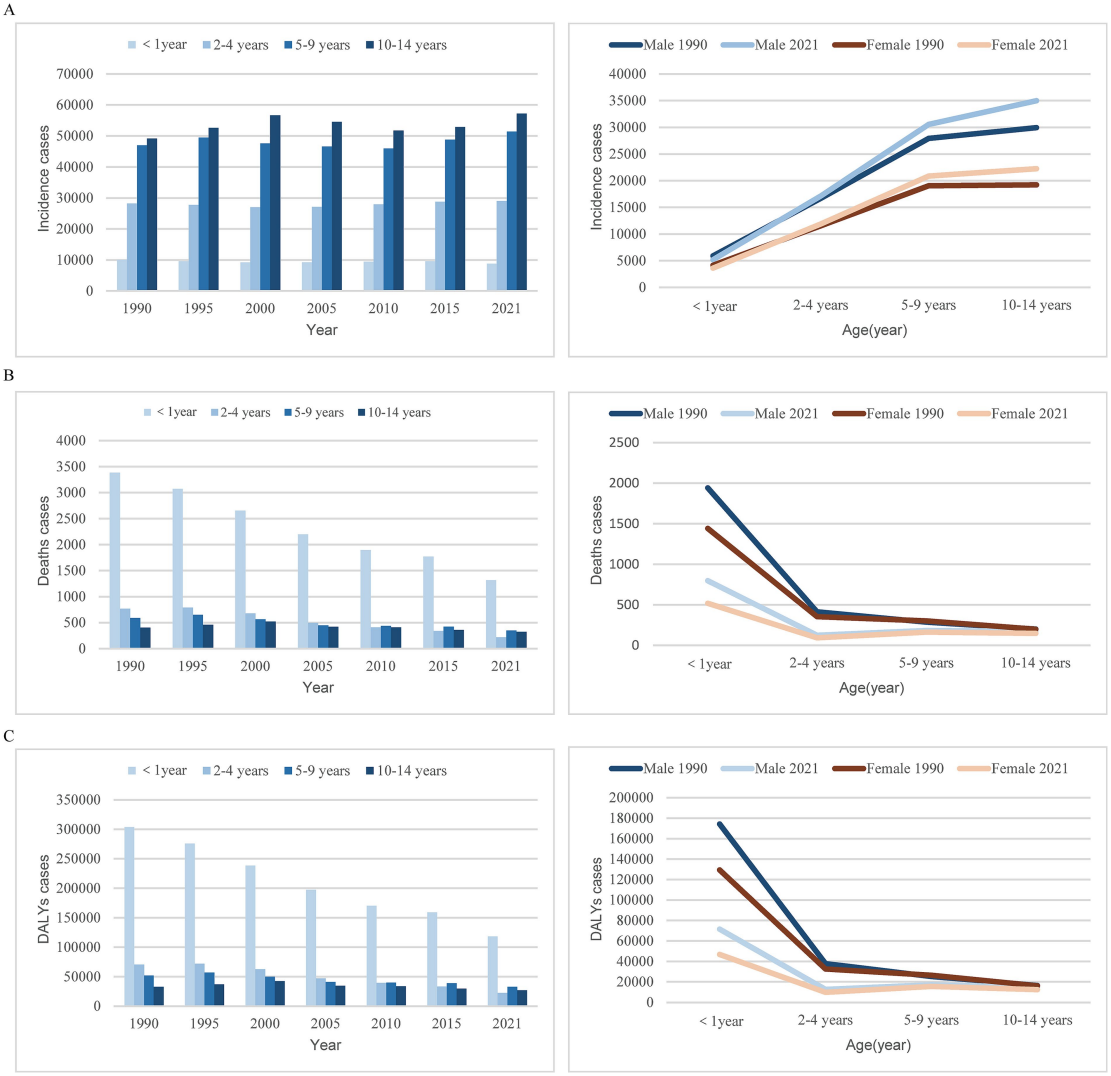
Trends in myocarditis incidence, deaths, and DALYs among children from 1990 to 2021. **(A)** Trends in incident cases. **(B)** Trends in death cases. **(C)** Trends in DALYs cases. DALYs, disability-adjusted life-years.

In 2021, there were 155.45/1,000 people (95% UI, 100.31–232.31) global cases of myocarditis in children, with males accounting for 59.94% (93.18/1,000 people, 95% UI, 60.26–138.77). From 1990 to 2021, the global incident cases of myocarditis in children increased by 8.10% (95% UI, 6.04–9.73%), although the incidence rate decreased from 8.27 (95% UI, 5.35–12.34) in 1990 to 7.73 (95% UI, 4.99–11.55) in 2021. The EAPC was −0.29 (95% CI, −0.33 – −0.26). During this period, the incident cases increased among children aged 2–14 years, with the highest increase observed in those aged 10–14 years, while decreased among children aged <1 year. Across both years, males had consistently higher incident cases than females, and across all age groups, incidence rates increased with age (Table 1 and Figure 1A).

**Table 1:** Incidence of myocarditis in children between 1990 and 2021 at the global and regional level.

	Rate per 100,000 (95% UI) Case per 1,000 (95% UI)					
	1990		2021		1990–2021	
Location	Incident cases	Incident rate	Incident cases	Incident rate	Cases change	EAPCs
Global	143.80 (93.13–214.67)	8.27 (5.35–12.34)	155.45 (100.31–232.31)	7.73 (4.99–11.55)	8.10 (6.04–9.73)	−0.29 (−0.33–−0.26)
Sex						
Female	57.82 (37.17–86.83)	6.84 (4.40–10.27)	62.27 (39.63–93.81)	6.40 (4.07–9.63)	7.70 (5.44–9.65)	−0.27 (−0.30–−0.24)
Male	85.98 (55.87–127.67)	9.62 (6.25–14.29)	93.18 (60.26–138.77)	8.98 (5.80–13.37)	8.37 (6.23–10.09)	−0.31 (−0.35–−0.27)
SDI						
High	16.22 (10.59–23.75)	8.73 (5.70–12.78)	14.10 (9.58–20.15)	8.17 (5.55–11.68)	−13.07 (−15.61–−9.80)	−0.33 (−0.43–−0.23)
High Middle SDI	24.22 (15.61–35.73)	8.85 (5.70–13.06)	18.17 (11.87–26.88)	7.87 (5.14–11.64)	−24.98 (−26.90–−23.15)	−0.56 (−0.66–−0.47)
Middle SDI	50.97 (32.86–76.29)	8.83 (5.69–13.22)	46.20 (29.56–69.47)	8.15 (5.21–12.26)	−9.35 (−11.56–−7.55)	−0.36 (−0.41–−0.30)
Low Middle SDI	35.78 (23.01–53.84)	7.58 (4.87–11.40)	43.69 (27.65–66.31)	7.53 (4.77–11.44)	22.11 (19.04–24.30)	−0.03 (−0.04–−0.03)
Low SDI	16.50 (10.69–24.53)	7.21 (4.67–10.71)	33.17 (21.27–49.63)	7.21 (4.62–10.78)	101.06 (96.06–104.52)	−0.00 (−0.00–0.00)
Regions						
Andean Latin America	0.95 (0.61–1.43)	6.41 (4.12–9.60)	1.17 (0.75–1.75)	6.45 (4.12–9.67)	22.69 (20.20–25.21)	0.04 (0.03–0.05)
Australasia	0.31 (0.20–0.46)	6.84 (4.40–10.04)	0.39 (0.25–0.57)	6.75 (4.33–9.95)	23.42 (21.97–25.28)	−0.09 (−0.11–−0.06)
Caribbean	0.76 (0.49–1.16)	6.65 (4.26–10.15)	0.77 (0.49–1.18)	6.69 (4.26–10.28)	1.33 (−0.33–2.49)	0.02 (0.02–0.03)
Central Asia	1.97 (1.28–2.97)	7.90 (5.12–11.87)	2.20 (1.42–3.34)	7.96 (5.13–12.08)	11.58 (10.24–12.65)	0.00 (−0.02–0.03)
Central Europe	2.40 (1.57–3.50)	8.13 (5.32–11.88)	1.44 (0.95–2.11)	8.16 (5.36–11.91)	−39.72 (−39.93–−39.47)	−0.08 (−0.12–−0.03)
Central Latin America	4.55 (2.89–6.85)	7.06 (4.49–10.64)	4.52 (2.81–6.86)	7.12 (4.43–10.81)	−0.58 (−3.05–1.17)	0.03 (0.02–0.03)
Central Sub-Saharan Africa	1.73 (1.13–2.53)	6.86 (4.48–9.99)	4.06 (2.63–6.01)	6.91 (4.48–10.23)	133.94 (126.39–139.76)	0.02 (0.01–0.02)
East Asia	34.04 (21.77–50.20)	10.32 (6.60–15.22)	22.28 (14.53–32.91)	8.33 (5.43–12.31)	−34.56 (−36.87–−32.05)	−0.94 (−1.11–−0.78)
Eastern Europe	4.52 (2.90–6.91)	8.79 (5.64–13.44)	3.15 (2.00–4.89)	8.90 (5.65–13.79)	−30.28 (−32.18–−28.93)	−0.05 (−0.09–−0.02)
Eastern Sub-Saharan Africa	6.54 (4.25–9.70)	7.22 (4.69–10.71)	12.93 (8.30–19.33)	7.25 (4.65–10.83)	97.67 (92.63–101.35)	0.01 (0.00–0.01)
High-income Asia Pacific	4.28 (2.80–6.32)	12.17 (7.94–17.96)	2.89 (1.92–4.24)	12.90 (8.58–18.89)	−32.45 (−33.89–−30.73)	0.25 (0.22–0.28)
High-income North America	4.24 (2.64–6.48)	6.88 (4.28–10.50)	4.09 (2.83–5.76)	6.24 (4.31–8.77)	−3.52 (−12.54–8.31)	−0.68 (−0.95–−0.41)
North Africa and Middle East	7.60 (4.79–11.63)	5.41 (3.41–8.28)	9.85 (6.14–1.53)	5.37 (3.35–8.32)	29.59 (26.17–32.12)	−0.07 (−0.08–−0.05)
Oceania	2.51 (1.66–3.69)	9.35 (6.19–13.77)	0.48 (0.32–0.70)	9.35 (6.21–13.75)	89.54 (89.08–90.16)	0.00 (−0.00–0.00)
South Asia	33.45 (21.45–50.81)	7.72 (4.95–11.72)	39.80 (24.97–60.88)	7.85 (4.93–12.01)	18.99 (15.14–21.72)	0.04 (0.04–0.05)
Southeast Asia	17.07 (11.15–25.60)	10.00 (6.53–14.99)	17.40 (11.30–26.27)	10.08 (6.55–15.21)	1.95 (0.41–3.37)	0.03 (0.03–0.03)
Southern Latin America	0.90 (0.59–1.33)	6.05 (3.94–8.89)	0.86 (0.56–1.27)	5.94 (3.89–8.77)	−4.78 (−7.36–−1.81)	−0.07 (−0.08–−0.06)
Southern Sub-Saharan Africa	1.55 (0.99–2.33)	7.49 (4.81–11.28)	1.81 (1.15–2.75)	7.53 (4.78–11.42)	16.91 (14.97–18.22)	−0.01 (−0.02–0.00)
Tropical Latin America	4.02 (2.49–6.16)	7.50 (4.64–11.49)	3.75 (2.34–5.71)	7.47 (4.67–11.38)	−6.81 (−7.67–−5.57)	−0.02 (−0.03–−0.02)
Western Europe	6.28 (4.29–8.96)	8.84 (6.03–12.62)	5.90 (4.06–8.37)	8.66 (5.96–12.29)	−6.10 (−7.38–−4.65)	−0.15 (−0.21–−0.08)
Western Sub-Saharan Africa	6.37 (4.16–9.43)	7.25 (4.74–10.73)	15.71 (10.10–23.47)	7.31 (4.70–10.93)	146.44 (139.89–151.40)	0.02 (0.02–0.02)

EAPCs, estimated annual percentage changes; SDI, Sociodemographic Index; UI, uncertainty interval. An EAPC is expressed as 95% CI.

#### 3.1.2 Mortality

Over the past 30 years, the global number of myocarditis-associated mortality in children decreased by 59.11%, from 6.34/1000 people (95% UI, 4.33–8.94) in 1990 to 2.59/1,000 people (95% UI, 2.07–3.29) in 2021. Correspondingly, the myocarditis-associated mortality rate decreased from 0.36 per 100,000 (95% UI, 0.25–0.51) in 1990 to 0.13 per 100,000 (95% UI, 0.10–0.16) in 2021, with an EAPC of −3.2 (95% CI, −3.33 – −3.07) (Table 2). Children under 1 year consistently accounted for the highest proportion of deaths, whereas children aged 10–14 years had the lowest proportion. From 1990 to 2021, the largest decline in myocarditis-associated mortality cases was seen in children under 1 year, with minimal decline observed in children aged 10–14 years. In 1990, females aged 5–9 years had a higher myocarditis-associated mortality case than males, while in other age groups, male mortality cases were higher. By 2021, males had higher mortality cases in all age groups, and across both sexes, mortality cases ranked from highest to lowest as follows: <1 year, 5–9 years, 10–14 years, 2–4 years (Table 2 and Figure 1B).

**Table 2:** Deaths of myocarditis in children between 1990 and 2021 at the global and regional level.

	Rate per 100,000 (95% UI) Case per 1,000 (95% UI)					
	1990		2021		1990–2021	
Location	Deaths cases	Deaths rate	Deaths cases	Deaths rate	Cases change	EAPCs
Global	6.34 (4.33–8.944)	0.36 (0.25–0.51)	2.59 (2.07–3.29)	0.13 (0.10–0.16)	−59.11 (−71.67–−40.24)	−3.20 (−3.33–−3.07)
Sex						
Female	2.88 (1.77–4.43)	0.34 (0.21–0.52)	1.11 (0.83–1.49)	0.11 (0.09–0.15)	−61.24 (−74.63–−39.31)	−3.23 (−3.37–−3.09)
Male	3.47 (2.05–5.39)	0.39 (0.23–0.60)	1.48 (1.14–1.99)	0.14 (0.11–0.19)	−57.35 (−70.09–−25.76)	−3.18 (−3.30–−3.05)
SDI						
High	0.38 (0.35–0.42)	0.20 (0.19–0.22)	0.16 (0.14–0.17)	0.09 (0.08–0.10)	−57.33 (−63.51–−51.75)	−2.42 (−2.90–−1.93)
High Middle SDI	1.16 (0.89–1.53)	0.42 (0.33–0.56)	0.26 (0.20–0.32)	0.11 (0.09–0.14)	−77.75 (−86.07–−68.54)	−4.12 (−4.35–−3.90)
Middle SDI	3.03 (2.03–4.15)	0.53 (0.35–0.72)	0.80 (0.62–1.00)	0.14 (0.11–0.18)	−73.65 (−83.56–−58.58)	−4.07 (−4.22–−3.91)
Low Middle SDI	1.03 (0.57–2.10)	0.22 (0.12–0.45)	0.74 (0.54–1.03)	0.13 (0.09–0.18)	−28.88 (−57.56–23.55)	−1.41 (−1.56–−1.26)
Low SDI	0.74 (0.31–1.42)	0.32 (0.13–0.62)	0.64 (0.43–1.04)	0.14 (0.09–0.23)	−13.31 (−44.44–92.53)	−2.68 (−2.81–−2.56)
Regions						
Andean Latin America	0.02 (0.007–0.03)	0.10 (0.05–0.19)	0.004 (0.003–0.006)	0.02 (0.02–0.03)	−73.76 (−87.15–−42.88)	−5.08 (−5.35–−4.81)
Australasia	0.02 (0.01–0.02)	0.33 (0.30–0.35)	0.005 (0.004–0.006)	0.08 (0.07–0.10)	−68.81 (−74.37–−62.16)	−4.26 (−4.87–−3.66)
Caribbean	0.04 (0.02–0.09)	0.32 (0.17–0.79)	0.04 (0.02–0.06)	0.33 (0.18–0.55)	2.36 (−38.97–72.38)	0.45 (0.27–0.63)
Central Asia	0.03 (0.02–0.03)	0.11 (0.09–0.13)	0.02 (0.02–0.02)	0.07 (0.06–0.08)	−28.79 (−45.71–−3.66)	−0.60 (−1.50–0.30)
Central Europe	0.09 (0.08–0.10)	0.30 (0.27–0.33)	0.01 (0.01–0.02)	0.08 (0.07–0.09)	−84.06 (−87.07–−80.59)	−4.03 (−4.26–−3.79)
Central Latin America	0.05 (0.04–0.05)	0.07 (0.06–0.08)	0.05 (0.04–0.07)	0.08 (0.06–0.11)	10.40 (−20.56–50.60)	1.02 (0.68–1.36)
Central Sub-Saharan Africa	0.08 (0.03–0.20)	0.33 (0.13–0.67)	0.06 (0.03–0.10)	0.10 (0.06–0.17)	−26.22 (−59.10–90.65)	−3.40 (−3.66–−3.15)
East Asia	3.38 (2.34–4.68)	1.02 (0.71–1.42)	0.66 (0.46–0.84)	0.25 (0.17–0.31)	−80.55 (−88.40–−70.79)	−4.28 (−4.53–−4.02)
Eastern Europe	0.04 (0.04–0.05)	0.08 (0.08–0.09)	0.01 (0.01–0.02)	0.04 (0.03–0.04)	−70.23 (−74.54–−65.16)	−2.46 (−3.43–−1.48)
Eastern Sub-Saharan Africa	0.27 (0.07–0.54)	0.30 (0.08–0.60)	0.15 (0.08–0.22)	0.08 (0.05–0.12)	−46.76 (−70.36–80.04)	−4.22 (−4.33–−4.11)
High-income Asia Pacific	0.07 (0.06–0.08)	0.19 (0.16–0.23)	0.02 (0.02–0.02)	0.10 (0.09–0.11)	−68.26 (−74.95–−60.86)	−2.61 (−3.15–−2.07)
High-income North America	0.18 (0.17–0.19)	0.29 (0.27–0.30)	0.10 (0.09–0.11)	0.15 (0.13–0.17)	−44.56 (−51.48–−36.77)	−2.08 (−2.52–−1.64)
North Africa and Middle East	0.33 (0.16–0.80)	0.24 (0.11–0.57)	0.17 (0.08–0.36)	0.09 (0.04–0.19)	−49.69 (−75.35–6.96)	−2.68 (−2.93–−2.42)
Oceania	0.008 (0.004–0.01)	0.29 (0.15–0.54)	0.01 (0.007–0.03)	0.29 (0.13–0.51)	89.35 (6.81–220.91)	0.15 (0.07–0.24)
South Asia	0.92 (0.41–1.74)	0.21 (0.09–0.40)	0.68 (0.45–0.91)	0.13 (0.09–0.18)	−26.69 (−57.03–32.78)	−1.00 (−1.19–−0.81)
Southeast Asia	0.29 (0.18–0.60)	0.17 (0.11–0.35)	0.19 (0.14–0.28)	0.11 (0.08–0.16)	−35.59 (−63.00–15.46)	−1.50 (−1.61–−1.38)
Southern Latin America	0.03 (0.03–0.04)	0.23 (0.20–0.27)	0.005 (0.004–0.006)	0.03 (0.03–0.04)	−85.61 (−88.89–−81.04)	−5.77 (−6.24–−5.30)
Southern Sub-Saharan Africa	0.03 (0.02–0.04)	0.15 (0.09–0.21)	0.02 (0.02–0.03)	0.10 (0.07–0.14)	−25.60 (−56.06–40.00)	−1.22 (−1.41–−1.02)
Tropical Latin America	0.10 (0.08–0.11)	0.18 (0.15–0.21)	0.05 (0.04–0.06)	0.09 (0.07–0.12)	−50.89 (−65.24–−33.91)	−1.75 (−2.39–−1.11)
Western Europe	0.06 (0.06–0.07)	0.09 (0.08–0.09)	0.02 (0.02–0.03)	0.03 (0.03–0.04)	−64.85 (−70.42–−58.79)	−3.13 (−4.16–−2.09)
Western Sub-Saharan Africa	0.32 (0.17–0.73)	7.25 (4.74–10.73)	0.33 (0.20–0.64)	0.15 (0.09–0.30)	1.16 (−34.01–84.59)	−2.99 (−3.22–−2.76)

EAPCs, estimated annual percentage changes; SDI, Sociodemographic Index; UI, uncertainty interval. An EAPC is expressed as 95% CI.

#### 3.1.3 DALYs

The global number of myocarditis-associated DALYs decreased by 58.23%, from 566.23/1000 people (95% UI, 388.29–791.17) in 1990 to 235.99/1000 people (95% UI, 192.40–299.99) in 2021. The myocarditis-associated DALYs rate dropped from 32.56 (95% UI, 22.33–45.49) in 1990 to 11.73 (95% UI, 9.56–14.91) in 2021, with an EAPC of −3.14 (95% CI, −3.26 – −3.02) (Table 3). The sharpest decline in the number of myocarditis-associated DALYs occurred in children aged 2–4 years, while the smallest decline was in 10–14 ages. In 1990, females aged 5–9 years had higher myocarditis-associated DALYs than males, but in all other age groups, males had higher DALYs. By 2021, males had higher DALYs across all age groups, with the order from highest to lowest being <1 year, 5–9 years, 10–14 years, and 2–4 years for both genders (Table 3 and Figure 1C).

**Table 3:** DALYs of myocarditis in children between 1990 and 2021 at the global and regional level.

	Rate per 100,000 (95% UI) Case per 1,000 (95% UI)					
	1990		2021		1990–2021	
Location	DALYs cases	DALYs rate	DALYs cases	DALYs rate	Cases change	EAPCs
Global	566.23 (388.29–791.17)	32.56 (22.33–45.49)	235.99 (192.40–299.99)	11.73 (9.56–14.91)	−58.32 (−71.03–−39.29)	−3.14 (−3.26–−3.02)
Sex						
Female	256.71 (158.57–394.53)	30.36 (18.75–46.65)	101.93 (77.35–134.76)	10.47 (7.94–13.84)	−60.29 (−73.98–−38.46)	−3.16 (−3.29–−3.02)
Male	309.53 (184.19–479.85)	34.64 (20.62–53.71)	134.07 (104.68–179.16)	12.91 (10.08–17.26)	−56.69 (−69.22–−25.31)	−3.12 (−3.24–−3.01)
SDI						
High	34.98 (32.25–38.67)	18.83 (17.36–20.81)	16.98 (15.18–19.00)	9.84 (8.80–11.01)	−51.46 (−58.41–−44.51)	−2.01 (−2.49–−1.54)
High Middle SDI	103.03 (79.68–136.11)	37.66 (29.12–49.74)	23.62 (18.62–28.87)	10.23 (8.07–12.51)	−77.07 (−85.47–−68.09)	−4.03 (−4.24–−3.81)
Middle SDI	268.57 (180.18–365.76)	46.53 (31.22–63.37)	71.55 (55.61–88.67)	12.62 (9.81–15.64)	−73.36 (−83.26–−58.02)	−4.03 (−4.24–−3.81)
Low Middle SDI	93.28 (52.33–187.12)	19.76 (11.08–39.63)	66.30 (49.69–92.10)	11.43 (8.57–15.88)	−28.93 (−57.28–21.84)	−1.42 (−1.56–−1.27)
Low SDI	66.01 (27.93–126.48)	28.84 (12.20–55.25)	57.31 (39.22–92.42)	12.45 (8.52–20.08)	−13.18 (−44.14–88.69)	−2.68 (−2.80–−2.56)
Regions						
Andean Latin America	1.38 (0.71–2.51)	9.28 (4.79–16.88)	0.38 (0.27–0.51)	2.08 (1.50–2.83)	−72.67 (−86.34–−42.02)	−4.96 (−5.23–−4.70)
Australasia	1.40 (1.28–1.51)	30.46 (27.96–33.04)	0.53 (0.44–0.62)	9.17 (7.68–10.88)	−62.36 (−68.69–−55.22)	−3.77 (−4.38–−3.16)
Caribbean	3.29 (1.76–8.02)	28.82 (15.41–70.31)	3.37 (1.87–5.58)	29.27 (16.22–48.54)	2.38 (−38.88–69.94)	0.45 (0.27–0.63)
Central Asia	2.41 (2.01–2.87)	9.63 (8.05–11.50)	1.73 (1.43–2.09)	6.25 (5.18–7.56)	−28.14 (−44.76–−4.23)	−0.57 (−1.44–0.30)
Central Europe	7.96 (7.14–8.74)	26.99 (24.21–29.63)	1.41 (1.22–1.65)	7.98 (6.87–9.31)	−82.25 (−85.32–−78.49)	−3.72 (−3.92–−3.52)
Central Latin America	4.16 (3.73–4.74)	6.47 (5.80–7.36)	4.59 (3.58–5.99)	7.23 (5.64–9.44)	10.19 (−19.69–48.93)	0.99 (0.66–1.32)
Central Sub-Saharan Africa	7.36 (2.82–14.99)	29.11 (11.16–59.26)	5.40 (2.98–8.78)	9.21 (5.08–14.97)	−26.64 (−59.21–88.12)	−3.43 (−3.68–−3.17)
East Asia	297.80 (205.94–413.09)	90.29 (62.44–125.24)	58.48 (41.84–74.27)	21.88 (15.65–27.78)	−80.36 (−88.08–−70.70)	−4.24 (−4.48–−4.00)
Eastern Europe	4.12 (3.85–4.49)	8.00 (7.48–8.72)	1.27 (1.08–1.48)	3.59 (3.05–4.18)	−69.07 (−72.95–−64.64)	−2.32 (−3.21–−1.43)
Eastern Sub-Saharan Africa	24.49 (6.87–48.38)	27.04 (7.58–53.42)	13.07 (7.56–19.77)	7.32 (4.23–11.08)	−46.64 (−70.08–71.58)	−4.22 (−4.33–−4.12)
High-income Asia Pacific	6.13 (5.19–7.37)	17.42 (14.74–20.94)	2.35 (2.04–2.68)	10.48 (9.10–11.97)	−61.68 (−69.85–−52.05)	−2.05 (−2.56–−1.54)
High-income North America	16.76 (15.66–17.87)	27.17 (25.38–28.97)	10.28 (9.17–11.59)	15.67 (13.97–17.66)	−38.65 (−46.19–−30.91)	−1.76 (−2.18–−1.33)
North Africa and Middle East	29.64 (14.38–70.72)	21.10 (10.24–50.34)	15.10 (7.77–31.78)	8.23 (4.24–17.33)	−49.08 (−74.16–5.74)	−2.64 (−2.89–−2.39)
Oceania	0.69 (0.35–1.28)	25.87 (13.16–47.64)	1.31 (0.61–2.29)	25.73 (12.06–45.08)	88.58 (6.11–220.37)	0.14 (0.06–0.22)
South Asia	83.58 (38.05–154.73)	19.29 (8.78–35.71)	61.15 (41.26–81.77)	12.06 (8.14–16.13)	−26.83 (−56.78–29.84)	−1.02 (−1.20–−0.83)
Southeast Asia	25.98 (16.66–53.16)	15.22 (9.75–31.13)	16.80 (12.60–24.43)	9.73 (7.30–14.15)	−35.34 (−62.83–13.45)	−1.49 (−1.60–−1.38)
Southern Latin America	3.10 (2.68–3.59)	20.74 (17.97–24.06)	0.47 (0.38–0.58)	3.25 (2.63–3.97)	−84.78 (−88.05–−80.22)	−5.63 (−6.07–−5.18)
Southern Sub-Saharan Africa	2.78 (1.67–3.89)	13.45 (8.06–18.82)	2.06 (1.47–3.04)	8.54 (6.11–12.64)	−26.10 (−55.80–38.65)	−1.21 (−1.41–−1.00)
Tropical Latin America	8.64 (7.28–10.32)	16.11 (13.58–19.25)	4.35 (3.44–5.32)	8.66 (6.86–10.60)	−49.68 (−64.00–−33.05)	−1.70 (−2.32–−1.07)
Western Europe	5.66 (5.23–6.13)	7.97 (7.36–8.63)	2.43 (2.11–2.81)	3.57 (3.10–4.12)	−57.01 (−63.71–−49.46)	−2.45 (−3.44–−1.46)
Western Sub-Saharan Africa	28.91 (15.63–65.30)	32.90 (17.79–74.30)	29.48 (18.20–56.71)	13.73 (8.47–26.41)	1.96 (−32.58–83.43)	−2.96 (−3.19–−2.73)

DALYs, disability-adjusted life-years; EAPCs, estimated annual percentage changes; SDI, Sociodemographic Index; UI, uncertainty interval. An EAPC is expressed as 95% CI.

### 3.2 Myocarditis in children: trends by SDI

#### 3.2.1 Incidence

**Figure 2:**
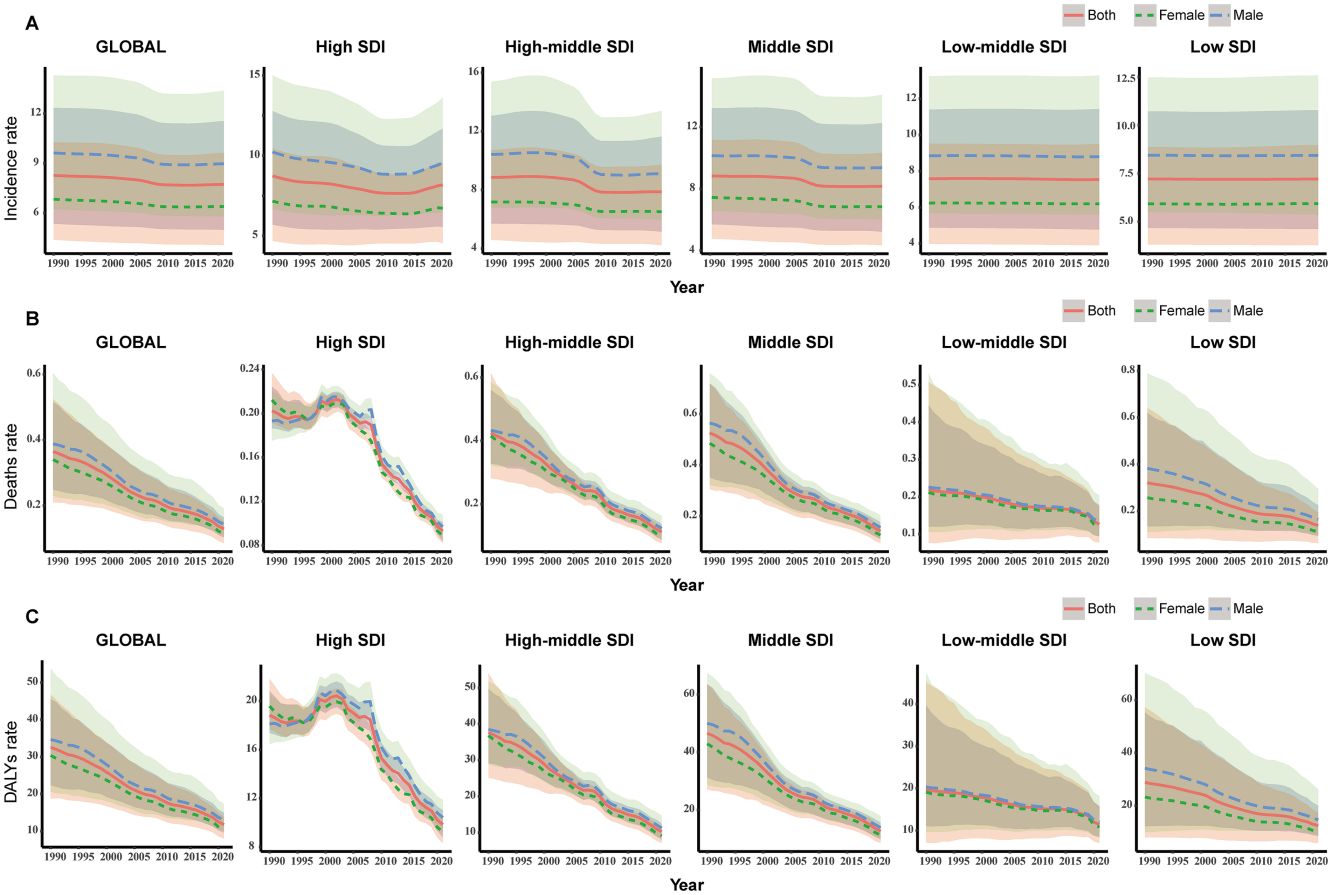
Change trends of incidence, deaths, and DALYs rate of myocarditis in children among different SDI Quintiles. **(A)** Change in incident rate. **(B)** Change in death rate. **(C)** Change in DALYs rate. DALYs, disability-adjusted life-years.

In 2021, the middle SDI region had the highest cases of myocarditis in children globally (46.20/1,000 people; 95% UI, 29.56–69.47). From 1990 to 2021, the incidence cases in the low SDI region increased by 101.06% (95% UI, 96.06–104.52%), while the cases of myocarditis in children in the middle-high SDI region decreased by 24.98% (95% UI, 23.15–26.90%). The greatest decline in incidence rate was observed in the middle-high SDI region (EAPC, −0.56; 95% CI: −0.66 – −0.47) (Table 1 and Figure 2A).

#### 3.2.2 Mortality

In 2021, the middle SDI region had the highest number of myocarditis-associated deaths (0.80/1,000 people; 95% UI, 0.62–1.00) in children, while the high SDI region had the lowest cases (0.16/1,000 people; 95% UI, 0.14–0.17). From 1990 to 2021, the myocarditis-associated mortality rate decreased in all five SDI regions, with the sharpest decline in the middle-high SDI region (77.75%; 95% UI, 68.54–86.07%). The low-middle SDI region showed the highest EAPC in myocarditis-associated mortality rate (−1.41; 95% CI, −1.56 – −1.26) (Table 2 and Figure 2B).

#### 3.2.3 DALYs

In 2021, the middle SDI region had the highest number of myocarditis-associated DALYs (71.55; 95% UI, 55.61–88.67), while the middle-high SDI region had the most significant decrease (77.07%) in the number of myocarditis-associated DALYs. The low-middle SDI region had the highest EAPC in myocarditis-associated DALYs rate (EAPC: -1.42; 95% CI, −1.56 – −1.27) (Table 3 and Figure 2C).

### 3.3 Myocarditis in children: geographic regional trends

#### 3.3.1 Incidence

**Figure 3:**
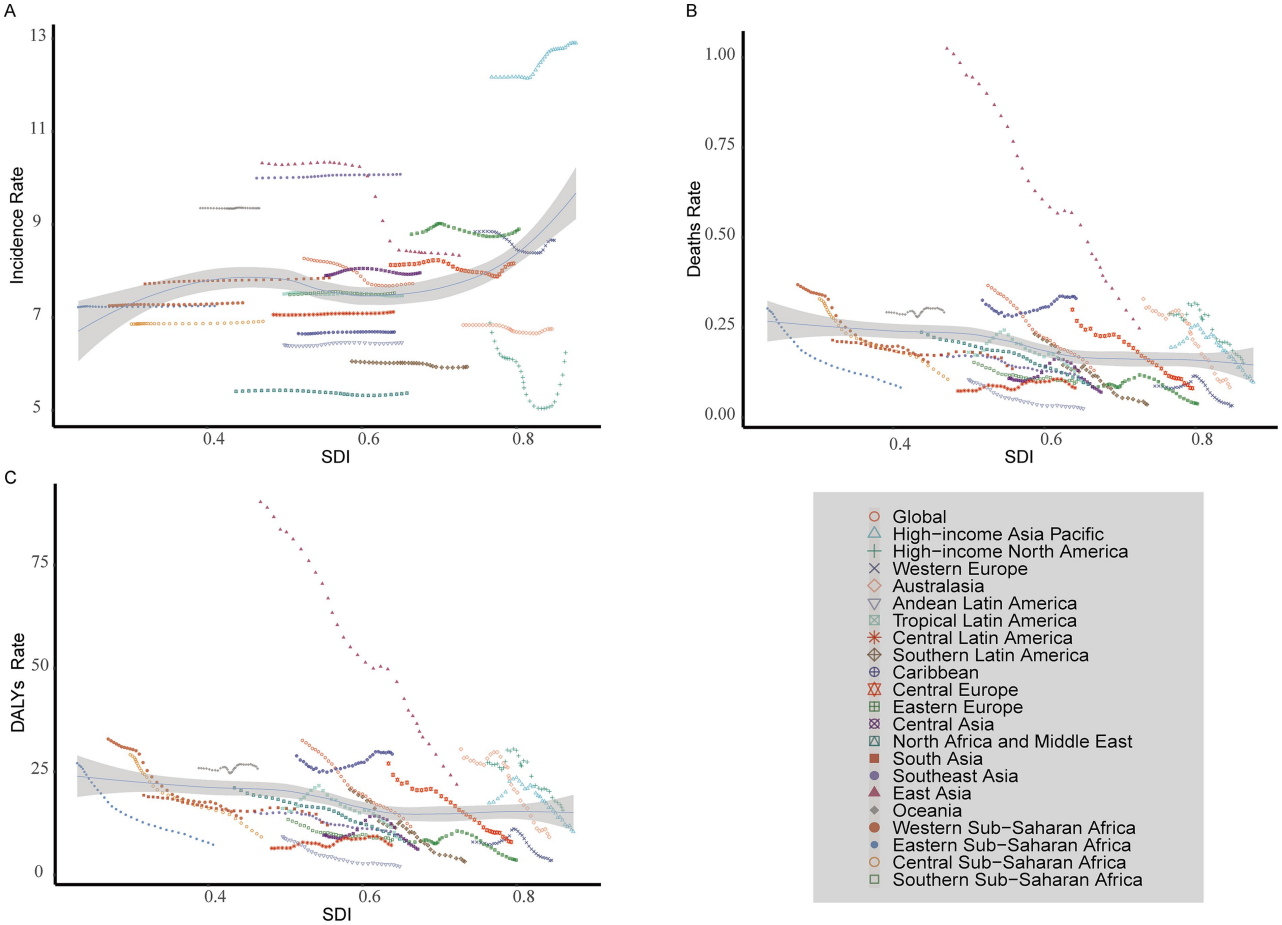
Incidence, death, and DALYs rates for myocarditis in children from 1990 to 2021. **(A)** Incident rate. **(B)** Death rate. **(C)** DALYs rate. DALYs, disability-adjusted life-years.

Among the 21 geographic regions, East Asia had the highest cases of myocarditis in children in 2021 (22.28/1,000 people; 95% UI, 14.53–32.91), whereas Australasia had the fewest (0.39/1,000 people; 95% UI, 0.25–0.57). The region with the highest incidence rate was High-income Asia Pacific (12.90; 95% UI, 8.58–18.89), and North Africa and the Middle East had the lowest rate (5.37; 95% UI, 3.35–8.32). From 1990 to 2021, the cases of myocarditis in children increased in regions such as Western Sub-Saharan Africa, South Asia, and Oceania, with the largest increase in Western Sub-Saharan Africa, where cases increased by 146.44%. Central Europe had the most significant decrease, decreased by 39.72%. High-income Asia Pacific had the greatest increase in incidence rate (EAPC, 0.25; 95% CI, 0.22–0.28), while East Asia experienced the largest decline (EAPC, −0.94; 95% CI, −1.11 to −0.78) (Table 1 and Figure 3A).

#### 3.3.2 Mortality

In 2021, South Asia had the highest cases of myocarditis-associated deaths in children (0.68/1,000 people; 95% UI, 0.45–0.91), while the Andean Latin America region had the fewest (4.04; 95% UI, 2.83–5.59). The Caribbean had the highest myocarditis-associated mortality rate (0.33; 95% UI, 0.18–0.55), whereas Andean Latin America had the lowest (0.02; 95% UI, 0.02–0.03). From 1990 to 2021, the cases of myocarditis-associated deaths in children increased in regions such as Central Latin America, Oceania, and Western Sub-Saharan Africa, while decreasing in all other regions. Specifically, Oceania had the most significant increase (89.35%). In contrast, Southern Latin America had the largest decrease (85.61%). Myocarditis-associated mortality rates in children showed an upward trend in Central Latin America, the Caribbean, and Oceania while declining in other regions. The highest increase was observed in Central Latin America (EAPC, 1.02; 95% CI, 0.68–1.36), whereas the largest decline occurred in Southern Latin America (EAPC, −5.77; 95% CI, −6.24 to −5.30) (Table 2 and Figure 3B).

#### 3.3.3 DALYs

In 2021, South Asia had the highest number of myocarditis-associated DALYs in children (61.15/1,000 people; 95% UI, 41.26–81.77), while Andean Latin America had the fewest number (0.38/1,000 people; 95% UI, 0.27–0.51). The Caribbean had the highest myocarditis-associated DALYs rate (29.27; 95% UI, 16.22–48.54), while Andean Latin America had the lowest (2.08; 95% UI, 1.50–2.83). From 1990 to 2021, the number of myocarditis-associated DALYs increased in the Caribbean, Central Latin America, Oceania, and Western Sub-Saharan Africa, with the largest increase in Oceania (88.58%) and the greatest decrease in Southern Latin America (84.78%). Central Latin America showed the highest increase in myocarditis-associated DALY rate (EAPC, 0.99; 95% CI, 0.66–1.32), whereas Southern Latin America exhibited the largest decrease (EAPC: -5.63; 95% CI, −6.07 to −5.18) (Table 3 and Figure 3C).

### 3.4 Myocarditis in children: trends by country

#### 3.4.1 Incidence

**Figure 4:**
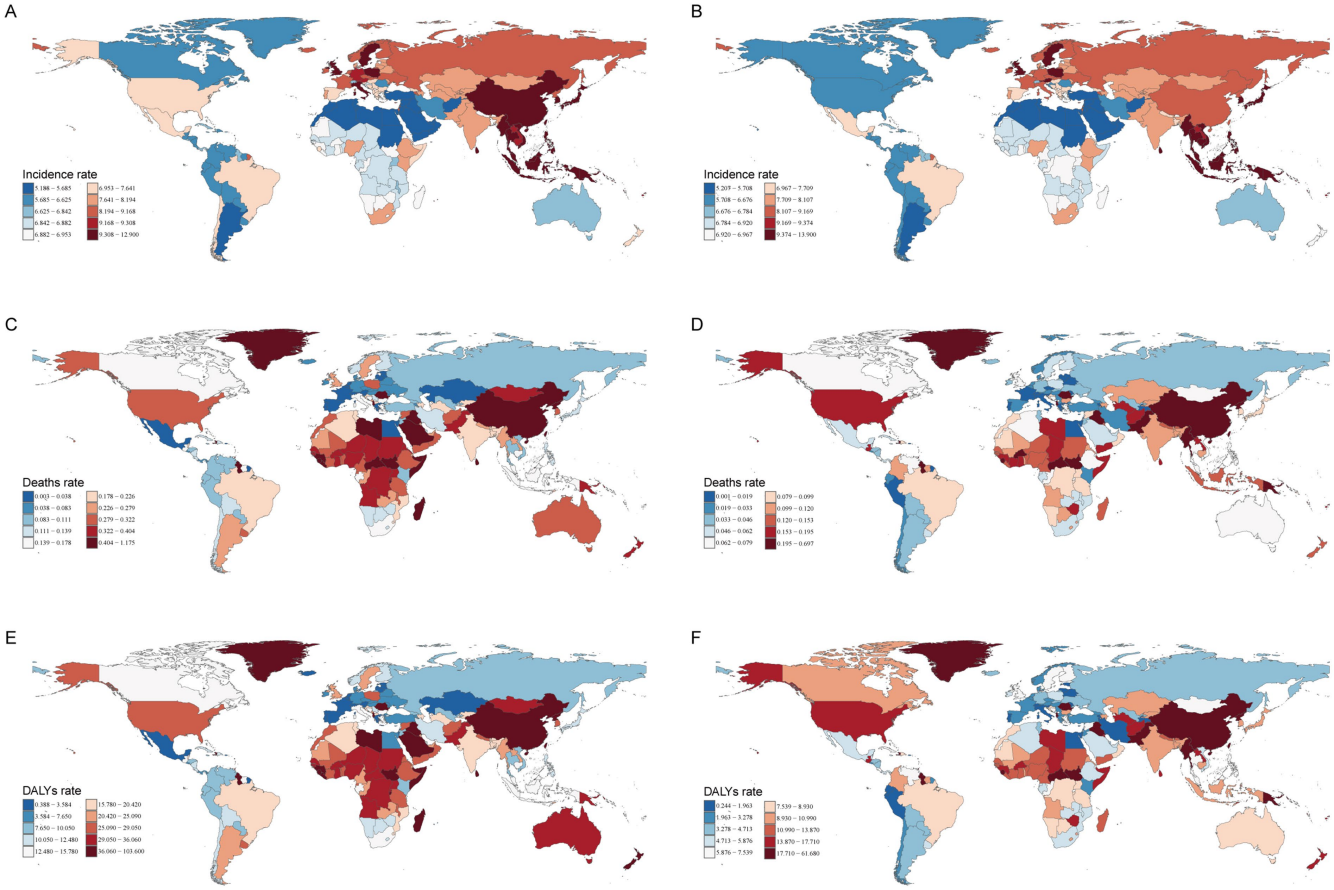
The incidence, deaths, and DALYs rates of myocarditis in children in 204 countries and territories in 1990 and 2021. **(A)** Incident rate in 1990. **(B)** Incident rate in 2021. **(C)** Death rate in 1990. **(D)** Death rate in 2021. **(E)** DALYs rate in 1990. **(F)** DALYs rate in 2021. DALYs, disability-adjusted life-years.

**Figure 5:**
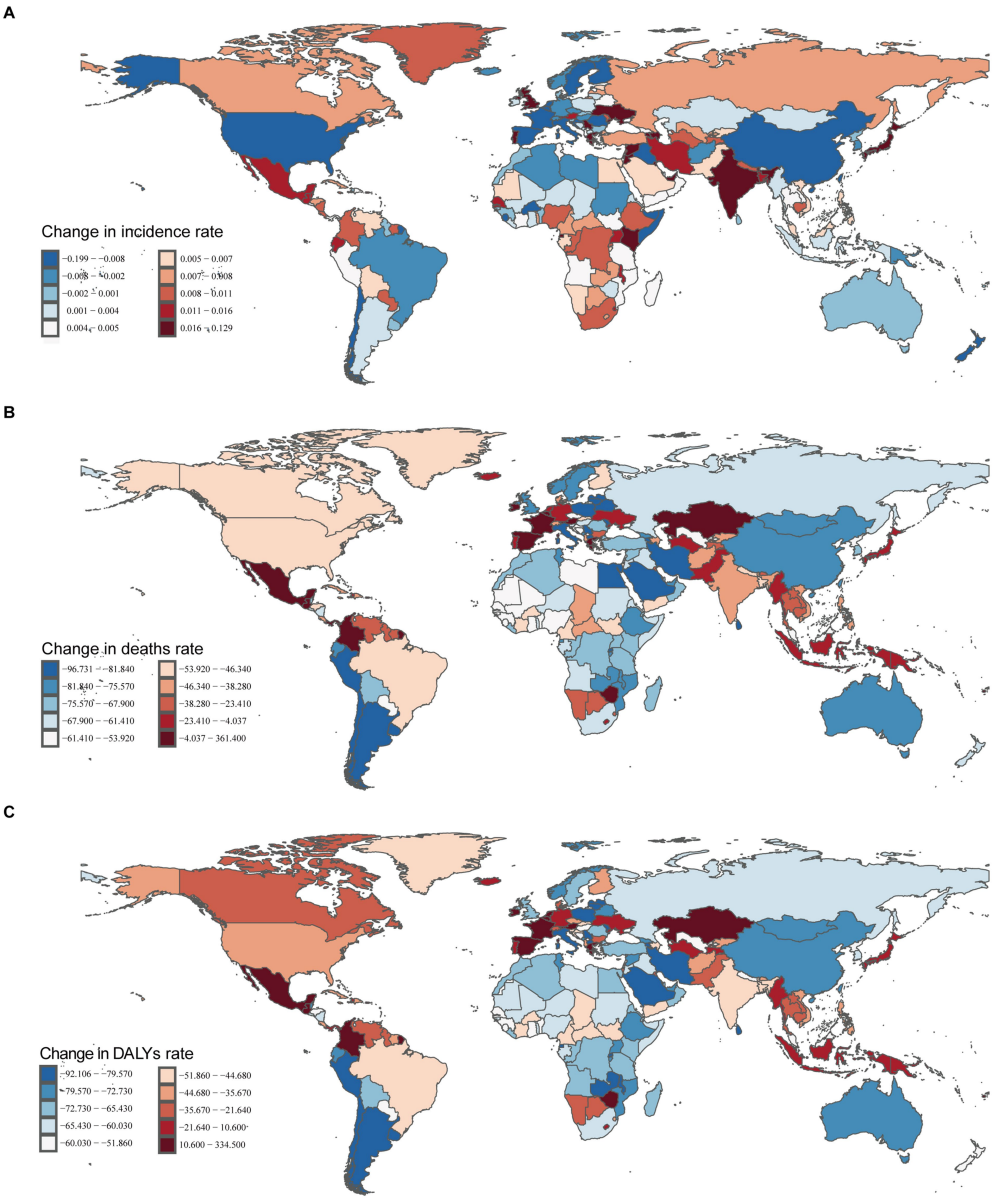
Incident, death, and DALYs rates of myocarditis in children in 204 countries and territories. **(A)** Incident rate. **(B)** Death rate. **(C)** DALYs rate.

**Figure 6:**
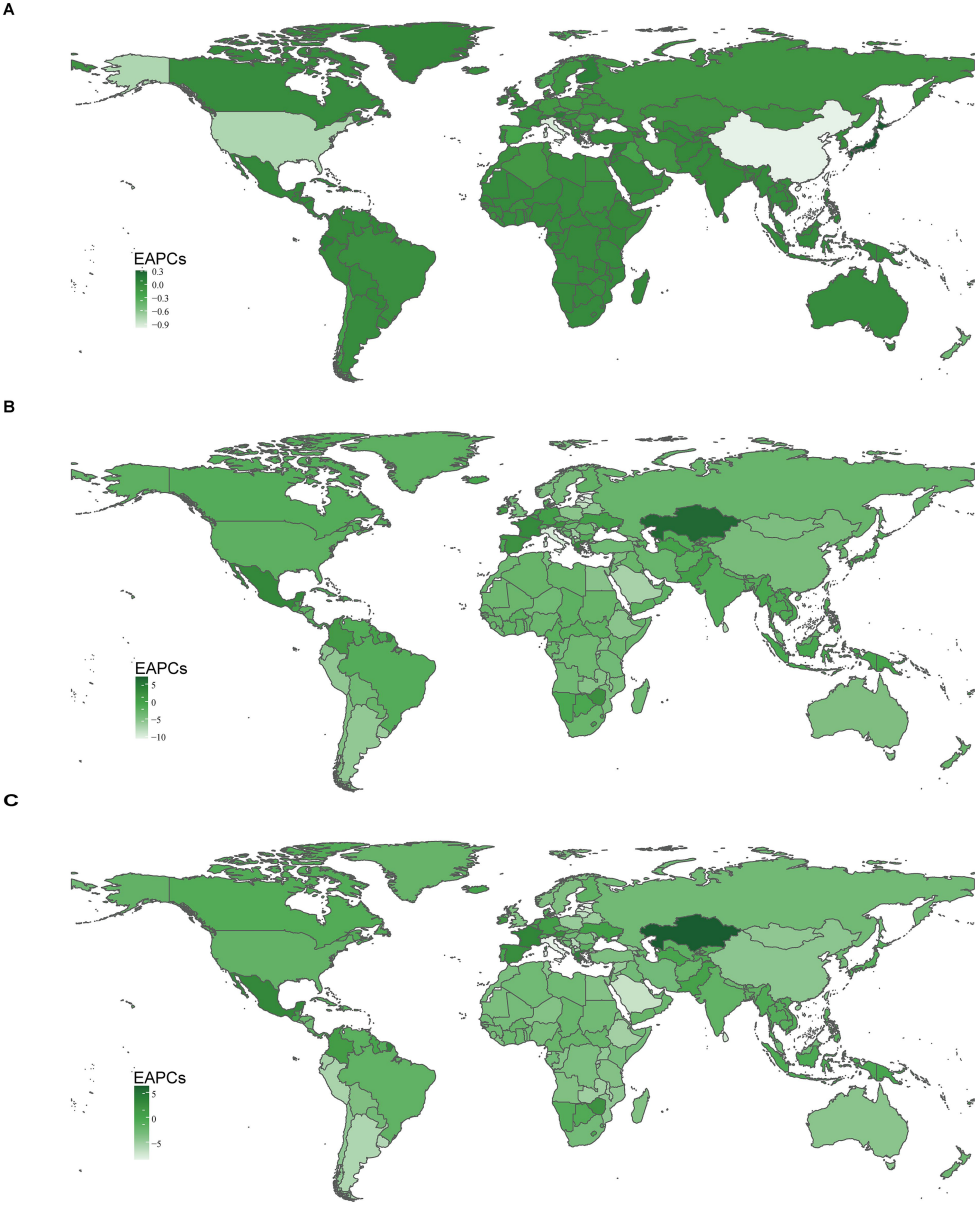
The national burden of myocarditis in children in 204 countries and territories. **(A)** EAPCs for incidence rate. **(B)** EAPCs for deaths rate. **(C)** EAPCs for DALYs rate. DALYs, disability-adjusted life-years; EAPCs, estimated annual percentage changes.

In 2021, among 204 countries, India had the highest cases of myocarditis in children 29.22/1,000 people (95% UI, 18.28–44.78), while Tokelau (0.04; 95% UI, 0.02–0.06) and Niue (0.04; 95% UI, 0.02–0.05) reported the fewest cases. Japan had the highest incidence rate 13.90 (95% UI, 9.25–20.41), whereas Qatar had the lowest 5.21 (95% UI, 3.26–8.09). Globally, the incidence rate of myocarditis in children was 7.73 (95% UI, 4.99–11.55) in 2021. Among the countries, 81 had a higher incidence rate than the global average, while 123 had a lower incidence rate. From 1990 to 2021, Qatar experienced the most significant increase in cases of myocarditis in children, increasing by 296.38%. In contrast, Albania saw the largest decline, decreased by 60.09% (Supplementary Table 1 and Figures 4A,B). The most notable upward trend in incidence rate was observed in Japan (EAPC, 0.31; 95% CI, 0.28–0.35), while China had the steepest decrease (EAPC, −0.98; 95% CI, −1.16– −0.80) (Supplementary Table 1 and Figures 5A, 6A).

#### 3.4.2 Mortality

In 2021, China had the highest cases of myocarditis-associated deaths 0.64/1000 people (95% UI, 0.45–0.82), while the Cook Islands had the fewest. Haiti had the highest myocarditis-associated mortality rate 0.70 (95% UI, 0.31–1.26), and Estonia had the lowest myocarditis-associated mortality rate (Supplementary Table 2 and Figures 4C,D). The global myocarditis-associated mortality rate in 2021 was 0.13 (95% UI, 0.10–0.16), with 57 countries having a higher myocarditis-associated mortality rate and 147 countries below the global average (Supplementary Table 2). From 1990 to 2021, Estonia had the most significant decrease in myocarditis-associated mortality rate (EAPC, −10.62; 95% CI, −11.83– −9.40), whereas Mauritius had the largest increase (EAPC, 7.11; 95% CI, 3.92–10.39) (Supplementary Table 2 and Figures 5B, 6B).

#### 3.4.3 DALYs

In 2021, China had the highest number of myocarditis-associated DALYs in children 57.04/1,000 people (95% UI, 40.82–72.39), while the Cook Islands had the lowest 0.01 (95% UI, 0.01–0.02). Haiti had the highest myocarditis-associated DALYs rate 61.68 (95% UI, 27.51–111.64), whereas Latvia had the lowest 0.24 (95% UI, 0.18–0.34) (Supplementary Table 3 and Figures 4E,F). In 2021, the global myocarditis-associated DALY rate in children was 11.73 (95% UI, 9.56–14.91), 57 countries were higher than the global average, while 147 countries were below the global average (Supplementary Table 3). From 1990 to 2021, Italy experienced the greatest decrease in myocarditis-associated DALY rate (EAPC, −8.51; 95% CI, −9.60– −7.40), whereas Kazakhstan showed the most significant increase (EAPC, 6.30; 95% CI, 4.68–7.94) (Supplementary Table 3 and Figures 5C, 6C).

### 3.5 Risk factors for myocarditis in children

**Figure 7:**
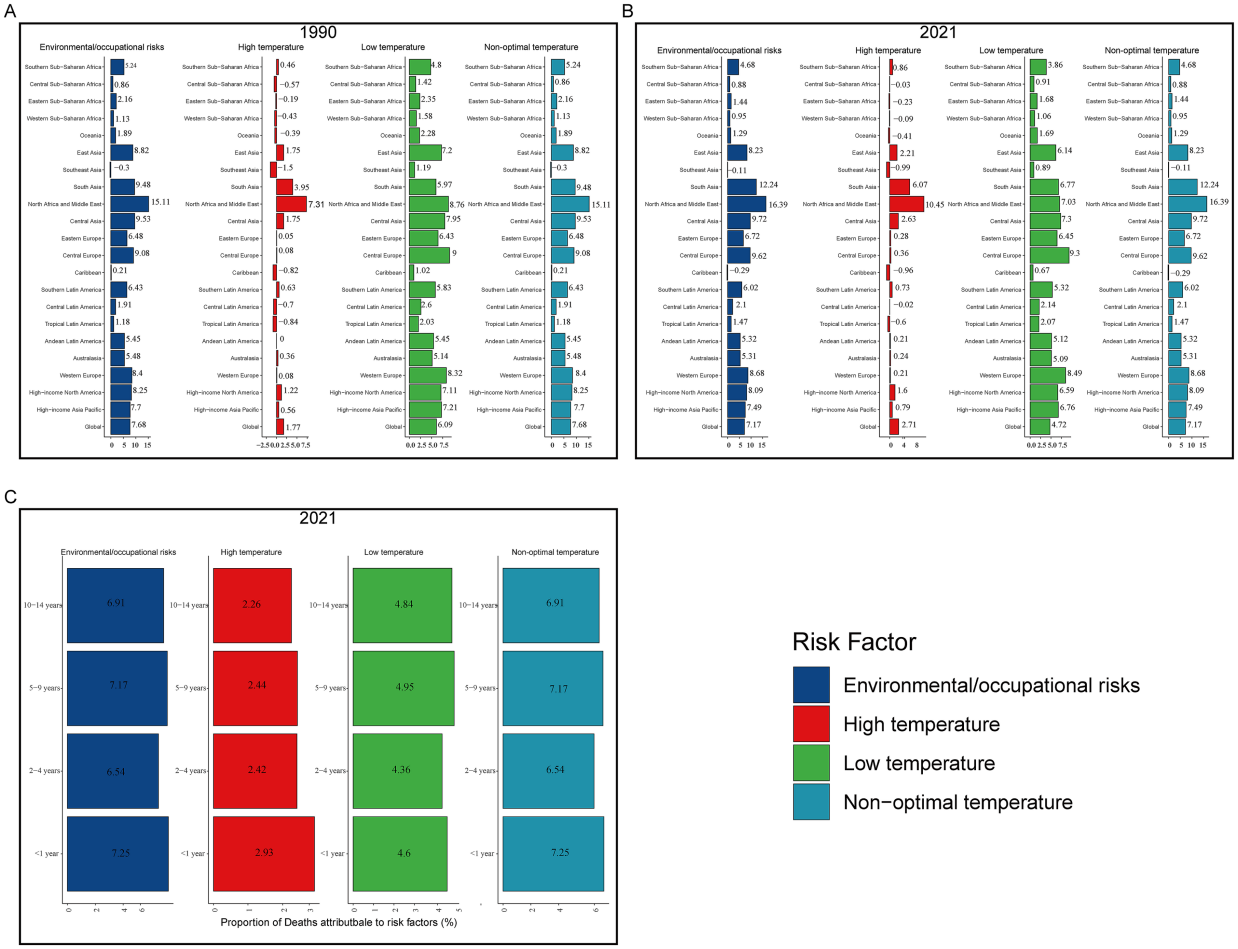
Proportion of myocarditis deaths attributable to risk factors. **(A)** Risk factors in 1990. **(B)** Risk factors in 2021. **(C)** Risk factors for different age groups in 2021.

The 2021 GBD database identified 4 risk factors for myocarditis in children: environmental/occupational risks, nonoptimal temperature, high temperature, and low temperature. In 1990, environmental/occupational risks accounted for 7.68% (486.73 of 6343.16) of myocarditis-associated deaths in children, while low temperatures contributed 6.09% (385.04 of 6343.16) and high temperatures 1.77% (113.12 of 6343.16) (Figure 7A). In 2021, environmental/occupational risks contributed to 7.17% (185.42 of 2593.47) of myocarditis-associated deaths in children. Among 21 regions, North Africa and the Middle East had the highest proportion of myocarditis-associated deaths attributable to environmental/occupational risks at 16.39% (24.08 of 166.12), with children under 1 year accounting for the largest share (7.25%). The proportion of myocarditis-associated deaths due to nonoptimal temperature was similar to that from environmental/occupational risks. Low temperatures were responsible for 4.72% (122.05 of 2593.47) of myocarditis-associated deaths, with Western Europe having the highest proportion (8.49%; 1.81 of 21.27), and the highest proportion of deaths occurred among children under 1 year (2.93%). High temperatures contributed to 2.71% (70.02 of 2593.47) of myocarditis-associated deaths, with the highest burden in North Africa and the Middle East (10.45%; 15.02 of 166.12), and children aged 5–9 years had the highest percentage of deaths (4.95%) (Figures 7B,C).

### 3.6 EAPC’s correlation factors

**Figure 8:**
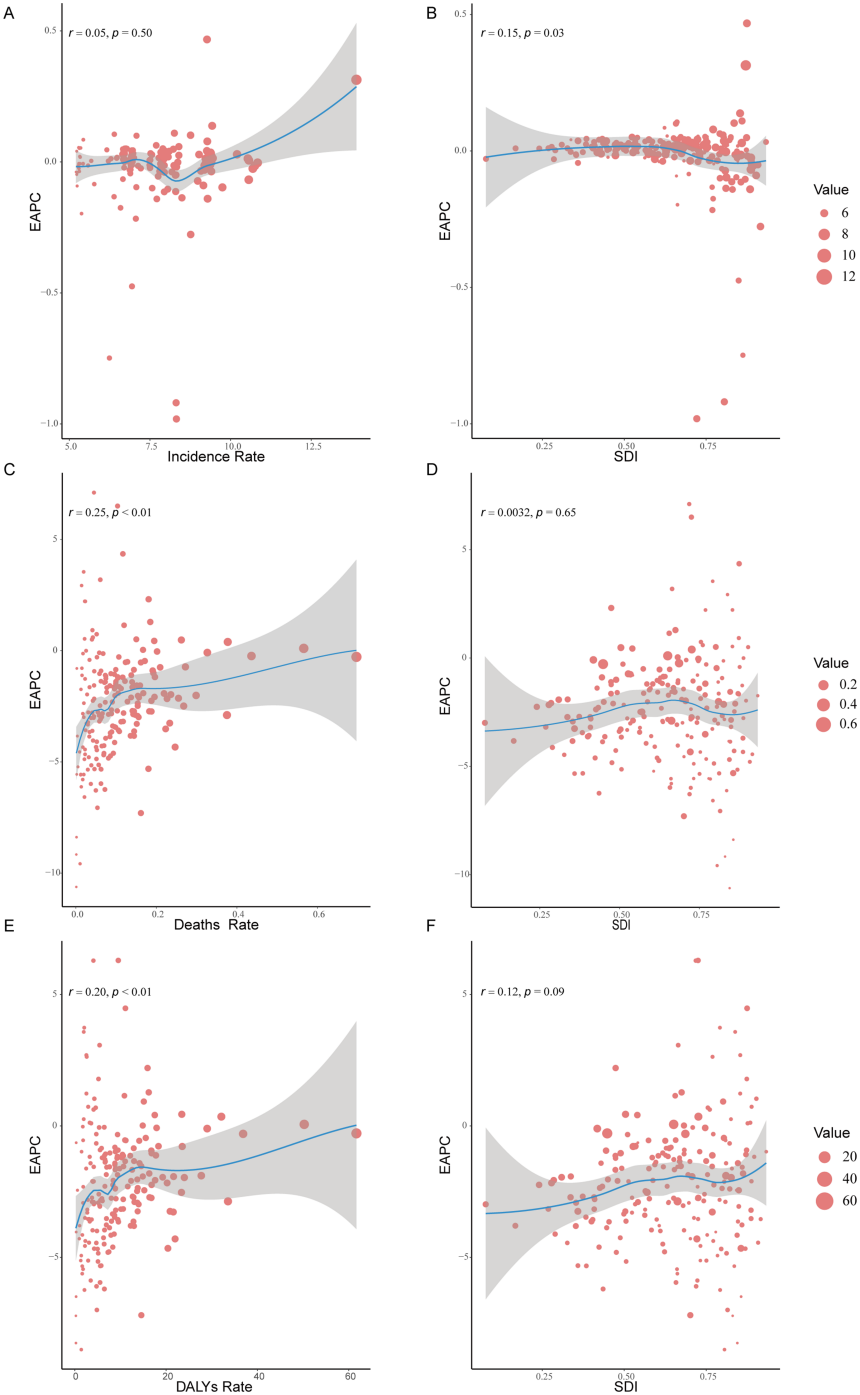
The correlation between EAPC and myocarditis incidence rate, deaths rate, and DALYs rate and SDI in 2021. **(A,C,E)** The correlation between EAPC and myocarditis incidence rate, deaths rate, and DALYs rate in 2021. **(B,D,F)** The correlation between EAPC and SDI in 2021. DALYs, disability-adjusted life-years; EAPCs, estimated annual percentage changes; SDI, sociodemographic index.

In 2021, there was no significant correlation between EAPC and the incidence rate of myocarditis in children (*r* = 0.05, *p* = 0.50). However, there was a weak positive correlation between EAPC in incidence rate and SDI (*r* = 0.15, *p* = 0.03). EAPC was weak positively correlated with myocarditis-associated mortality rate (*r* = 0.25, *p* < 0.01), though no correlation was found between EAPC in myocarditis-associated mortality rate and SDI (*r* = 0.0032, *p* = 0.65). There was also a weak positive correlation between myocarditis-associated DALY rate and EAPC (*r* = 0.20, *p* < 0.01), though EAPC in myocarditis-associated DALY rate was not correlated with SDI (*r* = 0.12, *p* = 0.09) (Figure 8).

## 4 Discussion

Over the past 30 years, the global incidence cases of myocarditis have increased, contributing to rising healthcare and societal costs, making it a significant public health issue that demands attention (7). This study analyzes data from the 2021 GBD database to assess the incidence, mortality, DALYs, and associated risk factors of myocarditis in children aged 0–14 years from 1990 to 2021. The findings aim to inform future prevention and treatment strategies for myocarditis in children. Our results present a comprehensive picture of the burden of myocarditis in children across different regions and countries, stratified by age and gender. While global trends show a decrease in the incidence, mortality, and DALYs, certain regions and countries have experienced an increasing burden of myocarditis in children. These findings provide valuable insights for policymakers and clinicians to formulate targeted prevention and management strategies to reduce the global burden of this condition.

From 1990 to 2021, the global incidence, mortality, and DALYs of myocarditis in children aged 0–14 years showed a downward trend. However, the cases of myocarditis in children increased by 8.10%. Our analysis revealed that males had a higher incidence rate of myocarditis than females, and the incidence rate increased with age, peaking in children aged 10–14 years, while being lowest in children aged under 1 year. The lower incidence rate in children under 1 year may be attributed to the atypical clinical presentation, making diagnosis more challenging (5, 17). Despite the lower incidence rate in children under 1 year, their myocarditis-associated mortality rate and DALYs rate were significantly higher compared to other age groups, likely due to delayed diagnosis and treatment, as well as their limited ability to cope with the disease (18). Previous studies have reported that 16–20% of sudden infant death syndrome (SIDS) cases are linked to myocarditis (19), underscoring the importance of early identification and timely intervention in this age group. This study also found that regions with Middle, High-Middle, and High SDI had a higher incidence rate of myocarditis in children than Low and Low-Middle SDI regions, but their myocarditis-associated mortality rate and DALYs rate were lower. This may be due to the limited medical conditions in Low and Low-Middle SDI regions, resulting in underdiagnosis of myocarditis, while Middle, High-Middle, and High SDI regions may have advanced instruments and equipment for early diagnosis and treatment. Therefore, more healthcare resources should be directed toward Low and Low-Middle SDI regions to reduce the burden of myocarditis in these areas. Among the 21 Geographic Regions, in 2021, High-income Asia Pacific had the highest incidence rate of myocarditis in children, while the Caribbean had the highest myocarditis-associated mortality rate and DALYs rate. East Asia had the highest number of myocarditis-associated death in children, with 0.66/1,000 people cases. This phenomenon may stem from the underdiagnosis of myocarditis in low-income regions, where access to healthcare resources is limited. In contrast, High-income regions are likely to possess advanced diagnostic technologies and robust healthcare infrastructures, which facilitate the early detection and management of the condition. Over the past 30 years, China decreased by 80.80% in myocarditis-associated deaths in children, reflecting improvements in healthcare and economic conditions. However, there were still 0.64/1000 people myocarditis-associated deaths (95% UI, 0.45–0.82) in 2021, maybe because of the large population base, indicating the need for continued investment in early diagnosis and treatment to further reduce mortality. In 2021, the incidence of myocarditis among children in Japan remained elevated (13.90; 95% UI, 9.25–20.41), potentially due to the resurgence of COVID-19, which may have contributed to the increased prevalence. However, the underlying factors require further investigation.

The exact etiology of myocarditis remains unclear, but various infectious agents, systemic diseases, drugs, and toxins can cause the condition (4). Acute lymphocytic myocarditis is the most common form and is often triggered by respiratory infections, such as those caused by adenoviruses (20). A 10-year autopsy study involving 12.75/1,000 people cases from the general population found that 1.06% had lymphocytic myocarditis (21). The clinical presentation of myocarditis varies widely, from mild chest pain and palpitations with transient electrocardiographic changes to life-threatening cardiogenic shock and ventricular arrhythmias (4). Common symptoms in children include abdominal pain, nausea, vomiting, and poor appetite (5, 22). Although myocarditis typically affects young individuals, it can occur at any age. Previous research has shown that myocarditis accounts for 16.00–20.00% of SIDS cases (19). The variability in clinical presentations makes diagnosis challenging, with EMB being the gold standard (23). However, due to its invasive nature, it is rarely used in clinical practice. Non-invasive methods such as cardiac MRI are now commonly used to diagnose myocarditis and monitor disease progression (24, 25). The low incidence rate of myocarditis in children may be underestimated, as many children present with mild symptoms, particularly neonates who cannot articulate their symptoms, leading clinicians to rely on physical examination and laboratory evaluations for diagnosis. This may explain the lower incidence rate in children under 1 year of myocarditis in this study. Myocarditis is a leading cause of sudden cardiac death (26). Among infants and adolescents, the prognosis of myocarditis in infants is particularly poor (5), consistent with our finding of higher myocarditis-associated mortality rate and DALYs rate in children under 1 year. Studies have also shown that myocarditis is more common in males than females (27), aligning with our results. Previous studies have demonstrated that the heart’s response to injury is influenced by sex hormones, which may help explain the higher incidence of myocarditis in men compared to women (28). In one such study, 3,198 patients with myocarditis were analyzed, of whom 77% were male. Experimental data further confirmed that sex hormones, determined by chromosomal differences, play a crucial role in the pathogenesis of the disease (29). However, myocarditis in females often presents with milder symptoms, leading to underdiagnosis (30). In murine models, male mice with Coxsackievirus B3 (CVB3)-induced myocarditis showed higher incidence and more severe clinical courses than female mice (31), differences in the innate immune response to CVB3 in male and female mice may explain this phenomenon (32, 33).

This study has several limitations. First, the analysis relies on data from the 2021 GBD database, and its accuracy depends on the availability of registry data across countries, undiagnosed myocarditis in children, and limited information on risk factors. Second, the varying clinical presentations of myocarditis and the focus on children aged 0–14 years across countries with different healthcare capabilities make accurate diagnosis challenging. Third, this study did not include specific classifications of myocarditis, which should be addressed in future research to better understand the condition in children. Finally, the results of this study indicate a reduction in mortality associated with myocarditis in children; however, the specific factors contributing to this decline remain unclear and require further investigation in future studies.

## 5 Conclusion

Between 1990 and 2021, global trends show a decline in the incidence, mortality, and DALYs associated with myocarditis in children aged 0 to 14 years. However, the overall incidence of myocarditis in this age group has been rising. Notably, the incidence rate in male children is higher than in females. Although the incidence among children under 1 year of age is relatively low, their mortality and DALY rates are significantly higher compared to other age groups. This highlights the critical importance of early diagnosis and timely treatment for myocarditis in infants under 1 year. From the perspective of the SDI, regions with Low and Low-Medium SDI have lower incidence rates of myocarditis in children, while regions with Medium, Medium-High, and High SDI exhibit higher incidence rates. Despite these higher incidence rates in the latter groups, the associated mortality and DALY rates remain relatively low. This pattern underscores the need for standardized treatment protocols in Low and Low-Medium SDI regions, where delays in diagnosis and treatment could exacerbate the burden of the disease. Future investment and support should prioritize the High-income Asia-Pacific region, which bears a disproportionately high burden of myocarditis. Notably, Japan reported the highest incidence of myocarditis in children in 2021, indicating a need for increased medical resources to investigate the underlying causes and implement preventive measures to reduce the disease burden. Additionally, China recorded the highest number of myocarditis-related deaths among children in 2021, emphasizing the urgency of early, standardized treatment and the improvement of diagnostic and therapeutic protocols. Further research into the etiological factors of myocarditis is essential. Such investigations will not only enhance the accuracy of disease burden estimates but also provide a scientific foundation for developing more targeted and effective prevention and control strategies.

## Data Availability

Publicly available datasets were analyzed in this study. This data can be found at: https://vizhub.healthdata.org/gbd-results/.

## Footnotes

^1^ https://vizhub.healthdata.org/gbd-results/

^2^ https://ghdx.healthdata.org/search/site/SDI
