# Supplementary material for: Global, regional, and national burden of myocarditis in children aged 0–14 years, 1990–2021: analysis for the global burden of disease study 2021
Source: Front Public Health. 2024 Dec 20;12:1504586. doi: 10.3389/fpubh.2024.1504586 (PMC11695415; doi:10.3389/fpubh.2024.1504586)
Supplement: Supplementary file 1 [file Table_1.DOCX]

Supplementary Material

# Supplementary Tables

Supplementary Table 1. Incidence of Myocarditis in Children Between 1990 and 2021 at the National Level

|  | **Rate per 100000(95%UI)** |  |  |  |  |  |
| --- | --- | --- | --- | --- | --- | --- |
|  | **1990** | | **2021** | | **1990-2021** | |
| **Location** | **Incident cases** | **Incident rate** | **Incident cases** | **Incident rate** | **cases change** | **EAPCs** |
| Afghanistan | 227.00(143.90-348.22) | 5.27(3.34-8.08) | 742.68(467.11-1149.23) | 5.23(3.29-8.09) | 227.17(222.30-231.01) | 0.04(0.02-0.06) |
| Albania | 83.60(56.20-120.70) | 7.48(5.03-10.80) | 33.36(22.22-48.56) | 7.52(5.01-10.94) | -60.09(-61.10--59.29) | 0.03(0.02-0.04) |
| Algeria | 564.88(352.48-880.97) | 5.27(3.29-8.21) | 697.34(434.89-1088.43) | 5.24(3.27-8.18) | 23.45(23.22-23.61) | -0.09(-0.13--0.06) |
| American Samoa | 1.74(1.16-2.56) | 9.16(6.08-13.47) | 1.31(0.84-1.99) | 9.27(5.92-14.07) | -24.58(-31.09--19.62) | 0.04(0.04-0.05) |
| Andorra | 0.78(0.52-1.14) | 8.21(5.51-11.97) | 0.82(0.55-1.20) | 8.05(5.39-11.82) | 4.96(2.13-7.29) | -0.10(-0.13--0.07) |
| Angola | 322.96(211.61-470.02) | 6.85(4.49-9.97) | 1048.89(679.41-1549.01) | 6.88(4.46-10.16) | 224.77(215.78-231.71) | 0.01(-0.00-0.01) |
| Antigua and Barbuda | 1.21(0.77-1.86) | 6.64(4.23-10.24) | 1.13(0.72-1.75) | 6.69(4.24-10.33) | -6.43(-8.00--5.11) | 0.04(0.04-0.05) |
| Argentina | 574.20(368.45-849.61) | 5.67(3.64-8.38) | 578.42(366.61-877.23) | 5.68(3.60-8.61) | 0.73(-2.16-2.82) | 0.00(0.00-0.01) |
| Armenia | 92.34(60.14-134.96) | 8.85(5.76-12.94) | 53.44(34.10-79.72) | 9.02(5.76-13.46) | -42.13(-45.29--37.75) | 0.03(-0.00-0.06) |
| Australia | 253.93(164.84-370.53) | 6.71(4.35-9.79) | 318.81(205.45-468.62) | 6.71(4.33-9.87) | 25.55(24.17-26.50) | -0.00(-0.00-0.00) |
| Austria | 125.23(91.88-167.62) | 9.29(6.81-12.43) | 122.30(85.57-171.25) | 9.43(6.60-13.20) | -2.34(-12.71-9.14) | 0.14(-0.06-0.34) |
| Azerbaijan | 192.58(124.28-292.11) | 7.94(5.12-12.04) | 191.18(120.93-295.58) | 8.10(5.12-12.52) | -0.73(-4.61-2.16) | 0.04(0.01-0.07) |
| Bahamas | 5.37(3.40-8.30) | 6.66(4.22-10.29) | 5.48(3.44-8.57) | 6.75(4.24-10.56) | 2.06(-2.23-5.26) | 0.05(0.04-0.06) |
| Bahrain | 8.49(5.32-13.20) | 5.20(3.26-8.09) | 15.76(9.77-24.61) | 5.31(3.29-8.29) | 85.60(78.26-90.73) | 0.05(0.04-0.06) |
| Bangladesh | 3373.64(2188.90-4958.09) | 6.90(4.48-10.14) | 3194.98(2020.67-4776.21) | 6.98(4.42-10.44) | -5.30(-9.66--2.19) | 0.02(0.01-0.03) |
| Barbados | 4.16(2.64-6.43) | 6.68(4.23-10.31) | 3.17(2.00-4.91) | 6.73(4.24-10.43) | -23.89(-25.33--22.92) | 0.03(0.02-0.04) |
| Belarus | 186.74(121.64-283.01) | 7.77(5.06-11.77) | 123.10(78.94-188.07) | 7.80(5.00-11.92) | -34.08(-35.58--33.08) | -0.04(-0.06--0.01) |
| Belgium | 129.96(88.01-184.46) | 7.20(4.87-10.21) | 136.35(92.12-194.84) | 7.13(4.82-10.19) | 4.92(3.48-6.22) | -0.01(-0.03-0.00) |
| Belize | 5.41(3.45-8.29) | 6.60(4.22-10.12) | 8.22(5.20-12.70) | 6.68(4.22-10.32) | 52.08(46.84-55.82) | 0.04(0.03-0.04) |
| Benin | 166.64(108.78-243.56) | 6.88(4.49-10.06) | 418.67(271.73-615.21) | 6.89(4.47-10.12) | 151.25(147.10-154.49) | -0.00(-0.01--0.00) |
| Bermuda | 0.79(0.50-1.21) | 6.62(4.23-10.14) | 0.57(0.36-0.87) | 6.70(4.24-10.36) | -28.34(-31.01--26.46) | 0.04(0.03-0.04) |
| Bhutan | 18.23(11.83-26.89) | 6.95(4.51-10.26) | 13.03(8.28-19.39) | 6.96(4.43-10.36) | -28.50(-30.25--27.28) | 0.00(-0.00-0.00) |
| Bolivia (Plurinational State of) | 172.70(111.56-261.12) | 6.43(4.15-9.72) | 225.50(143.64-344.44) | 6.47(4.12-9.88) | 30.58(27.72-32.52) | 0.01(0.01-0.02) |
| Bosnia and Herzegovina | 82.01(54.53-119.42) | 7.49(4.98-10.90) | 36.75(24.41-53.67) | 7.49(4.98-10.94) | -55.18(-55.59--54.88) | 0.01(-0.00-0.02) |
| Botswana | 40.72(26.39-60.18) | 6.90(4.47-10.19) | 48.53(31.04-72.14) | 6.95(4.44-10.33) | 19.18(17.28-20.51) | 0.02(0.01-0.02) |
| Brazil | 3912.77(2419.18-5990.48) | 7.53(4.66-11.53) | 3615.17(2259.23-5513.81) | 7.50(4.69-11.44) | -7.61(-8.52--6.27) | -0.02(-0.03--0.01) |
| Brunei Darussalam | 9.85(6.79-13.93) | 10.88(7.49-15.38) | 10.25(6.95-14.69) | 10.84(7.35-15.53) | 4.06(0.48-7.02) | -0.00(-0.02-0.01) |
| Bulgaria | 130.39(86.88-190.03) | 7.51(5.00-10.94) | 73.22(48.72-106.76) | 7.50(4.99-10.94) | -43.85(-44.04--43.67) | -0.02(-0.03--0.01) |
| Burkina Faso | 326.93(213.46-477.27) | 6.93(4.52-10.11) | 712.34(463.68-1042.99) | 6.87(4.47-10.06) | 117.88(116.29-119.04) | -0.03(-0.03--0.03) |
| Burundi | 179.34(117.22-261.69) | 6.84(4.47-9.98) | 403.02(261.50-594.69) | 6.88(4.47-10.16) | 124.73(118.64-129.22) | 0.00(-0.01-0.01) |
| Cabo Verde | 10.83(7.01-15.96) | 6.88(4.46-10.14) | 10.00(6.32-14.97) | 6.99(4.41-10.46) | -7.60(-11.72--4.65) | 0.03(0.02-0.04) |
| Cambodia | 432.61(286.18-638.27) | 9.28(6.14-13.69) | 479.88(311.72-714.52) | 9.38(6.09-13.96) | 10.93(7.29-13.70) | 0.02(0.01-0.04) |
| Cameroon | 335.69(219.43-489.47) | 6.88(4.49-10.03) | 932.16(604.14-1378.55) | 6.92(4.49-10.24) | 177.69(168.87-184.28) | 0.01(0.00-0.02) |
| Canada | 354.69(236.02-529.44) | 6.17(4.10-9.20) | 383.30(253.27-578.58) | 6.21(4.10-9.37) | 8.06(6.19-9.44) | 0.00(-0.01-0.02) |
| Central African Republic | 83.86(54.85-122.23) | 6.86(4.49-10.00) | 157.86(102.51-232.82) | 6.91(4.49-10.19) | 88.25(82.88-92.22) | 0.02(0.02-0.03) |
| Chad | 200.41(131.98-290.73) | 6.85(4.51-9.93) | 618.68(403.43-904.46) | 6.86(4.48-10.03) | 208.71(203.98-212.40) | 0.01(0.00-0.01) |
| Chile | 277.99(184.55-414.10) | 7.00(4.65-10.43) | 240.58(164.51-340.85) | 6.59(4.50-9.33) | -13.46(-20.84--5.53) | -0.18(-0.23--0.12) |
| China | 33088.61(21137.16-48844.70) | 10.39(6.64-15.34) | 21603.18(14078.21-31928.87) | 8.32(5.42-12.30) | -34.71(-37.18--32.16) | -0.98(-1.16--0.80) |
| Colombia | 771.60(493.51-1181.41) | 6.62(4.23-10.13) | 708.42(449.88-1092.20) | 6.67(4.24-10.29) | -8.19(-10.46--6.60) | 0.04(0.03-0.05) |
| Comoros | 14.67(9.55-21.51) | 6.90(4.49-10.11) | 16.70(10.69-24.82) | 6.95(4.45-10.33) | 13.80(10.17-16.45) | 0.03(0.03-0.03) |
| Congo | 72.50(47.12-106.70) | 6.89(4.48-10.13) | 134.07(85.29-199.78) | 6.95(4.42-10.36) | 84.92(79.50-88.92) | 0.02(0.01-0.03) |
| Cook Islands | 0.61(0.39-0.91) | 9.18(5.92-13.75) | 0.35(0.22-0.52) | 9.22(5.92-13.84) | -42.34(-43.92--41.27) | 0.01(0.01-0.02) |
| Costa Rica | 74.39(47.55-114.08) | 6.62(4.23-10.15) | 68.03(42.86-105.41) | 6.69(4.21-10.36) | -8.55(-11.98--6.22) | 0.01(0.00-0.02) |
| Croatia | 67.70(43.88-99.58) | 6.86(4.45-10.09) | 39.78(27.08-56.35) | 6.66(4.53-9.44) | -41.23(-46.62--36.10) | -0.07(-0.27-0.14) |
| Cuba | 166.36(106.36-254.94) | 6.64(4.25-10.18) | 118.93(75.03-184.18) | 6.69(4.22-10.36) | -28.51(-30.67--27.07) | 0.01(0.00-0.03) |
| Cyprus | 17.22(11.93-23.88) | 8.70(6.03-12.07) | 19.16(13.32-26.44) | 8.76(6.09-12.09) | 11.26(10.29-12.34) | 0.10(0.06-0.14) |
| Czechia | 183.78(121.66-275.28) | 8.34(5.52-12.49) | 142.43(95.45-211.44) | 8.30(5.56-12.32) | -22.50(-23.92--20.70) | -0.11(-0.18--0.05) |
| Côte d'Ivoire | 392.52(256.20-573.40) | 6.88(4.49-10.05) | 800.64(519.10-1179.62) | 6.92(4.49-10.19) | 103.98(99.69-107.18) | 0.01(0.00-0.01) |
| Democratic People's Republic of Korea | 497.34(340.32-712.63) | 8.36(5.72-11.98) | 399.23(266.34-580.71) | 8.36(5.58-12.16) | -19.73(-23.73--16.64) | -0.00(-0.00-0.00) |
| Democratic Republic of the Congo | 1213.56(793.91-1768.44) | 6.85(4.48-9.99) | 2631.04(1701.92-3895.74) | 6.92(4.48-10.25) | 116.80(109.24-122.60) | 0.02(0.02-0.03) |
| Denmark | 68.56(47.42-98.00) | 7.76(5.37-11.09) | 73.99(51.15-105.70) | 7.75(5.36-11.08) | 7.91(7.46-8.26) | -0.05(-0.11--0.00) |
| Djibouti | 12.13(7.89-17.84) | 6.97(4.53-10.25) | 28.96(18.69-42.91) | 7.01(4.52-10.39) | 138.75(135.69-141.04) | 0.02(0.01-0.02) |
| Dominica | 1.65(1.06-2.54) | 6.66(4.26-10.23) | 0.92(0.58-1.45) | 6.76(4.23-10.59) | -44.07(-47.38--41.89) | 0.05(0.05-0.06) |
| Dominican Republic | 177.83(113.86-271.30) | 6.60(4.22-10.07) | 194.86(124.43-299.46) | 6.63(4.23-10.19) | 9.57(8.30-10.45) | 0.03(0.02-0.04) |
| Ecuador | 243.35(157.35-363.26) | 6.30(4.07-9.40) | 324.27(208.74-477.37) | 6.39(4.12-9.41) | 33.25(24.63-42.11) | 0.11(0.08-0.14) |
| Egypt | 1156.94(726.90-1790.93) | 5.22(3.28-8.07) | 1935.09(1206.88-3016.86) | 5.25(3.27-8.19) | 67.26(64.37-69.32) | -0.04(-0.06--0.02) |
| El Salvador | 143.00(91.43-219.06) | 6.63(4.24-10.15) | 121.11(76.92-186.94) | 6.66(4.23-10.28) | -15.30(-16.73--14.34) | 0.03(0.02-0.05) |
| Equatorial Guinea | 13.52(8.88-19.67) | 6.87(4.51-9.99) | 41.44(26.25-61.95) | 7.08(4.49-10.59) | 206.39(187.09-221.04) | 0.10(0.10-0.10) |
| Eritrea | 110.10(71.61-161.31) | 6.92(4.50-10.13) | 175.17(113.58-258.81) | 6.94(4.50-10.25) | 59.11(56.24-61.27) | 0.01(0.01-0.01) |
| Estonia | 27.12(17.76-40.95) | 7.77(5.09-11.73) | 16.89(10.92-25.59) | 7.82(5.05-11.84) | -37.72(-38.80--36.92) | -0.03(-0.05-0.00) |
| Eswatini | 26.53(17.22-39.06) | 6.88(4.46-10.13) | 28.75(18.42-42.73) | 6.97(4.46-10.35) | 8.38(5.76-10.24) | 0.03(0.03-0.04) |
| Ethiopia | 1891.47(1218.32-2852.50) | 7.76(5.00-11.71) | 3474.87(2221.18-5292.68) | 7.84(5.01-11.93) | 83.71(79.30-86.74) | 0.04(0.03-0.04) |
| Fiji | 25.99(16.82-38.87) | 9.23(5.98-13.81) | 25.17(16.28-37.62) | 9.23(5.97-13.81) | -3.16(-3.31--2.97) | -0.01(-0.01--0.01) |
| Finland | 80.89(55.46-115.80) | 8.38(5.75-12.00) | 69.94(47.45-100.78) | 8.26(5.60-11.90) | -13.53(-15.62--11.68) | 0.11(0.01-0.21) |
| France | 976.36(661.24-1405.96) | 8.33(5.64-12.00) | 956.87(644.55-1385.41) | 8.24(5.55-11.94) | -2.00(-3.83--0.43) | -0.01(-0.03-0.00) |
| Gabon | 28.00(18.19-41.14) | 6.87(4.46-10.10) | 44.18(28.21-65.77) | 6.91(4.41-10.29) | 57.77(52.97-61.24) | 0.01(0.00-0.02) |
| Gambia | 31.64(20.64-46.23) | 6.86(4.47-10.02) | 68.71(44.51-101.55) | 6.92(4.48-10.22) | 117.18(111.07-121.64) | 0.02(0.01-0.03) |
| Georgia | 98.37(63.17-145.37) | 7.19(4.62-10.62) | 53.10(33.51-78.98) | 7.22(4.55-10.73) | -46.02(-49.02--43.44) | -0.02(-0.05-0.00) |
| Germany | 1189.22(825.18-1697.60) | 9.19(6.37-13.11) | 1095.74(760.14-1569.69) | 9.16(6.35-13.12) | -7.86(-8.33--7.44) | -0.09(-0.19-0.01) |
| Ghana | 463.85(301.81-679.59) | 6.91(4.49-10.12) | 892.14(577.90-1320.01) | 6.92(4.49-10.25) | 92.33(88.02-95.49) | 0.00(0.00-0.00) |
| Greece | 153.13(103.86-220.75) | 7.57(5.13-10.91) | 107.32(73.42-155.00) | 7.69(5.26-11.11) | -29.92(-33.42--25.73) | 0.06(0.04-0.08) |
| Greenland | 0.87(0.58-1.28) | 6.10(4.06-8.98) | 0.72(0.48-1.08) | 6.16(4.10-9.19) | -16.59(-19.45--14.32) | 0.05(0.02-0.07) |
| Grenada | 2.21(1.41-3.39) | 6.61(4.23-10.15) | 1.46(0.93-2.26) | 6.69(4.24-10.33) | -33.84(-35.84--32.40) | 0.05(0.04-0.05) |
| Guam | 3.86(2.56-5.66) | 9.25(6.15-13.58) | 3.40(2.22-5.04) | 9.30(6.07-13.79) | -11.79(-14.24--9.96) | 0.04(0.03-0.04) |
| Guatemala | 266.98(171.29-406.71) | 6.57(4.22-10.01) | 328.51(207.88-508.44) | 6.66(4.21-10.30) | 23.05(18.12-26.50) | 0.05(0.04-0.05) |
| Guinea | 190.06(125.17-275.69) | 6.91(4.55-10.02) | 417.45(270.63-615.54) | 6.90(4.48-10.18) | 119.64(111.97-125.61) | -0.00(-0.01-0.00) |
| Guinea-Bissau | 33.31(21.70-48.76) | 6.91(4.50-10.11) | 62.02(40.22-91.50) | 6.91(4.48-10.19) | 86.18(82.89-88.68) | -0.01(-0.02--0.01) |
| Guyana | 19.65(12.73-29.60) | 6.69(4.33-10.07) | 14.27(9.12-21.87) | 6.69(4.27-10.25) | -27.40(-29.17--26.14) | 0.05(0.02-0.07) |
| Haiti | 181.94(118.00-272.93) | 6.71(4.35-10.06) | 291.73(186.94-444.25) | 6.70(4.29-10.21) | 60.34(56.38-63.29) | 0.00(-0.00-0.01) |
| Honduras | 145.77(93.25-222.49) | 6.60(4.22-10.07) | 218.05(138.76-335.88) | 6.65(4.23-10.25) | 49.58(45.78-52.32) | 0.03(0.03-0.03) |
| Hungary | 160.74(106.22-235.81) | 7.54(4.98-11.07) | 104.02(69.32-151.18) | 7.49(4.99-10.89) | -35.29(-36.75--33.42) | -0.01(-0.02--0.00) |
| Iceland | 5.30(3.60-7.62) | 8.35(5.67-12.02) | 5.62(3.81-8.11) | 8.33(5.64-12.01) | 6.09(5.09-7.04) | -0.02(-0.04--0.00) |
| India | 25623.27(16376.01-39163.75) | 7.85(5.02-11.99) | 29226.62(18278.11-44779.53) | 7.98(4.99-12.22) | 14.06(10.22-16.83) | 0.04(0.04-0.05) |
| Indonesia | 7142.83(4598.12-10741.22) | 10.54(6.79-15.86) | 7109.67(4580.30-10687.89) | 10.57(6.81-15.88) | -0.46(-0.86--0.09) | 0.01(0.00-0.01) |
| Iran (Islamic Republic of) | 1444.59(897.68-2197.97) | 5.69(3.54-8.66) | 1165.03(722.75-1791.04) | 5.77(3.58-8.88) | -19.35(-21.54--17.90) | -0.08(-0.13--0.04) |
| Iraq | 468.08(298.99-704.26) | 5.68(3.63-8.55) | 724.12(450.81-1126.74) | 5.38(3.35-8.37) | 54.70(42.42-64.05) | -0.20(-0.21--0.19) |
| Ireland | 80.71(54.21-117.42) | 8.21(5.52-11.95) | 82.02(55.17-119.04) | 8.23(5.53-11.94) | 1.63(1.34-1.95) | 0.05(0.01-0.09) |
| Israel | 128.13(86.88-184.32) | 8.36(5.67-12.02) | 220.62(149.95-316.73) | 8.40(5.71-12.05) | 72.19(70.94-73.67) | 0.03(0.02-0.04) |
| Italy | 941.57(606.50-1404.58) | 10.20(6.57-15.22) | 631.63(439.31-877.41) | 8.31(5.78-11.55) | -32.92(-38.52--26.94) | -0.92(-1.15--0.69) |
| Jamaica | 55.44(35.27-85.46) | 6.64(4.22-10.23) | 39.18(24.72-60.69) | 6.71(4.23-10.39) | -29.34(-31.33--27.95) | 0.04(0.03-0.05) |
| Japan | 2979.30(1923.11-4427.89) | 12.90(8.33-19.18) | 2146.93(1429.15-3152.48) | 13.90(9.25-20.41) | -27.94(-30.14--24.88) | 0.31(0.28-0.35) |
| Jordan | 85.62(53.65-132.93) | 5.24(3.28-8.14) | 194.37(120.48-302.72) | 5.35(3.32-8.33) | 127.02(117.28-133.76) | 0.05(0.04-0.07) |
| Kazakhstan | 412.17(265.78-627.47) | 7.93(5.11-12.08) | 431.48(278.09-656.88) | 7.95(5.12-12.11) | 4.69(4.53-4.81) | -0.04(-0.07--0.01) |
| Kenya | 866.43(557.87-1311.25) | 7.76(4.99-11.74) | 1474.53(927.02-2258.25) | 7.90(4.97-12.10) | 70.18(63.24-74.89) | 0.05(0.04-0.06) |
| Kiribati | 2.72(1.81-4.00) | 9.22(6.12-13.55) | 3.87(2.51-5.78) | 9.22(5.98-13.77) | 42.30(36.46-46.80) | -0.01(-0.01--0.00) |
| Kuwait | 29.08(18.20-45.23) | 5.25(3.28-8.16) | 45.12(27.98-70.39) | 5.34(3.31-8.33) | 55.16(50.22-58.58) | 0.02(-0.00-0.04) |
| Kyrgyzstan | 132.47(85.75-199.99) | 7.90(5.11-11.92) | 180.73(116.01-276.23) | 7.95(5.10-12.15) | 36.43(33.79-38.34) | -0.01(-0.04-0.02) |
| Lao People's Democratic Republic | 171.47(113.17-252.94) | 9.30(6.14-13.72) | 214.62(139.95-318.50) | 9.35(6.09-13.87) | 25.17(23.03-26.76) | 0.02(0.01-0.03) |
| Latvia | 43.58(28.54-63.16) | 7.66(5.02-11.10) | 22.91(14.95-33.64) | 7.71(5.03-11.33) | -47.42(-48.76--46.47) | -0.14(-0.26--0.03) |
| Lebanon | 55.14(34.62-85.38) | 5.27(3.31-8.16) | 68.11(42.23-106.20) | 5.33(3.30-8.31) | 23.52(19.04-26.56) | -0.01(-0.02-0.01) |
| Lesotho | 47.14(30.56-69.70) | 6.91(4.48-10.21) | 43.88(27.84-65.42) | 6.96(4.42-10.38) | -6.92(-9.22--5.31) | 0.02(0.02-0.02) |
| Liberia | 78.27(51.43-113.82) | 6.93(4.55-10.07) | 151.73(97.82-224.80) | 6.94(4.48-10.28) | 93.85(84.97-100.44) | -0.01(-0.03-0.00) |
| Libya | 98.85(62.45-152.16) | 5.46(3.45-8.40) | 80.94(50.33-126.00) | 5.43(3.37-8.45) | -18.11(-23.23--14.63) | -0.01(-0.02-0.01) |
| Lithuania | 65.80(43.02-96.58) | 7.92(5.18-11.63) | 32.40(20.89-47.82) | 7.94(5.12-11.73) | -50.77(-51.57--50.24) | -0.11(-0.18--0.05) |
| Luxembourg | 6.18(4.30-8.56) | 9.35(6.51-12.96) | 9.40(6.53-13.08) | 9.29(6.45-12.92) | 52.16(49.94-54.09) | -0.14(-0.21--0.07) |
| Madagascar | 375.85(244.84-549.79) | 6.89(4.49-10.08) | 812.31(523.05-1205.19) | 6.92(4.46-10.27) | 116.13(109.58-121.10) | 0.02(0.01-0.02) |
| Malawi | 311.26(204.57-452.33) | 6.84(4.50-9.94) | 563.39(359.96-837.24) | 6.94(4.43-10.31) | 81.00(71.50-88.15) | 0.04(0.03-0.06) |
| Malaysia | 614.53(401.00-912.67) | 9.35(6.10-13.89) | 716.56(463.62-1069.54) | 9.41(6.09-14.05) | 16.60(13.57-18.80) | 0.02(0.02-0.03) |
| Maldives | 9.76(6.46-14.40) | 9.29(6.15-13.71) | 9.42(6.09-14.11) | 9.41(6.08-14.08) | -3.47(-8.17-0.13) | 0.02(-0.00-0.04) |
| Mali | 283.43(186.30-412.03) | 6.86(4.51-9.98) | 796.21(518.12-1166.36) | 6.88(4.48-10.08) | 180.92(176.18-184.53) | 0.01(0.01-0.01) |
| Malta | 7.28(4.92-10.50) | 8.32(5.62-12.00) | 5.37(3.65-7.72) | 8.40(5.70-12.07) | -26.16(-27.03--25.14) | 0.06(0.03-0.09) |
| Marshall Islands | 2.02(1.32-3.03) | 9.23(6.00-13.81) | 1.61(1.04-2.41) | 9.23(5.95-13.82) | -20.38(-21.58--19.36) | -0.01(-0.02--0.01) |
| Mauritania | 63.62(41.48-93.00) | 6.88(4.49-10.06) | 128.23(82.84-190.01) | 6.92(4.47-10.25) | 101.55(95.49-106.05) | 0.01(-0.00-0.01) |
| Mauritius | 31.01(20.07-46.24) | 9.40(6.08-14.01) | 19.52(12.63-29.18) | 9.41(6.09-14.07) | -37.05(-37.57--36.69) | 0.02(0.02-0.03) |
| Mexico | 2497.97(1567.25-3797.92) | 7.48(4.69-11.37) | 2424.75(1494.01-3708.79) | 7.56(4.66-11.57) | -2.93(-5.47--1.17) | 0.03(0.02-0.03) |
| Micronesia (Federated States of) | 4.26(2.77-6.35) | 9.27(6.03-13.84) | 2.83(1.82-4.24) | 9.25(5.94-13.86) | -33.53(-35.21--32.37) | -0.01(-0.01--0.01) |
| Monaco | 0.30(0.20-0.43) | 8.40(5.70-12.08) | 0.41(0.28-0.59) | 8.29(5.61-11.94) | 39.44(38.61-40.24) | -0.05(-0.06--0.04) |
| Mongolia | 71.18(46.04-107.63) | 7.91(5.11-11.96) | 86.02(55.30-131.30) | 7.92(5.09-12.08) | 20.85(19.56-21.93) | -0.04(-0.07--0.01) |
| Montenegro | 12.09(8.04-17.60) | 7.48(4.98-10.89) | 8.36(5.57-12.16) | 7.51(5.00-10.92) | -30.84(-30.99--30.64) | 0.01(0.00-0.01) |
| Morocco | 519.48(326.67-803.57) | 5.31(3.34-8.21) | 518.95(322.97-807.93) | 5.30(3.30-8.25) | -0.10(-3.13-1.95) | -0.03(-0.03--0.02) |
| Mozambique | 426.01(276.93-624.68) | 6.87(4.46-10.07) | 983.20(636.82-1454.22) | 6.89(4.46-10.19) | 130.79(126.85-133.81) | 0.01(0.00-0.02) |
| Myanmar | 1381.47(897.61-2056.84) | 9.35(6.07-13.92) | 1463.99(948.71-2179.97) | 9.38(6.08-13.96) | 5.97(5.37-6.39) | 0.01(0.00-0.01) |
| Namibia | 41.39(26.91-60.87) | 6.89(4.48-10.13) | 57.25(36.61-85.10) | 6.94(4.44-10.31) | 38.32(34.85-40.82) | 0.02(0.01-0.02) |
| Nauru | 0.39(0.26-0.57) | 9.19(6.07-13.55) | 0.37(0.24-0.55) | 9.25(6.03-13.75) | -5.17(-7.60--3.40) | 0.01(0.01-0.02) |
| Nepal | 581.39(378.09-852.02) | 6.90(4.49-10.11) | 643.22(411.40-955.34) | 6.97(4.46-10.35) | 10.63(6.72-13.48) | 0.04(0.04-0.05) |
| Netherlands | 228.27(155.18-328.26) | 8.38(5.69-12.04) | 222.77(150.71-321.44) | 8.31(5.62-11.98) | -2.41(-3.76--1.23) | -0.05(-0.06--0.04) |
| New Zealand | 59.60(36.99-88.77) | 7.45(4.62-11.09) | 68.16(43.71-100.44) | 6.94(4.45-10.23) | 14.37(6.85-22.69) | -0.47(-0.61--0.34) |
| Nicaragua | 120.15(76.75-184.05) | 6.60(4.21-10.11) | 131.94(83.80-203.58) | 6.66(4.23-10.28) | 9.81(7.16-11.65) | 0.02(0.01-0.03) |
| Niger | 278.18(182.67-404.69) | 6.85(4.50-9.96) | 877.02(571.22-1283.66) | 6.87(4.48-10.06) | 215.27(210.74-218.76) | 0.01(0.00-0.02) |
| Nigeria | 3016.50(1958.93-4535.19) | 7.71(5.01-11.59) | 7899.16(5060.25-12015.03) | 7.78(4.98-11.83) | 161.86(154.11-167.83) | 0.02(0.01-0.02) |
| Niue | 0.07(0.05-0.11) | 9.24(5.93-13.93) | 0.04(0.02-0.05) | 9.32(5.99-13.98) | -51.62(-52.35--50.82) | 0.01(-0.01-0.03) |
| North Macedonia | 39.41(26.21-57.38) | 7.48(4.97-10.89) | 24.54(16.28-35.86) | 7.49(4.97-10.95) | -37.74(-38.41--37.25) | 0.01(-0.00-0.01) |
| Northern Mariana Islands | 1.10(0.73-1.62) | 9.08(6.02-13.30) | 1.05(0.67-1.58) | 9.28(5.95-13.99) | -5.29(-12.03--0.33) | 0.08(0.06-0.09) |
| Norway | 70.17(45.47-103.09) | 8.79(5.70-12.91) | 80.95(51.48-121.80) | 8.76(5.57-13.18) | 15.37(12.21-17.85) | -0.28(-0.47--0.08) |
| Oman | 43.76(27.54-67.73) | 5.21(3.28-8.06) | 63.97(39.80-100.15) | 5.23(3.25-8.19) | 46.17(41.98-49.13) | -0.05(-0.08--0.01) |
| Pakistan | 3851.60(2476.07-5847.24) | 7.82(5.03-11.87) | 6722.61(4280.77-10288.93) | 7.87(5.01-12.04) | 74.54(71.45-76.64) | 0.01(0.01-0.02) |
| Palau | 0.42(0.27-0.63) | 9.27(5.99-13.86) | 0.30(0.19-0.45) | 9.27(5.95-13.93) | -28.43(-30.29--27.16) | 0.01(0.01-0.02) |
| Palestine | 50.26(31.70-77.59) | 5.19(3.27-8.01) | 98.82(61.41-154.12) | 5.29(3.29-8.25) | 96.63(87.49-102.93) | 0.05(0.05-0.06) |
| Panama | 55.37(35.29-85.23) | 6.64(4.23-10.22) | 76.88(48.73-118.84) | 6.67(4.22-10.30) | 38.84(37.00-40.03) | 0.01(0.00-0.01) |
| Papua New Guinea | 159.93(106.53-234.96) | 9.41(6.27-13.82) | 367.42(244.95-539.36) | 9.38(6.25-13.77) | 129.74(129.23-130.41) | -0.00(-0.01--0.00) |
| Paraguay | 110.17(70.39-168.44) | 6.60(4.22-10.09) | 133.93(85.02-206.66) | 6.67(4.23-10.29) | 21.57(17.93-24.18) | 0.03(0.02-0.03) |
| Peru | 535.86(342.82-815.41) | 6.46(4.13-9.82) | 618.18(394.40-944.46) | 6.48(4.14-9.90) | 15.36(14.72-15.83) | 0.02(0.01-0.02) |
| Philippines | 2640.89(1727.72-3932.90) | 10.47(6.85-15.60) | 3581.77(2297.00-5404.52) | 10.54(6.76-15.90) | 35.63(27.14-44.30) | 0.01(0.01-0.02) |
| Poland | 931.65(604.35-1392.67) | 9.73(6.31-14.54) | 573.63(373.71-852.74) | 9.75(6.35-14.49) | -38.43(-38.77--37.99) | -0.10(-0.16--0.03) |
| Portugal | 168.00(113.81-246.02) | 7.94(5.38-11.63) | 109.92(75.66-159.42) | 8.07(5.55-11.70) | -34.57(-36.45--31.77) | 0.02(-0.00-0.04) |
| Puerto Rico | 66.49(42.19-102.51) | 6.68(4.24-10.29) | 30.16(18.73-47.53) | 6.79(4.22-10.69) | -54.64(-57.08--53.05) | 0.05(0.04-0.06) |
| Qatar | 6.49(4.09-10.00) | 5.19(3.27-8.00) | 25.72(16.11-39.96) | 5.21(3.26-8.09) | 296.38(288.30-301.98) | -0.04(-0.06--0.02) |
| Republic of Korea | 1224.21(818.52-1765.21) | 10.77(7.20-15.52) | 649.31(427.43-946.76) | 10.69(7.04-15.59) | -46.96(-48.28--46.00) | -0.03(-0.04--0.01) |
| Republic of Moldova | 95.85(62.90-144.77) | 7.76(5.09-11.71) | 40.87(26.32-62.32) | 7.83(5.04-11.93) | -57.36(-58.77--56.35) | -0.01(-0.03-0.02) |
| Romania | 363.63(236.32-534.83) | 6.53(4.24-9.61) | 192.26(124.30-284.89) | 6.39(4.13-9.46) | -47.13(-47.81--46.60) | -0.13(-0.19--0.07) |
| Russian Federation | 3090.03(1981.68-4744.73) | 8.91(5.71-13.67) | 2341.66(1486.83-3636.63) | 8.98(5.70-13.95) | -24.22(-25.97--23.03) | -0.07(-0.11--0.03) |
| Rwanda | 232.41(151.31-340.46) | 6.85(4.46-10.03) | 344.22(222.04-509.92) | 6.93(4.47-10.26) | 48.11(43.66-51.35) | 0.03(0.02-0.03) |
| Saint Kitts and Nevis | 0.94(0.60-1.45) | 6.64(4.22-10.25) | 0.66(0.42-1.02) | 6.68(4.22-10.32) | -29.87(-31.02--29.01) | 0.03(0.02-0.04) |
| Saint Lucia | 3.42(2.18-5.26) | 6.63(4.23-10.21) | 1.99(1.26-3.09) | 6.72(4.24-10.39) | -41.68(-43.39--40.51) | 0.05(0.04-0.06) |
| Saint Vincent and the Grenadines | 2.74(1.73-4.24) | 6.67(4.21-10.32) | 1.67(1.05-2.60) | 6.70(4.21-10.42) | -38.93(-39.88--38.30) | 0.01(0.01-0.02) |
| Samoa | 6.62(4.33-9.82) | 9.29(6.07-13.78) | 7.35(4.80-10.90) | 9.19(6.01-13.64) | 10.97(10.23-11.57) | -0.03(-0.03--0.03) |
| San Marino | 0.34(0.23-0.49) | 8.19(5.51-11.93) | 0.36(0.24-0.53) | 8.18(5.49-11.97) | 7.19(6.21-8.06) | -0.04(-0.07--0.00) |
| Sao Tome and Principe | 3.92(2.54-5.80) | 6.92(4.49-10.23) | 5.42(3.44-8.09) | 6.97(4.41-10.39) | 38.19(34.25-40.95) | 0.00(-0.01-0.01) |
| Saudi Arabia | 346.93(217.88-537.58) | 5.29(3.32-8.20) | 402.94(249.85-628.04) | 5.33(3.30-8.30) | 16.15(11.37-19.32) | 0.02(0.01-0.03) |
| Senegal | 250.63(163.54-366.16) | 6.86(4.48-10.03) | 441.97(285.93-653.89) | 6.95(4.50-10.28) | 76.34(70.73-80.47) | 0.03(0.03-0.04) |
| Serbia | 161.63(107.38-236.80) | 7.45(4.95-10.92) | 100.51(66.49-148.09) | 7.57(5.01-11.15) | -37.81(-39.73--36.21) | -0.10(-0.21-0.01) |
| Seychelles | 2.22(1.44-3.31) | 9.37(6.08-13.94) | 2.19(1.42-3.27) | 9.37(6.08-13.96) | -1.31(-1.52--1.16) | -0.01(-0.02--0.00) |
| Sierra Leone | 126.09(83.45-182.21) | 6.96(4.60-10.05) | 246.42(160.05-362.78) | 6.89(4.48-10.14) | 95.43(87.11-101.97) | -0.04(-0.06--0.03) |
| Singapore | 70.68(47.98-101.00) | 10.88(7.39-15.55) | 87.30(59.79-124.99) | 10.75(7.36-15.39) | 23.50(20.37-26.66) | -0.01(-0.04-0.01) |
| Slovakia | 112.51(74.72-166.03) | 8.49(5.64-12.52) | 72.73(48.76-106.44) | 8.49(5.69-12.43) | -35.35(-36.06--34.40) | -0.14(-0.20--0.07) |
| Slovenia | 29.32(19.20-43.43) | 7.09(4.64-10.50) | 22.10(14.49-32.53) | 7.08(4.64-10.42) | -24.62(-25.29--23.70) | -0.10(-0.19-0.00) |
| Solomon Islands | 14.38(9.51-21.18) | 9.23(6.11-13.60) | 23.95(15.66-35.52) | 9.21(6.02-13.66) | 66.54(64.23-68.24) | -0.01(-0.02--0.01) |
| Somalia | 271.17(176.75-396.22) | 6.96(4.54-10.17) | 712.84(464.33-1043.08) | 6.90(4.50-10.10) | 162.88(161.66-163.77) | -0.03(-0.04--0.02) |
| South Africa | 1061.54(678.05-1618.47) | 7.80(4.98-11.89) | 1197.62(756.06-1834.27) | 7.88(4.97-12.06) | 12.82(10.60-14.30) | 0.01(-0.01-0.02) |
| South Sudan | 181.99(118.40-266.60) | 6.94(4.51-10.16) | 298.93(194.08-440.24) | 6.96(4.52-10.25) | 64.25(61.61-66.19) | 0.01(0.01-0.02) |
| Spain | 583.26(393.55-851.77) | 7.44(5.02-10.87) | 457.70(316.58-650.48) | 7.06(4.88-10.04) | -21.53(-29.28--13.50) | -0.22(-0.29--0.15) |
| Sri Lanka | 521.06(337.08-777.42) | 9.42(6.09-14.05) | 479.92(310.18-718.77) | 9.40(6.08-14.08) | -7.90(-9.05--7.11) | -0.04(-0.05--0.02) |
| Sudan | 472.06(298.75-725.62) | 5.31(3.36-8.16) | 878.66(547.72-1367.14) | 5.30(3.30-8.24) | 86.13(77.78-91.95) | -0.01(-0.02-0.00) |
| Suriname | 8.63(5.49-13.31) | 6.62(4.21-10.22) | 9.58(6.06-14.82) | 6.69(4.23-10.34) | 11.07(9.05-12.52) | 0.03(0.02-0.03) |
| Sweden | 166.16(112.85-236.86) | 10.76(7.31-15.34) | 192.33(127.43-284.79) | 10.56(7.00-15.64) | 15.75(8.11-25.08) | -0.07(-0.15-0.02) |
| Switzerland | 78.03(53.92-113.51) | 6.75(4.67-9.82) | 89.53(61.39-130.13) | 6.72(4.61-9.77) | 14.73(13.77-15.56) | 0.03(0.01-0.06) |
| Syrian Arab Republic | 314.01(197.10-486.73) | 5.30(3.33-8.22) | 199.06(124.32-311.43) | 5.43(3.39-8.50) | -36.61(-41.21--33.04) | 0.08(0.05-0.12) |
| Taiwan (Province of China) | 452.06(299.83-677.38) | 8.21(5.44-12.30) | 273.14(188.00-399.02) | 9.27(6.38-13.54) | -39.58(-48.37--26.87) | 0.47(0.34-0.59) |
| Tajikistan | 182.65(119.11-273.56) | 7.87(5.13-11.78) | 284.56(183.81-431.01) | 7.94(5.13-12.02) | 55.80(52.77-58.17) | 0.02(-0.00-0.04) |
| Thailand | 1587.41(1026.37-2375.05) | 9.42(6.09-14.09) | 923.12(593.90-1387.42) | 9.45(6.08-14.21) | -41.85(-42.58--41.35) | 0.01(0.01-0.02) |
| Timor-Leste | 30.97(20.68-45.34) | 9.31(6.22-13.63) | 48.85(31.83-72.50) | 9.38(6.11-13.93) | 57.74(50.94-63.10) | 0.04(0.03-0.04) |
| Togo | 121.37(78.89-178.08) | 6.89(4.48-10.10) | 229.47(148.17-340.12) | 6.93(4.48-10.28) | 89.06(84.43-92.48) | 0.02(0.01-0.02) |
| Tokelau | 0.06(0.04-0.08) | 9.19(5.92-13.87) | 0.04(0.02-0.06) | 9.39(5.99-14.25) | -33.53(-36.50--31.34) | 0.03(0.02-0.05) |
| Tonga | 3.85(2.52-5.71) | 9.22(6.03-13.66) | 3.58(2.34-5.32) | 9.18(6.00-13.64) | -6.99(-7.34--6.76) | -0.01(-0.01--0.01) |
| Trinidad and Tobago | 27.00(17.12-41.77) | 6.65(4.21-10.28) | 18.25(11.48-28.31) | 6.70(4.22-10.39) | -32.40(-33.93--31.22) | -0.02(-0.04--0.00) |
| Tunisia | 164.09(102.38-255.46) | 5.28(3.30-8.23) | 146.89(91.11-229.27) | 5.31(3.29-8.29) | -10.48(-11.88--9.53) | -0.04(-0.06--0.01) |
| Turkmenistan | 118.38(76.75-178.22) | 7.89(5.11-11.87) | 121.40(78.11-185.08) | 7.97(5.13-12.14) | 2.55(0.37-4.20) | 0.03(0.01-0.04) |
| Tuvalu | 0.32(0.22-0.47) | 9.27(6.23-13.52) | 0.34(0.22-0.51) | 9.25(6.00-13.80) | 7.00(0.46-12.25) | -0.00(-0.01--0.00) |
| Türkiye | 1134.11(719.37-1743.03) | 5.54(3.51-8.51) | 1033.80(651.86-1611.78) | 5.58(3.52-8.70) | -8.85(-11.00--7.45) | -0.01(-0.03-0.00) |
| Uganda | 575.92(379.63-835.09) | 6.84(4.51-9.92) | 1372.89(890.73-2025.53) | 6.92(4.49-10.21) | 138.38(129.71-145.17) | 0.04(0.04-0.04) |
| Ukraine | 1014.79(650.36-1558.71) | 8.92(5.72-13.70) | 576.23(363.95-896.11) | 9.08(5.74-14.12) | -43.22(-45.83--41.54) | -0.03(-0.07-0.00) |
| United Arab Emirates | 30.60(19.21-47.55) | 5.19(3.26-8.07) | 70.62(43.73-110.75) | 5.27(3.27-8.27) | 130.76(120.05-138.43) | -0.01(-0.04-0.01) |
| United Kingdom | 1070.99(711.48-1531.56) | 9.81(6.52-14.02) | 1201.97(802.03-1720.75) | 10.20(6.81-14.60) | 12.23(10.02-14.48) | 0.03(-0.04-0.10) |
| United Republic of Tanzania | 829.40(540.63-1212.18) | 6.87(4.48-10.04) | 1684.88(1092.39-2490.30) | 6.90(4.48-10.20) | 103.15(98.47-106.70) | 0.01(0.01-0.02) |
| United States of America | 3888.48(2398.96-5941.05) | 6.95(4.29-10.63) | 3710.80(2568.23-5187.73) | 6.24(4.32-8.73) | -4.57(-14.40-8.51) | -0.75(-1.04--0.45) |
| United States Virgin Islands | 2.12(1.36-3.26) | 6.65(4.25-10.21) | 0.90(0.57-1.40) | 6.72(4.24-10.42) | -57.61(-59.03--56.65) | 0.04(0.04-0.04) |
| Uruguay | 51.49(33.74-75.66) | 6.29(4.12-9.24) | 41.49(26.84-61.91) | 6.29(4.07-9.39) | -19.42(-21.34--18.08) | 0.01(0.01-0.02) |
| Uzbekistan | 674.19(437.36-1014.15) | 7.88(5.11-11.85) | 801.09(518.44-1210.31) | 7.94(5.14-11.99) | 18.82(17.88-19.57) | 0.01(-0.01-0.04) |
| Vanuatu | 6.31(4.19-9.27) | 9.26(6.16-13.61) | 10.75(7.03-15.97) | 9.23(6.03-13.70) | 70.50(66.13-73.74) | -0.02(-0.02--0.01) |
| Venezuela (Bolivarian Republic of) | 469.79(300.35-720.23) | 6.62(4.23-10.15) | 441.16(280.57-679.83) | 6.66(4.24-10.26) | -6.09(-7.75--4.97) | 0.01(0.00-0.02) |
| Viet Nam | 2479.12(1612.79-3691.51) | 9.35(6.08-13.92) | 2329.50(1505.10-3481.12) | 9.41(6.08-14.06) | -6.04(-7.56--4.91) | -0.00(-0.02-0.01) |
| Yemen | 373.49(235.40-576.68) | 5.26(3.32-8.13) | 729.28(454.72-1135.03) | 5.29(3.30-8.23) | 95.26(87.62-100.53) | -0.02(-0.05--0.00) |
| Zambia | 257.56(168.15-375.66) | 6.86(4.48-10.01) | 571.52(368.89-847.11) | 6.91(4.46-10.24) | 121.90(115.07-127.06) | 0.02(0.02-0.03) |
| Zimbabwe | 331.96(215.36-490.11) | 6.89(4.47-10.18) | 435.29(280.67-645.27) | 6.92(4.46-10.25) | 31.13(29.82-32.06) | -0.01(-0.01--0.00) |

EAPCs, estimated annual percentage changes; UI, uncertainty interval. a EAPC is expressed as 95% CI.

Supplementary Table 2. Deaths of Myocarditis in Children Between 1990 and 2021 at the National Level

|  | **Rate per 100000(95%UI)** |  |  |  |  |  |
| --- | --- | --- | --- | --- | --- | --- |
|  | **1990** | | **2021** | | **1990-2021** | |
| **Location** | **Deaths cases** | **Deaths rate** | **Deaths cases** | **Deaths rate** | **cases change** | **EAPCs** |
| Afghanistan | 12.78(1.79-48.19) | 0.30(0.04-1.12) | 23.78(3.54-79.00) | 0.17(0.02-0.56) | 86.094(-16.951-337.082) | -1.91(-2.06--1.76) |
| Albania | 4.77(2.26-7.27) | 0.43(0.20-0.65) | 1.05(0.58-1.60) | 0.24(0.13-0.36) | -78.040(-87.474--58.655) | -1.97(-2.14--1.80) |
| Algeria | 21.18(3.83-73.68) | 0.20(0.04-0.69) | 8.34(1.48-27.12) | 0.06(0.01-0.20) | -60.649(-87.511--12.994) | -2.87(-3.15--2.58) |
| American Samoa | 0.01(0.00-0.01) | 0.04(0.02-0.07) | 0.00(0.00-0.00) | 0.02(0.01-0.03) | -63.066(-83.864--17.736) | -2.36(-2.62--2.10) |
| Andorra | 0.02(0.01-0.03) | 0.20(0.12-0.33) | 0.00(0.00-0.01) | 0.04(0.02-0.05) | -79.316(-89.158--59.460) | -5.15(-5.39--4.92) |
| Angola | 15.84(4.07-44.92) | 0.34(0.09-0.95) | 16.47(9.30-27.49) | 0.11(0.06-0.18) | 3.922(-51.841-271.437) | -3.63(-3.96--3.30) |
| Antigua and Barbuda | 0.04(0.03-0.05) | 0.23(0.18-0.28) | 0.02(0.02-0.03) | 0.14(0.12-0.17) | -42.706(-56.721--27.589) | -1.41(-1.53--1.28) |
| Argentina | 26.64(22.44-31.65) | 0.26(0.22-0.31) | 3.79(3.00-4.64) | 0.04(0.03-0.05) | -85.778(-89.238--81.067) | -5.99(-6.47--5.51) |
| Armenia | 0.55(0.34-0.82) | 0.05(0.03-0.08) | 0.06(0.04-0.07) | 0.01(0.01-0.01) | -89.958(-94.222--80.554) | -4.55(-5.90--3.19) |
| Australia | 11.84(10.64-12.98) | 0.31(0.28-0.34) | 3.29(2.65-4.06) | 0.07(0.06-0.09) | -72.239(-78.420--64.996) | -4.69(-5.32--4.05) |
| Austria | 0.22(0.19-0.25) | 0.02(0.01-0.02) | 0.29(0.23-0.36) | 0.02(0.02-0.03) | 30.429(3.357-64.248) | 2.22(-0.20-4.69) |
| Azerbaijan | 4.69(1.95-7.63) | 0.19(0.08-0.31) | 2.48(1.52-3.63) | 0.11(0.06-0.15) | -47.072(-71.574-3.787) | -1.91(-2.40--1.41) |
| Bahamas | 0.07(0.06-0.09) | 0.09(0.07-0.11) | 0.03(0.02-0.05) | 0.04(0.03-0.06) | -52.260(-67.691--32.103) | -2.25(-2.69--1.81) |
| Bahrain | 0.12(0.08-0.20) | 0.07(0.05-0.12) | 0.05(0.03-0.09) | 0.02(0.01-0.03) | -56.684(-80.165--21.659) | -4.34(-4.56--4.12) |
| Bangladesh | 91.56(31.41-224.18) | 0.19(0.06-0.46) | 45.63(29.10-68.14) | 0.10(0.06-0.15) | -50.167(-78.776-42.017) | -1.59(-1.79--1.40) |
| Barbados | 0.05(0.04-0.07) | 0.09(0.07-0.11) | 0.03(0.02-0.04) | 0.07(0.05-0.09) | -40.810(-59.691--14.636) | -0.13(-0.44-0.18) |
| Belarus | 1.76(1.42-2.20) | 0.07(0.06-0.09) | 0.19(0.14-0.26) | 0.01(0.01-0.02) | -89.329(-92.716--84.942) | -5.58(-6.54--4.62) |
| Belgium | 0.38(0.33-0.43) | 0.02(0.02-0.02) | 0.42(0.33-0.52) | 0.02(0.02-0.03) | 10.476(-13.564-41.021) | 0.53(-1.59-2.70) |
| Belize | 0.17(0.12-0.24) | 0.21(0.15-0.29) | 0.06(0.05-0.08) | 0.05(0.04-0.07) | -62.567(-77.306--37.531) | -4.09(-4.63--3.56) |
| Benin | 7.64(3.51-16.21) | 0.32(0.15-0.67) | 8.55(3.90-16.46) | 0.14(0.06-0.27) | 11.807(-47.201-153.853) | -2.71(-2.90--2.52) |
| Bermuda | 0.01(0.00-0.01) | 0.06(0.04-0.09) | 0.00(0.00-0.01) | 0.05(0.03-0.07) | -47.342(-72.203-5.423) | -0.92(-1.57--0.28) |
| Bhutan | 0.58(0.17-1.15) | 0.22(0.06-0.44) | 0.26(0.16-0.41) | 0.14(0.08-0.22) | -54.583(-79.121-36.589) | -1.64(-2.06--1.22) |
| Bolivia (Plurinational State of) | 3.51(1.32-8.93) | 0.13(0.05-0.33) | 1.43(0.82-2.30) | 0.04(0.02-0.07) | -59.335(-81.833-13.109) | -3.60(-3.66--3.55) |
| Bosnia and Herzegovina | 1.59(0.86-2.43) | 0.15(0.08-0.22) | 0.32(0.16-0.52) | 0.07(0.03-0.11) | -79.701(-87.867--67.199) | -2.82(-3.14--2.50) |
| Botswana | 0.82(0.50-1.31) | 0.14(0.09-0.22) | 0.69(0.39-1.12) | 0.10(0.06-0.16) | -15.609(-54.614-37.695) | -0.85(-0.98--0.72) |
| Brazil | 94.25(78.83-113.37) | 0.18(0.15-0.22) | 46.26(36.58-57.65) | 0.10(0.08-0.12) | -50.917(-65.448--33.401) | -1.72(-2.37--1.06) |
| Brunei Darussalam | 0.54(0.38-0.74) | 0.59(0.42-0.82) | 0.28(0.20-0.39) | 0.30(0.21-0.41) | -47.545(-66.141--20.786) | -2.01(-2.22--1.80) |
| Bulgaria | 2.92(2.55-3.35) | 0.17(0.15-0.19) | 1.10(0.85-1.39) | 0.11(0.09-0.14) | -62.418(-72.386--49.214) | -1.70(-2.59--0.80) |
| Burkina Faso | 17.36(7.23-41.13) | 0.37(0.15-0.87) | 20.26(9.27-42.52) | 0.20(0.09-0.41) | 16.694(-38.530-135.945) | -2.08(-2.31--1.84) |
| Burundi | 11.41(3.15-25.20) | 0.44(0.12-0.96) | 5.63(2.73-10.09) | 0.10(0.05-0.17) | -50.632(-76.793-75.278) | -4.13(-4.58--3.67) |
| Cabo Verde | 0.18(0.06-0.48) | 0.12(0.04-0.31) | 0.06(0.01-0.15) | 0.04(0.01-0.11) | -65.904(-88.776-4.388) | -3.02(-3.16--2.88) |
| Cambodia | 7.26(2.77-20.24) | 0.16(0.06-0.43) | 5.33(3.27-8.74) | 0.10(0.06-0.17) | -26.535(-69.987-91.327) | -1.23(-1.29--1.16) |
| Cameroon | 13.68(6.28-25.97) | 0.28(0.13-0.53) | 18.49(8.61-34.63) | 0.14(0.06-0.26) | 35.148(-42.074-229.144) | -2.10(-2.35--1.85) |
| Canada | 8.54(7.67-9.44) | 0.15(0.13-0.16) | 4.75(4.06-5.49) | 0.08(0.07-0.09) | -44.427(-53.920--34.146) | -1.66(-2.34--0.98) |
| Central African Republic | 4.94(1.80-10.91) | 0.40(0.15-0.89) | 5.00(2.24-9.27) | 0.22(0.10-0.41) | 1.321(-52.041-146.039) | -1.92(-2.06--1.79) |
| Chad | 9.97(3.86-24.95) | 0.34(0.13-0.85) | 16.92(5.88-46.23) | 0.19(0.07-0.51) | 69.720(-19.521-238.469) | -2.25(-2.45--2.06) |
| Chile | 5.32(4.69-6.02) | 0.13(0.12-0.15) | 0.83(0.60-1.11) | 0.02(0.02-0.03) | -84.291(-89.321--77.794) | -4.66(-5.27--4.05) |
| China | 3333.62(2292.59-4634.40) | 1.05(0.72-1.46) | 640.12(450.93-816.43) | 0.25(0.17-0.31) | -80.798(-88.564--70.961) | -4.33(-4.59--4.07) |
| Colombia | 11.07(9.30-13.20) | 0.09(0.08-0.11) | 12.71(9.23-17.27) | 0.12(0.09-0.16) | 14.810(-17.954-58.946) | 1.14(0.70-1.59) |
| Comoros | 0.71(0.21-1.49) | 0.34(0.10-0.70) | 0.19(0.11-0.32) | 0.08(0.05-0.13) | -72.767(-88.261--5.104) | -4.83(-4.99--4.68) |
| Congo | 2.49(1.11-4.48) | 0.24(0.11-0.43) | 1.42(0.81-2.31) | 0.07(0.04-0.12) | -42.867(-72.995-49.357) | -3.94(-4.29--3.59) |
| Cook Islands | 0.00(0.00-0.00) | 0.01(0.00-0.01) | 0.00(0.00-0.00) | 0.00(0.00-0.01) | -81.791(-95.284--38.263) | -5.56(-6.16--4.96) |
| Costa Rica | 1.30(1.11-1.49) | 0.12(0.10-0.13) | 0.72(0.59-0.88) | 0.07(0.06-0.09) | -44.478(-55.987--29.275) | -1.43(-1.83--1.03) |
| Croatia | 1.87(1.69-2.06) | 0.19(0.17-0.21) | 0.48(0.40-0.58) | 0.08(0.07-0.10) | -74.175(-78.584--68.601) | -2.86(-3.22--2.50) |
| Cuba | 2.85(2.27-3.37) | 0.11(0.09-0.13) | 1.11(0.90-1.36) | 0.06(0.05-0.08) | -61.109(-70.621--47.752) | -1.37(-1.73--1.01) |
| Cyprus | 0.16(0.08-0.28) | 0.08(0.04-0.14) | 0.03(0.02-0.05) | 0.01(0.01-0.02) | -81.821(-92.539--51.968) | -6.12(-6.66--5.58) |
| Czechia | 0.87(0.77-0.98) | 0.04(0.03-0.04) | 0.30(0.22-0.39) | 0.02(0.01-0.02) | -65.913(-74.286--53.089) | -2.83(-3.11--2.55) |
| Côte d'Ivoire | 18.24(8.39-39.99) | 0.32(0.15-0.70) | 17.78(7.90-31.38) | 0.15(0.07-0.27) | -2.530(-54.392-135.980) | -2.37(-2.65--2.10) |
| Democratic People's Republic of Korea | 37.35(24.39-54.92) | 0.63(0.41-0.92) | 12.79(6.81-21.75) | 0.27(0.14-0.46) | -65.769(-83.249--39.677) | -2.49(-2.74--2.24) |
| Democratic Republic of the Congo | 58.34(22.24-123.49) | 0.33(0.13-0.70) | 37.53(18.46-67.93) | 0.10(0.05-0.18) | -35.672(-68.300-67.270) | -3.45(-3.72--3.17) |
| Denmark | 0.42(0.31-0.57) | 0.05(0.03-0.06) | 0.27(0.22-0.32) | 0.03(0.02-0.03) | -36.839(-54.823--8.448) | -1.97(-3.21--0.71) |
| Djibouti | 0.45(0.12-0.94) | 0.26(0.07-0.54) | 0.29(0.16-0.49) | 0.07(0.04-0.12) | -35.496(-72.862-159.616) | -4.27(-4.69--3.85) |
| Dominica | 0.05(0.04-0.08) | 0.21(0.15-0.30) | 0.03(0.02-0.04) | 0.20(0.13-0.29) | -46.884(-67.850--11.818) | -0.04(-0.45-0.38) |
| Dominican Republic | 3.75(1.84-6.92) | 0.14(0.07-0.26) | 2.48(1.33-4.32) | 0.08(0.05-0.15) | -33.789(-75.723-50.263) | -1.58(-1.82--1.33) |
| Ecuador | 3.38(2.74-4.26) | 0.09(0.07-0.11) | 0.99(0.72-1.32) | 0.02(0.01-0.03) | -70.637(-80.483--59.355) | -5.57(-6.47--4.66) |
| Egypt | 6.90(0.65-30.26) | 0.03(0.00-0.14) | 1.91(0.25-6.75) | 0.01(0.00-0.02) | -72.272(-94.082-32.423) | -5.22(-5.49--4.96) |
| El Salvador | 1.73(1.05-2.66) | 0.08(0.05-0.12) | 0.55(0.29-0.99) | 0.03(0.02-0.05) | -68.092(-86.236--26.277) | -3.22(-3.72--2.71) |
| Equatorial Guinea | 0.60(0.22-1.25) | 0.31(0.11-0.63) | 0.36(0.16-0.71) | 0.06(0.03-0.12) | -39.640(-75.598-59.463) | -5.98(-6.33--5.62) |
| Eritrea | 5.17(1.53-11.23) | 0.32(0.10-0.71) | 2.67(1.40-4.44) | 0.11(0.06-0.18) | -48.422(-78.361-87.084) | -3.57(-3.73--3.42) |
| Estonia | 0.12(0.10-0.14) | 0.03(0.03-0.04) | 0.00(0.00-0.00) | 0.00(0.00-0.00) | -97.976(-98.599--96.974) | -10.62(-11.83--9.40) |
| Eswatini | 0.71(0.40-1.12) | 0.18(0.10-0.29) | 0.48(0.25-0.82) | 0.12(0.06-0.20) | -31.879(-68.487-35.979) | -1.20(-1.30--1.09) |
| Ethiopia | 69.99(20.03-162.53) | 0.29(0.08-0.67) | 27.86(15.93-44.35) | 0.06(0.04-0.10) | -60.197(-82.037-42.064) | -5.33(-5.60--5.07) |
| Fiji | 0.43(0.29-0.64) | 0.15(0.10-0.23) | 0.50(0.30-0.79) | 0.18(0.11-0.29) | 17.643(-34.203-106.812) | 1.29(0.96-1.63) |
| Finland | 1.19(0.95-1.47) | 0.12(0.10-0.15) | 0.51(0.42-0.61) | 0.06(0.05-0.07) | -57.292(-69.173--42.996) | -2.47(-3.78--1.14) |
| France | 0.84(0.73-0.96) | 0.01(0.01-0.01) | 1.65(1.37-1.97) | 0.01(0.01-0.02) | 96.786(57.053-145.396) | 2.93(0.82-5.09) |
| Gabon | 0.66(0.28-1.23) | 0.16(0.07-0.30) | 0.37(0.20-0.61) | 0.06(0.03-0.10) | -44.429(-73.075-31.110) | -2.91(-3.17--2.64) |
| Gambia | 1.14(0.51-2.40) | 0.25(0.11-0.52) | 1.18(0.38-2.36) | 0.12(0.04-0.24) | 3.450(-61.358-171.518) | -2.80(-3.14--2.47) |
| Georgia | 1.30(0.82-1.87) | 0.09(0.06-0.14) | 0.16(0.12-0.21) | 0.02(0.02-0.03) | -87.980(-92.900--76.685) | -4.88(-7.03--2.68) |
| Germany | 5.44(4.35-7.32) | 0.04(0.03-0.06) | 4.78(3.96-5.78) | 0.04(0.03-0.05) | -12.094(-42.637-26.071) | 0.50(-1.30-2.34) |
| Ghana | 23.38(12.90-39.66) | 0.35(0.19-0.59) | 19.79(9.54-37.49) | 0.15(0.07-0.29) | -15.361(-56.676-100.810) | -2.48(-2.68--2.29) |
| Greece | 0.16(0.14-0.18) | 0.01(0.01-0.01) | 0.27(0.21-0.35) | 0.02(0.01-0.02) | 70.212(27.434-130.025) | 3.54(1.20-5.94) |
| Greenland | 0.07(0.03-0.13) | 0.52(0.21-0.93) | 0.03(0.01-0.05) | 0.25(0.09-0.44) | -59.581(-82.398--30.869) | -2.14(-2.38--1.89) |
| Grenada | 0.07(0.06-0.10) | 0.22(0.17-0.31) | 0.03(0.02-0.03) | 0.12(0.10-0.15) | -64.448(-74.545--52.492) | -1.37(-1.60--1.14) |
| Guam | 0.11(0.08-0.15) | 0.27(0.20-0.36) | 0.06(0.04-0.09) | 0.17(0.11-0.26) | -44.842(-64.511--15.209) | -0.46(-0.89--0.04) |
| Guatemala | 6.83(4.52-9.26) | 0.17(0.11-0.23) | 9.60(7.09-13.49) | 0.19(0.14-0.27) | 40.519(-17.091-161.471) | 0.44(-0.07-0.95) |
| Guinea | 12.19(4.80-28.92) | 0.44(0.17-1.05) | 11.03(5.47-21.61) | 0.18(0.09-0.36) | -9.527(-56.909-133.128) | -2.73(-2.84--2.61) |
| Guinea-Bissau | 1.91(0.87-4.28) | 0.40(0.18-0.89) | 1.38(0.53-2.78) | 0.15(0.06-0.31) | -27.814(-70.744-104.296) | -2.93(-3.22--2.65) |
| Guyana | 2.33(1.87-2.86) | 0.79(0.63-0.97) | 1.21(0.87-1.58) | 0.57(0.41-0.74) | -48.223(-65.416--24.643) | 0.10(-0.25-0.46) |
| Haiti | 23.44(7.49-70.56) | 0.86(0.28-2.60) | 30.34(13.36-54.87) | 0.70(0.31-1.26) | 29.453(-32.480-176.192) | -0.28(-0.46--0.10) |
| Honduras | 2.18(1.29-3.68) | 0.10(0.06-0.17) | 1.49(0.60-3.40) | 0.05(0.02-0.10) | -31.457(-79.143-60.323) | -2.16(-2.28--2.04) |
| Hungary | 1.58(1.33-2.10) | 0.07(0.06-0.10) | 0.37(0.28-0.48) | 0.03(0.02-0.03) | -76.711(-84.666--67.675) | -3.27(-3.63--2.92) |
| Iceland | 0.02(0.02-0.03) | 0.04(0.03-0.05) | 0.02(0.02-0.03) | 0.03(0.03-0.04) | -7.359(-27.034-21.654) | -0.41(-2.03-1.22) |
| India | 643.67(278.44-1204.32) | 0.20(0.09-0.37) | 394.89(261.16-539.09) | 0.11(0.07-0.15) | -38.650(-66.834-17.780) | -1.63(-1.82--1.43) |
| Indonesia | 98.41(45.44-264.82) | 0.15(0.07-0.39) | 83.03(54.41-133.94) | 0.12(0.08-0.20) | -15.629(-61.657-100.563) | -0.52(-0.66--0.39) |
| Iran (Islamic Republic of) | 30.07(9.92-61.10) | 0.12(0.04-0.24) | 3.96(2.14-6.13) | 0.02(0.01-0.03) | -86.828(-95.947--62.061) | -2.99(-4.05--1.92) |
| Iraq | 83.65(39.39-133.61) | 1.02(0.48-1.62) | 50.53(27.80-79.40) | 0.38(0.21-0.59) | -39.596(-70.982-19.018) | -2.90(-3.17--2.62) |
| Ireland | 0.38(0.34-0.44) | 0.04(0.03-0.04) | 0.41(0.34-0.48) | 0.04(0.03-0.05) | 7.555(-12.374-35.015) | 0.92(-1.31-3.20) |
| Israel | 0.45(0.36-0.54) | 0.03(0.02-0.04) | 0.40(0.31-0.52) | 0.02(0.01-0.02) | -10.328(-36.239-29.965) | -1.11(-3.22-1.05) |
| Italy | 14.80(13.89-15.62) | 0.16(0.15-0.17) | 0.79(0.65-0.98) | 0.01(0.01-0.01) | -94.638(-95.682--93.359) | -9.58(-10.82--8.32) |
| Jamaica | 0.83(0.53-1.09) | 0.10(0.06-0.13) | 0.34(0.25-0.47) | 0.06(0.04-0.08) | -58.598(-73.791--27.956) | -1.04(-1.49--0.58) |
| Japan | 26.97(25.90-28.08) | 0.12(0.11-0.12) | 14.61(13.19-16.14) | 0.09(0.09-0.10) | -45.845(-51.957--39.679) | -1.64(-2.66--0.61) |
| Jordan | 1.60(0.97-2.44) | 0.10(0.06-0.15) | 1.23(0.81-1.70) | 0.03(0.02-0.05) | -23.032(-54.675-33.224) | -3.66(-3.94--3.37) |
| Kazakhstan | 1.17(0.79-1.61) | 0.02(0.02-0.03) | 5.62(4.00-7.71) | 0.10(0.07-0.14) | 381.887(182.938-739.870) | 6.50(4.79-8.25) |
| Kenya | 10.91(4.00-19.73) | 0.10(0.04-0.18) | 5.55(3.07-10.25) | 0.03(0.02-0.05) | -49.111(-75.531-86.390) | -3.32(-3.52--3.12) |
| Kiribati | 0.10(0.05-0.21) | 0.35(0.17-0.71) | 0.11(0.05-0.19) | 0.27(0.12-0.45) | 11.319(-42.088-122.680) | -0.73(-0.84--0.61) |
| Kuwait | 1.30(1.01-1.65) | 0.23(0.18-0.30) | 1.28(1.03-1.61) | 0.15(0.12-0.19) | -1.754(-32.065-47.210) | -1.56(-2.18--0.94) |
| Kyrgyzstan | 3.52(2.76-4.21) | 0.21(0.16-0.25) | 2.63(2.32-2.98) | 0.12(0.10-0.13) | -25.155(-39.161--2.985) | -1.20(-2.44-0.06) |
| Lao People's Democratic Republic | 4.86(1.43-19.74) | 0.26(0.08-1.07) | 4.42(2.37-8.30) | 0.19(0.10-0.36) | -9.142(-61.116-179.757) | -1.00(-1.09--0.92) |
| Latvia | 0.12(0.09-0.15) | 0.02(0.02-0.03) | 0.00(0.00-0.00) | 0.00(0.00-0.00) | -97.209(-98.239--95.316) | -9.16(-10.32--7.99) |
| Lebanon | 1.47(0.73-2.56) | 0.14(0.07-0.24) | 0.70(0.44-1.08) | 0.05(0.03-0.08) | -52.688(-79.558-26.774) | -2.76(-3.07--2.45) |
| Lesotho | 0.88(0.56-1.32) | 0.13(0.08-0.19) | 0.67(0.41-1.06) | 0.11(0.06-0.17) | -23.445(-58.805-24.003) | -0.28(-0.46--0.10) |
| Liberia | 5.71(2.06-15.56) | 0.51(0.18-1.38) | 3.16(1.25-6.18) | 0.14(0.06-0.28) | -44.694(-77.506-73.676) | -3.83(-4.08--3.58) |
| Libya | 8.59(1.90-26.50) | 0.47(0.10-1.46) | 2.80(0.47-9.87) | 0.19(0.03-0.66) | -67.387(-87.806--42.624) | -2.73(-2.91--2.56) |
| Lithuania | 0.15(0.13-0.17) | 0.02(0.02-0.02) | 0.01(0.00-0.01) | 0.00(0.00-0.00) | -95.513(-97.054--93.130) | -8.39(-9.58--7.17) |
| Luxembourg | 0.02(0.02-0.02) | 0.03(0.03-0.04) | 0.02(0.02-0.03) | 0.02(0.02-0.03) | 15.551(-14.365-59.091) | -1.27(-2.20--0.33) |
| Madagascar | 26.18(6.99-55.47) | 0.48(0.13-1.02) | 16.99(8.38-29.07) | 0.14(0.07-0.25) | -35.114(-70.621-122.327) | -3.62(-3.77--3.48) |
| Malawi | 14.58(3.14-32.33) | 0.32(0.07-0.71) | 5.05(2.66-8.46) | 0.06(0.03-0.10) | -65.367(-83.938-70.686) | -5.32(-5.56--5.08) |
| Malaysia | 10.85(6.96-15.98) | 0.17(0.11-0.24) | 4.90(3.44-6.65) | 0.06(0.05-0.09) | -54.791(-73.036--20.685) | -2.98(-3.38--2.58) |
| Maldives | 0.25(0.10-0.54) | 0.24(0.09-0.52) | 0.09(0.06-0.14) | 0.09(0.06-0.14) | -64.880(-86.537-25.206) | -2.83(-3.00--2.67) |
| Mali | 12.32(4.92-30.19) | 0.30(0.12-0.73) | 13.60(5.84-29.85) | 0.12(0.05-0.26) | 10.395(-47.312-159.144) | -3.18(-3.37--3.00) |
| Malta | 0.11(0.09-0.13) | 0.12(0.10-0.14) | 0.10(0.07-0.13) | 0.15(0.11-0.20) | -9.491(-35.260-24.876) | 0.51(-0.77-1.81) |
| Marshall Islands | 0.03(0.01-0.06) | 0.14(0.07-0.27) | 0.02(0.01-0.04) | 0.13(0.06-0.24) | -29.302(-61.748-39.479) | -0.50(-0.76--0.25) |
| Mauritania | 2.14(1.06-4.20) | 0.23(0.11-0.45) | 1.69(0.60-3.16) | 0.09(0.03-0.17) | -20.694(-63.538-71.310) | -3.39(-3.74--3.04) |
| Mauritius | 0.04(0.03-0.05) | 0.01(0.01-0.01) | 0.09(0.07-0.12) | 0.04(0.04-0.06) | 144.168(83.762-230.111) | 7.11(3.92-10.39) |
| Mexico | 12.79(11.35-14.48) | 0.04(0.03-0.04) | 19.45(14.71-25.58) | 0.06(0.05-0.08) | 52.045(9.474-110.513) | 3.19(2.41-3.97) |
| Micronesia (Federated States of) | 0.08(0.04-0.15) | 0.18(0.08-0.32) | 0.03(0.01-0.05) | 0.10(0.05-0.17) | -63.382(-80.509--29.709) | -2.02(-2.14--1.90) |
| Monaco | 0.00(0.00-0.01) | 0.10(0.05-0.16) | 0.00(0.00-0.00) | 0.03(0.02-0.05) | -53.821(-76.671--11.396) | -4.79(-5.50--4.08) |
| Mongolia | 3.00(1.87-4.51) | 0.33(0.21-0.50) | 0.76(0.48-1.16) | 0.07(0.04-0.11) | -74.768(-86.641--50.729) | -4.61(-5.26--3.95) |
| Montenegro | 0.37(0.24-0.55) | 0.23(0.15-0.34) | 0.06(0.03-0.10) | 0.05(0.03-0.09) | -84.649(-92.879--68.793) | -4.87(-5.32--4.41) |
| Morocco | 30.50(4.96-107.11) | 0.31(0.05-1.09) | 9.47(1.18-30.87) | 0.10(0.01-0.32) | -68.951(-90.924--14.859) | -3.42(-3.77--3.08) |
| Mozambique | 13.31(2.14-32.72) | 0.21(0.03-0.53) | 7.42(3.46-13.55) | 0.05(0.02-0.10) | -44.243(-76.941-179.592) | -4.47(-4.67--4.27) |
| Myanmar | 38.88(13.45-128.81) | 0.26(0.09-0.87) | 32.70(19.63-58.52) | 0.21(0.13-0.37) | -15.913(-63.557-117.349) | -0.75(-0.90--0.60) |
| Namibia | 0.79(0.48-1.17) | 0.13(0.08-0.19) | 0.72(0.37-1.24) | 0.09(0.05-0.15) | -8.728(-54.776-61.289) | -1.03(-1.27--0.79) |
| Nauru | 0.01(0.00-0.01) | 0.18(0.07-0.34) | 0.01(0.00-0.01) | 0.16(0.08-0.27) | -17.172(-57.610-57.772) | -0.53(-0.90--0.16) |
| Nepal | 23.42(7.96-52.96) | 0.28(0.09-0.63) | 11.82(6.97-18.27) | 0.13(0.08-0.20) | -49.500(-78.429-62.523) | -2.29(-2.52--2.05) |
| Netherlands | 0.75(0.64-0.86) | 0.03(0.02-0.03) | 0.68(0.57-0.82) | 0.03(0.02-0.03) | -8.612(-29.111-17.044) | -0.00(-2.15-2.19) |
| New Zealand | 3.18(2.88-3.53) | 0.40(0.36-0.44) | 1.40(1.20-1.58) | 0.14(0.12-0.16) | -56.045(-63.145--49.038) | -2.86(-3.46--2.25) |
| Nicaragua | 2.04(0.88-3.37) | 0.11(0.05-0.19) | 0.81(0.46-1.31) | 0.04(0.02-0.07) | -60.388(-83.984-9.615) | -2.79(-3.08--2.49) |
| Niger | 15.18(5.01-39.62) | 0.37(0.12-0.98) | 17.34(7.87-33.00) | 0.14(0.06-0.26) | 14.256(-51.709-235.432) | -3.83(-4.19--3.46) |
| Nigeria | 152.64(78.30-372.25) | 0.39(0.20-0.95) | 153.67(89.46-315.93) | 0.15(0.09-0.31) | 0.671(-35.026-73.803) | -3.29(-3.59--2.99) |
| Niue | 0.00(0.00-0.00) | 0.14(0.07-0.23) | 0.00(0.00-0.00) | 0.38(0.18-0.63) | 30.372(-25.088-136.554) | 0.39(-0.65-1.45) |
| North Macedonia | 1.06(0.53-1.97) | 0.20(0.10-0.37) | 0.10(0.05-0.19) | 0.03(0.02-0.06) | -90.855(-96.773--73.409) | -5.35(-5.67--5.02) |
| Northern Mariana Islands | 0.01(0.01-0.01) | 0.07(0.04-0.10) | 0.01(0.00-0.01) | 0.05(0.03-0.08) | -26.853(-54.797-23.312) | -0.29(-0.85-0.26) |
| Norway | 0.96(0.89-1.03) | 0.12(0.11-0.13) | 0.21(0.18-0.24) | 0.02(0.02-0.03) | -78.173(-81.271--74.685) | -4.44(-5.19--3.69) |
| Oman | 2.42(1.04-4.73) | 0.29(0.12-0.56) | 1.11(0.65-1.71) | 0.09(0.05-0.14) | -54.286(-81.052-25.155) | -2.31(-3.11--1.50) |
| Pakistan | 163.57(73.59-294.62) | 0.33(0.15-0.60) | 223.92(124.84-349.66) | 0.26(0.15-0.41) | 36.899(-15.225-140.529) | 0.48(-0.01-0.97) |
| Palau | 0.01(0.00-0.01) | 0.14(0.06-0.24) | 0.00(0.00-0.00) | 0.08(0.04-0.12) | -60.685(-78.347--14.970) | -1.87(-2.04--1.70) |
| Palestine | 1.53(0.76-2.76) | 0.16(0.08-0.28) | 1.36(0.65-2.11) | 0.07(0.03-0.11) | -11.190(-64.772-88.073) | -2.32(-2.46--2.18) |
| Panama | 0.48(0.39-0.60) | 0.06(0.05-0.07) | 0.67(0.52-0.86) | 0.06(0.05-0.07) | 38.861(-0.947-94.821) | -0.09(-0.51-0.32) |
| Papua New Guinea | 5.96(2.57-11.93) | 0.35(0.15-0.70) | 12.79(5.48-23.29) | 0.33(0.14-0.59) | 114.472(12.783-310.151) | -0.09(-0.16--0.01) |
| Paraguay | 1.80(1.11-2.48) | 0.11(0.07-0.15) | 0.90(0.55-1.52) | 0.04(0.03-0.08) | -49.719(-73.618--10.078) | -2.95(-3.18--2.73) |
| Peru | 8.53(3.31-17.27) | 0.10(0.04-0.21) | 1.63(0.91-2.70) | 0.02(0.01-0.03) | -80.934(-93.226--40.106) | -5.79(-6.04--5.55) |
| Philippines | 31.24(14.79-52.42) | 0.12(0.06-0.21) | 25.31(18.42-34.74) | 0.07(0.05-0.10) | -18.976(-52.012-64.339) | -1.25(-1.40--1.09) |
| Poland | 28.25(26.23-30.48) | 0.29(0.27-0.32) | 2.72(2.24-3.33) | 0.05(0.04-0.06) | -90.365(-92.247--88.171) | -5.32(-5.60--5.05) |
| Portugal | 0.41(0.35-0.47) | 0.02(0.02-0.02) | 0.20(0.16-0.25) | 0.01(0.01-0.02) | -49.932(-60.879--35.336) | -0.79(-2.71-1.17) |
| Puerto Rico | 0.17(0.14-0.21) | 0.02(0.01-0.02) | 0.08(0.06-0.10) | 0.02(0.01-0.02) | -54.011(-64.787--39.429) | -0.13(-1.79-1.56) |
| Qatar | 0.16(0.09-0.27) | 0.13(0.07-0.22) | 0.16(0.10-0.29) | 0.03(0.02-0.06) | 4.373(-47.599-92.797) | -3.86(-4.06--3.66) |
| Republic of Korea | 32.78(21.56-46.97) | 0.29(0.19-0.41) | 5.20(3.65-7.03) | 0.09(0.06-0.12) | -84.122(-90.876--73.132) | -3.70(-3.91--3.48) |
| Republic of Moldova | 0.86(0.68-1.07) | 0.07(0.05-0.09) | 0.06(0.05-0.09) | 0.01(0.01-0.02) | -92.613(-95.288--87.863) | -5.56(-6.47--4.64) |
| Romania | 39.23(30.48-46.42) | 0.70(0.55-0.83) | 6.71(5.77-7.86) | 0.22(0.19-0.26) | -82.888(-86.657--76.417) | -3.52(-3.85--3.18) |
| Russian Federation | 34.16(32.32-36.25) | 0.10(0.09-0.10) | 9.84(8.26-11.44) | 0.04(0.03-0.04) | -71.201(-75.075--66.969) | -3.03(-4.11--1.93) |
| Rwanda | 13.69(3.92-31.70) | 0.40(0.12-0.93) | 3.58(1.95-5.79) | 0.07(0.04-0.12) | -73.830(-88.789--0.561) | -6.24(-6.68--5.80) |
| Saint Kitts and Nevis | 0.01(0.00-0.01) | 0.04(0.03-0.06) | 0.01(0.00-0.01) | 0.05(0.04-0.07) | -17.832(-48.598-21.781) | 0.74(0.39-1.09) |
| Saint Lucia | 0.14(0.11-0.17) | 0.27(0.22-0.33) | 0.05(0.04-0.06) | 0.16(0.12-0.21) | -66.251(-76.517--53.270) | -1.56(-1.74--1.38) |
| Saint Vincent and the Grenadines | 0.07(0.05-0.09) | 0.17(0.13-0.21) | 0.04(0.03-0.05) | 0.14(0.11-0.19) | -47.926(-62.894--27.037) | -0.86(-1.30--0.41) |
| Samoa | 0.11(0.06-0.19) | 0.16(0.08-0.27) | 0.08(0.04-0.13) | 0.10(0.05-0.17) | -30.184(-65.019-36.723) | -1.47(-1.60--1.34) |
| San Marino | 0.00(0.00-0.01) | 0.09(0.05-0.14) | 0.00(0.00-0.00) | 0.03(0.02-0.05) | -61.523(-83.884--17.704) | -2.77(-3.01--2.53) |
| Sao Tome and Principe | 0.16(0.07-0.35) | 0.29(0.13-0.61) | 0.07(0.01-0.20) | 0.09(0.02-0.25) | -57.932(-89.077-37.842) | -3.65(-4.07--3.23) |
| Saudi Arabia | 26.70(12.92-47.34) | 0.41(0.20-0.72) | 4.05(1.82-9.19) | 0.05(0.02-0.12) | -84.825(-95.336--43.458) | -7.06(-7.45--6.67) |
| Senegal | 12.92(6.88-28.16) | 0.35(0.19-0.77) | 9.19(3.20-18.01) | 0.14(0.05-0.28) | -28.876(-71.078-70.843) | -2.72(-3.01--2.43) |
| Serbia | 2.91(1.56-4.86) | 0.13(0.07-0.22) | 0.28(0.12-0.65) | 0.02(0.01-0.05) | -90.254(-97.134--68.974) | -6.58(-7.17--5.99) |
| Seychelles | 0.04(0.03-0.06) | 0.18(0.13-0.25) | 0.02(0.02-0.03) | 0.10(0.07-0.13) | -47.603(-65.227--24.312) | -1.06(-1.46--0.66) |
| Sierra Leone | 10.55(3.77-27.85) | 0.58(0.21-1.54) | 8.34(3.32-18.49) | 0.23(0.09-0.52) | -20.955(-63.668-88.130) | -3.26(-3.48--3.03) |
| Singapore | 7.63(6.87-8.45) | 1.18(1.06-1.30) | 1.46(1.26-1.72) | 0.18(0.15-0.21) | -80.812(-84.209--76.383) | -5.31(-5.68--4.94) |
| Slovakia | 0.92(0.68-1.24) | 0.07(0.05-0.09) | 0.29(0.20-0.42) | 0.03(0.02-0.05) | -68.215(-79.889--50.014) | -1.64(-1.93--1.36) |
| Slovenia | 0.04(0.04-0.05) | 0.01(0.01-0.01) | 0.01(0.01-0.02) | 0.00(0.00-0.01) | -74.326(-81.847--63.812) | -3.84(-4.38--3.29) |
| Solomon Islands | 0.23(0.10-0.42) | 0.15(0.07-0.27) | 0.26(0.12-0.47) | 0.10(0.05-0.18) | 10.961(-46.048-133.457) | -1.39(-1.55--1.22) |
| Somalia | 16.77(4.70-38.40) | 0.43(0.12-0.99) | 16.11(7.26-30.54) | 0.16(0.07-0.30) | -3.980(-53.116-191.814) | -2.99(-3.24--2.74) |
| South Africa | 22.27(10.87-34.34) | 0.16(0.08-0.25) | 9.33(6.94-13.70) | 0.06(0.05-0.09) | -58.089(-75.247--10.440) | -3.08(-3.21--2.95) |
| South Sudan | 11.55(1.72-26.35) | 0.44(0.07-1.00) | 9.73(2.79-24.02) | 0.23(0.06-0.56) | -15.800(-55.898-103.573) | -2.16(-2.69--1.63) |
| Spain | 0.99(0.88-1.12) | 0.01(0.01-0.01) | 1.51(1.20-1.90) | 0.02(0.02-0.03) | 52.487(17.040-100.889) | 2.22(-0.11-4.60) |
| Sri Lanka | 55.60(31.74-71.37) | 1.00(0.57-1.29) | 8.25(5.48-12.01) | 0.16(0.11-0.24) | -85.170(-91.141--72.489) | -7.30(-8.13--6.46) |
| Sudan | 35.61(4.24-164.04) | 0.40(0.05-1.84) | 24.43(3.96-82.53) | 0.15(0.02-0.50) | -31.410(-69.312-130.537) | -2.89(-3.13--2.64) |
| Suriname | 0.21(0.10-0.33) | 0.16(0.08-0.25) | 0.15(0.09-0.23) | 0.10(0.06-0.16) | -29.590(-60.013-27.147) | -1.17(-1.35--1.00) |
| Sweden | 3.57(3.13-4.06) | 0.23(0.20-0.26) | 0.91(0.79-1.05) | 0.05(0.04-0.06) | -74.397(-79.397--68.580) | -4.33(-4.98--3.68) |
| Switzerland | 0.27(0.21-0.36) | 0.02(0.02-0.03) | 0.17(0.14-0.21) | 0.01(0.01-0.02) | -38.010(-57.207--14.318) | -1.74(-3.45--0.00) |
| Syrian Arab Republic | 18.93(3.95-55.13) | 0.32(0.07-0.93) | 3.15(0.41-13.44) | 0.09(0.01-0.37) | -83.367(-96.172--56.727) | -4.73(-5.28--4.19) |
| Taiwan (Province of China) | 4.09(3.64-4.61) | 0.07(0.07-0.08) | 3.44(2.94-4.01) | 0.12(0.10-0.14) | -15.804(-30.347-0.209) | 4.35(3.35-5.37) |
| Tajikistan | 0.07(0.02-0.15) | 0.00(0.00-0.01) | 0.08(0.04-0.15) | 0.00(0.00-0.00) | 15.372(-57.791-305.693) | -0.80(-0.99--0.60) |
| Thailand | 16.56(9.89-28.43) | 0.10(0.06-0.17) | 7.27(4.74-9.64) | 0.07(0.05-0.10) | -56.090(-79.010--17.198) | -1.14(-1.52--0.77) |
| Timor-Leste | 0.81(0.28-2.98) | 0.24(0.08-0.90) | 0.99(0.55-1.82) | 0.19(0.11-0.35) | 22.535(-52.069-257.544) | -0.89(-1.09--0.69) |
| Togo | 5.14(2.53-9.83) | 0.29(0.14-0.56) | 3.72(1.63-6.56) | 0.11(0.05-0.20) | -27.600(-64.687-59.114) | -3.19(-3.40--2.98) |
| Tokelau | 0.00(0.00-0.00) | 0.12(0.06-0.22) | 0.00(0.00-0.00) | 0.44(0.18-0.80) | 131.824(7.091-435.081) | -0.24(-1.92-1.47) |
| Tonga | 0.04(0.02-0.07) | 0.11(0.06-0.17) | 0.03(0.01-0.05) | 0.07(0.04-0.13) | -35.087(-68.412-28.184) | -1.10(-1.23--0.98) |
| Trinidad and Tobago | 1.36(1.15-1.59) | 0.34(0.28-0.39) | 0.53(0.41-0.69) | 0.20(0.15-0.25) | -60.722(-70.731--46.438) | -1.18(-1.40--0.97) |
| Tunisia | 5.59(1.07-19.25) | 0.18(0.03-0.62) | 1.18(0.16-4.04) | 0.04(0.01-0.15) | -78.859(-95.322--45.156) | -4.04(-4.24--3.84) |
| Turkmenistan | 2.84(2.00-3.70) | 0.19(0.13-0.25) | 2.54(2.05-3.20) | 0.17(0.13-0.21) | -10.342(-39.474-39.385) | 0.04(-1.31-1.40) |
| Tuvalu | 0.01(0.00-0.02) | 0.25(0.11-0.46) | 0.00(0.00-0.01) | 0.10(0.06-0.17) | -56.147(-79.690-3.646) | -2.70(-2.98--2.43) |
| Türkiye | 17.23(6.14-38.17) | 0.08(0.03-0.19) | 4.46(2.10-7.18) | 0.02(0.01-0.04) | -74.129(-92.142--16.196) | -3.58(-3.73--3.42) |
| Uganda | 32.03(7.75-73.53) | 0.38(0.09-0.87) | 18.47(9.21-34.96) | 0.09(0.05-0.18) | -42.342(-73.168-100.079) | -4.61(-4.82--4.41) |
| Ukraine | 5.74(4.47-7.21) | 0.05(0.04-0.06) | 2.67(2.13-3.38) | 0.04(0.03-0.05) | -53.428(-67.523--30.908) | 0.60(-0.10-1.30) |
| United Arab Emirates | 1.52(0.74-2.86) | 0.26(0.13-0.48) | 0.78(0.50-1.37) | 0.06(0.04-0.10) | -48.370(-73.429-4.024) | -3.18(-3.67--2.68) |
| United Kingdom | 28.92(26.39-32.27) | 0.26(0.24-0.30) | 7.60(6.51-8.73) | 0.06(0.06-0.07) | -73.717(-79.249--68.094) | -4.29(-4.79--3.79) |
| United Republic of Tanzania | 34.97(8.83-74.67) | 0.29(0.07-0.62) | 20.67(11.23-34.01) | 0.08(0.05-0.14) | -40.893(-75.057-125.184) | -3.87(-4.05--3.68) |
| United States of America | 168.02(158.32-178.55) | 0.30(0.28-0.32) | 93.16(83.51-103.84) | 0.16(0.14-0.17) | -44.555(-51.632--36.475) | -2.11(-2.54--1.67) |
| United States Virgin Islands | 0.08(0.06-0.13) | 0.26(0.18-0.39) | 0.01(0.01-0.02) | 0.09(0.05-0.15) | -85.886(-92.695--73.555) | -2.66(-3.03--2.29) |
| Uruguay | 2.44(2.12-2.84) | 0.30(0.26-0.35) | 0.33(0.24-0.43) | 0.05(0.04-0.06) | -86.611(-90.545--82.138) | -6.28(-6.77--5.79) |
| Uzbekistan | 9.40(6.32-12.18) | 0.11(0.07-0.14) | 4.56(3.46-6.00) | 0.05(0.03-0.06) | -51.436(-67.318--18.476) | -1.56(-2.47--0.64) |
| Vanuatu | 0.13(0.07-0.23) | 0.20(0.11-0.33) | 0.16(0.08-0.28) | 0.14(0.07-0.24) | 19.754(-32.455-119.442) | -1.10(-1.23--0.97) |
| Venezuela (Bolivarian Republic of) | 7.65(6.55-9.50) | 0.11(0.09-0.13) | 4.86(3.50-6.60) | 0.07(0.05-0.10) | -36.436(-56.860--9.355) | -1.41(-1.89--0.92) |
| Viet Nam | 26.85(16.15-45.18) | 0.10(0.06-0.17) | 15.45(8.14-28.67) | 0.06(0.03-0.12) | -42.448(-72.866-12.768) | -0.91(-1.18--0.63) |
| Yemen | 22.17(3.02-98.07) | 0.31(0.04-1.38) | 21.23(3.38-70.91) | 0.15(0.02-0.51) | -4.262(-62.529-210.470) | -2.03(-2.28--1.77) |
| Zambia | 10.48(2.24-26.71) | 0.28(0.06-0.71) | 4.69(2.42-7.81) | 0.06(0.03-0.09) | -55.286(-81.267-121.975) | -5.14(-5.46--4.81) |
| Zimbabwe | 5.82(3.73-8.76) | 0.12(0.08-0.18) | 11.38(6.25-17.82) | 0.18(0.10-0.28) | 95.465(10.600-230.641) | 2.31(1.82-2.81) |

EAPCs, estimated annual percentage changes; UI, uncertainty interval. a EAPC is expressed as 95% CI.

Supplementary Table 3. DALYs of Myocarditis in Children Between 1990 and 2021 at the National Level

|  | **Rate per 100000(95%UI)** |  |  |  |  |  |
| --- | --- | --- | --- | --- | --- | --- |
|  | **1990** | | **2021** | | **1990-2021** | |
| **Location** | **DALYs cases** | **DALYs rate** | **DALYs cases** | **DALYs rate** | **cases change** | **EAPCs** |
| Afghanistan | 1136.53(163.77-4277.79) | 26.38(3.80-99.30) | 2115.03(334.47-6925.76) | 14.89(2.36-48.77) | 86.09(-14.79-341.84) | -1.92(-2.07--1.77) |
| Albania | 429.03(207.00-649.67) | 38.40(18.53-58.15) | 96.00(54.43-143.85) | 21.64(12.27-32.42) | -77.62(-87.11--58.51) | -1.93(-2.09--1.76) |
| Algeria | 1886.02(355.30-6509.74) | 17.59(3.31-60.70) | 750.40(152.86-2397.08) | 5.64(1.15-18.02) | -60.21(-86.57--13.36) | -2.83(-3.11--2.54) |
| American Samoa | 0.70(0.38-1.20) | 3.68(2.02-6.30) | 0.26(0.14-0.42) | 1.85(1.00-2.99) | -62.55(-82.99--19.06) | -2.31(-2.57--2.06) |
| Andorra | 1.75(1.06-2.85) | 18.39(11.16-29.98) | 0.43(0.30-0.60) | 4.24(2.97-5.86) | -75.30(-86.26--55.27) | -4.67(-4.88--4.46) |
| Angola | 1408.29(368.98-3972.68) | 29.87(7.83-84.26) | 1456.81(826.35-2419.79) | 9.56(5.42-15.87) | 3.45(-51.83-258.37) | -3.65(-3.98--3.31) |
| Antigua and Barbuda | 3.74(3.04-4.56) | 20.54(16.69-25.07) | 2.19(1.88-2.57) | 12.98(11.10-15.19) | -41.29(-55.25--26.61) | -1.34(-1.45--1.22) |
| Argentina | 2390.68(2016.80-2839.47) | 23.59(19.90-28.02) | 355.30(287.71-431.67) | 3.49(2.83-4.24) | -85.14(-88.66--80.49) | -5.88(-6.34--5.41) |
| Armenia | 51.50(32.95-74.58) | 4.94(3.16-7.15) | 5.86(4.56-7.53) | 0.99(0.77-1.27) | -88.61(-92.97--79.77) | -4.29(-5.53--3.03) |
| Australia | 1105.12(995.94-1221.61) | 29.19(26.31-32.27) | 376.33(302.13-458.34) | 7.92(6.36-9.65) | -65.95(-73.08--58.14) | -4.16(-4.79--3.52) |
| Austria | 21.51(18.76-24.53) | 1.60(1.39-1.82) | 32.98(27.27-40.31) | 2.54(2.10-3.11) | 53.32(27.52-89.71) | 2.69(0.34-5.10) |
| Azerbaijan | 415.50(178.84-676.50) | 17.12(7.37-27.87) | 221.05(138.68-321.54) | 9.36(5.87-13.62) | -46.80(-70.84-2.73) | -1.86(-2.31--1.40) |
| Bahamas | 6.50(5.16-8.19) | 8.06(6.40-10.16) | 3.23(2.38-4.31) | 3.98(2.94-5.31) | -50.37(-65.29--30.63) | -2.12(-2.55--1.70) |
| Bahrain | 10.67(6.91-17.70) | 6.53(4.24-10.84) | 4.91(3.10-8.35) | 1.66(1.04-2.81) | -53.95(-77.75--20.06) | -4.15(-4.38--3.92) |
| Bangladesh | 8235.40(2925.77-19891.65) | 16.84(5.98-40.67) | 4089.27(2674.15-6017.15) | 8.94(5.84-13.15) | -50.35(-78.87-36.36) | -1.61(-1.81--1.42) |
| Barbados | 4.89(3.96-6.02) | 7.85(6.35-9.66) | 3.04(2.24-4.10) | 6.46(4.76-8.70) | -37.86(-56.44--13.18) | -0.05(-0.34-0.24) |
| Belarus | 166.63(136.97-203.86) | 6.93(5.70-8.48) | 22.50(17.18-29.02) | 1.43(1.09-1.84) | -86.50(-90.08--81.73) | -4.93(-5.76--4.10) |
| Belgium | 35.99(31.42-41.20) | 1.99(1.74-2.28) | 48.71(39.72-60.59) | 2.55(2.08-3.17) | 35.36(11.32-67.49) | 1.23(-0.83-3.34) |
| Belize | 15.35(10.84-21.11) | 18.75(13.24-25.79) | 5.97(4.67-7.73) | 4.85(3.80-6.28) | -61.08(-76.01--35.99) | -4.00(-4.52--3.47) |
| Benin | 685.24(318.46-1442.43) | 28.29(13.15-59.56) | 770.49(357.40-1470.89) | 12.67(5.88-24.19) | 12.44(-46.44-152.30) | -2.68(-2.87--2.50) |
| Bermuda | 0.69(0.47-0.96) | 5.82(3.92-8.02) | 0.40(0.28-0.57) | 4.71(3.31-6.80) | -42.81(-67.86-5.67) | -0.66(-1.25--0.07) |
| Bhutan | 52.29(16.43-102.79) | 19.94(6.26-39.21) | 23.52(14.33-36.22) | 12.57(7.66-19.35) | -55.02(-78.98-29.76) | -1.67(-2.08--1.26) |
| Bolivia (Plurinational State of) | 312.35(119.86-788.97) | 11.63(4.46-29.37) | 129.03(75.89-204.16) | 3.70(2.18-5.86) | -58.69(-81.50-12.20) | -3.56(-3.61--3.51) |
| Bosnia and Herzegovina | 140.44(77.93-212.56) | 12.82(7.11-19.40) | 29.11(15.11-45.98) | 5.94(3.08-9.38) | -79.27(-87.35--66.74) | -2.76(-3.08--2.44) |
| Botswana | 72.23(45.19-115.34) | 12.23(7.65-19.53) | 61.32(34.94-98.38) | 8.78(5.00-14.09) | -15.10(-53.51-37.24) | -0.81(-0.94--0.68) |
| Brazil | 8476.08(7125.57-10171.78) | 16.32(13.72-19.58) | 4263.96(3373.85-5233.39) | 8.85(7.00-10.86) | -49.69(-64.26--32.54) | -1.66(-2.30--1.03) |
| Brunei Darussalam | 47.69(34.24-64.90) | 52.65(37.81-71.65) | 26.21(18.71-36.04) | 27.71(19.77-38.10) | -45.04(-63.70--18.90) | -1.89(-2.10--1.69) |
| Bulgaria | 262.78(229.96-300.57) | 15.14(13.24-17.31) | 104.08(81.60-129.78) | 10.66(8.36-13.30) | -60.39(-70.28--47.70) | -1.54(-2.40--0.68) |
| Burkina Faso | 1556.46(652.09-3668.16) | 32.98(13.82-77.73) | 1825.38(847.74-3794.47) | 17.60(8.17-36.58) | 17.28(-37.61-136.88) | -2.06(-2.30--1.83) |
| Burundi | 1016.25(287.31-2237.07) | 38.77(10.96-85.35) | 504.86(247.82-898.84) | 8.62(4.23-15.35) | -50.32(-76.04-72.15) | -4.11(-4.57--3.66) |
| Cabo Verde | 17.29(6.54-44.00) | 10.99(4.16-27.97) | 6.00(1.68-13.81) | 4.19(1.18-9.64) | -65.31(-87.44-1.11) | -2.98(-3.11--2.84) |
| Cambodia | 645.83(252.39-1799.68) | 13.86(5.42-38.61) | 473.81(296.14-774.90) | 9.26(5.79-15.15) | -26.64(-69.67-88.14) | -1.23(-1.30--1.16) |
| Cameroon | 1224.68(571.55-2307.02) | 25.09(11.71-47.26) | 1660.82(788.75-3079.76) | 12.33(5.86-22.87) | 35.61(-41.41-224.50) | -2.09(-2.34--1.84) |
| Canada | 823.48(743.61-916.11) | 14.32(12.93-15.93) | 584.04(483.24-708.69) | 9.46(7.83-11.48) | -29.08(-41.87--14.73) | -0.92(-1.57--0.27) |
| Central African Republic | 437.92(160.07-965.39) | 35.82(13.09-78.96) | 439.59(198.47-814.68) | 19.25(8.69-35.67) | 0.38(-52.42-142.45) | -1.95(-2.09--1.82) |
| Chad | 890.96(351.15-2221.89) | 30.45(12.00-75.93) | 1517.08(541.23-4099.17) | 16.83(6.00-45.47) | 70.28(-17.68-239.09) | -2.24(-2.44--2.05) |
| Chile | 484.42(426.86-546.69) | 12.20(10.75-13.76) | 84.40(63.14-108.54) | 2.31(1.73-2.97) | -82.58(-87.58--76.18) | -4.38(-4.95--3.80) |
| China | 294138.77(201842.13-408935.49) | 92.39(63.40-128.44) | 57042.39(40816.18-72392.72) | 21.97(15.72-27.88) | -80.61(-88.25--70.86) | -4.30(-4.55--4.05) |
| Colombia | 993.87(840.80-1180.40) | 8.52(7.21-10.12) | 1153.71(847.40-1557.64) | 10.87(7.98-14.68) | 16.08(-15.76-58.45) | 1.15(0.71-1.59) |
| Comoros | 63.72(18.76-132.66) | 29.96(8.82-62.37) | 17.45(9.92-28.85) | 7.27(4.13-12.01) | -72.61(-88.06--7.01) | -4.81(-4.96--4.66) |
| Congo | 221.25(99.11-397.22) | 21.01(9.41-37.72) | 125.75(72.01-202.80) | 6.52(3.73-10.51) | -43.16(-72.83-46.85) | -3.96(-4.31--3.61) |
| Cook Islands | 0.05(0.02-0.09) | 0.73(0.33-1.42) | 0.01(0.01-0.02) | 0.31(0.15-0.66) | -75.83(-91.34--39.54) | -4.04(-4.45--3.62) |
| Costa Rica | 119.43(102.97-137.59) | 10.62(9.16-12.24) | 68.26(57.53-82.41) | 6.71(5.66-8.10) | -42.84(-53.97--28.32) | -1.35(-1.74--0.96) |
| Croatia | 171.59(156.42-189.44) | 17.39(15.85-19.19) | 53.20(44.66-64.41) | 8.91(7.48-10.79) | -68.99(-73.99--62.95) | -2.38(-2.69--2.07) |
| Cuba | 258.36(208.09-303.36) | 10.32(8.31-12.11) | 106.55(86.77-130.86) | 6.00(4.88-7.36) | -58.76(-68.55--44.88) | -1.21(-1.55--0.87) |
| Cyprus | 15.25(7.92-25.67) | 7.71(4.00-12.97) | 3.57(2.41-5.52) | 1.63(1.10-2.52) | -76.61(-88.76--46.73) | -5.33(-5.77--4.90) |
| Czechia | 85.85(76.07-96.66) | 3.90(3.45-4.39) | 42.82(33.46-55.21) | 2.49(1.95-3.22) | -50.13(-59.99--35.81) | -1.62(-1.84--1.40) |
| Côte d'Ivoire | 1631.67(757.11-3565.11) | 28.61(13.27-62.51) | 1595.60(718.93-2794.54) | 13.79(6.21-24.15) | -2.21(-53.72-134.94) | -2.36(-2.63--2.09) |
| Democratic People's Republic of Korea | 3294.41(2153.91-4839.06) | 55.38(36.20-81.34) | 1115.28(610.29-1867.63) | 23.36(12.78-39.12) | -66.15(-83.10--40.78) | -2.53(-2.77--2.28) |
| Democratic Republic of the Congo | 5184.90(1995.09-10954.86) | 29.29(11.27-61.88) | 3315.87(1660.15-5938.56) | 8.73(4.37-15.63) | -36.05(-68.26-65.41) | -3.47(-3.74--3.20) |
| Denmark | 39.12(29.15-51.88) | 4.43(3.30-5.87) | 31.19(25.96-37.34) | 3.27(2.72-3.91) | -20.26(-41.85-10.78) | -1.17(-2.41-0.07) |
| Djibouti | 40.06(11.15-83.71) | 23.01(6.40-48.08) | 26.00(14.20-43.35) | 6.29(3.44-10.49) | -35.09(-72.45-152.69) | -4.25(-4.67--3.84) |
| Dominica | 4.67(3.35-6.64) | 18.82(13.48-26.77) | 2.45(1.57-3.57) | 17.92(11.50-26.13) | -47.52(-67.73--13.73) | -0.07(-0.48-0.33) |
| Dominican Republic | 336.46(166.74-616.65) | 12.48(6.19-22.88) | 223.77(123.99-385.56) | 7.62(4.22-13.12) | -33.49(-75.35-48.84) | -1.56(-1.80--1.31) |
| Ecuador | 300.89(245.48-375.87) | 7.78(6.35-9.72) | 92.06(70.27-119.60) | 1.82(1.39-2.36) | -69.40(-79.06--58.78) | -5.45(-6.32--4.57) |
| Egypt | 863.57(275.94-2940.37) | 3.89(1.24-13.26) | 392.69(177.63-811.06) | 1.07(0.48-2.20) | -54.53(-79.51-12.10) | -3.79(-3.95--3.64) |
| El Salvador | 154.31(94.14-235.95) | 7.15(4.36-10.93) | 50.02(27.84-87.73) | 2.75(1.53-4.82) | -67.59(-85.30--25.78) | -3.18(-3.68--2.69) |
| Equatorial Guinea | 53.56(20.00-109.89) | 27.20(10.15-55.81) | 32.42(14.44-61.49) | 5.54(2.47-10.51) | -39.47(-75.32-58.13) | -5.96(-6.30--5.61) |
| Eritrea | 461.07(140.33-992.46) | 28.96(8.82-62.35) | 237.88(126.08-393.67) | 9.42(4.99-15.59) | -48.41(-78.00-80.96) | -3.57(-3.72--3.41) |
| Estonia | 11.29(9.44-13.24) | 3.23(2.70-3.79) | 0.55(0.40-0.76) | 0.26(0.18-0.35) | -95.11(-96.51--93.35) | -8.26(-9.08--7.43) |
| Eswatini | 62.45(35.99-99.10) | 16.19(9.33-25.69) | 42.33(22.85-71.19) | 10.26(5.54-17.25) | -32.21(-68.05-34.72) | -1.20(-1.31--1.10) |
| Ethiopia | 6381.25(1988.72-14630.58) | 26.19(8.16-60.05) | 2562.76(1501.49-4007.98) | 5.78(3.39-9.04) | -59.84(-81.52-33.85) | -5.31(-5.58--5.05) |
| Fiji | 37.75(25.89-55.88) | 13.41(9.20-19.85) | 44.14(26.70-68.58) | 16.20(9.80-25.17) | 16.94(-34.42-102.65) | 1.28(0.94-1.61) |
| Finland | 110.02(90.35-135.34) | 11.40(9.36-14.03) | 59.19(49.64-71.94) | 6.99(5.86-8.49) | -46.20(-59.06--30.35) | -1.57(-2.83--0.29) |
| France | 93.33(80.64-107.52) | 0.80(0.69-0.92) | 228.05(187.34-279.85) | 1.96(1.61-2.41) | 144.35(99.94-192.20) | 3.58(1.59-5.61) |
| Gabon | 59.08(25.20-109.29) | 14.50(6.18-26.82) | 32.72(18.32-53.78) | 5.12(2.87-8.41) | -44.61(-72.63-28.38) | -2.92(-3.19--2.66) |
| Gambia | 101.82(46.64-213.35) | 22.07(10.11-46.25) | 105.44(35.96-208.46) | 10.61(3.62-20.98) | 3.56(-59.87-169.94) | -2.79(-3.12--2.46) |
| Georgia | 121.89(79.62-171.87) | 8.91(5.82-12.56) | 16.38(12.63-20.98) | 2.23(1.72-2.85) | -86.56(-91.63--75.96) | -4.57(-6.59--2.50) |
| Germany | 512.13(416.77-676.88) | 3.96(3.22-5.23) | 513.02(429.32-605.53) | 4.29(3.59-5.06) | 0.17(-33.11-35.99) | 1.04(-0.74-2.84) |
| Ghana | 2077.89(1159.76-3505.24) | 30.94(17.27-52.19) | 1758.66(848.34-3331.78) | 13.65(6.58-25.86) | -15.36(-56.45-98.14) | -2.48(-2.67--2.29) |
| Greece | 16.25(14.14-18.81) | 0.80(0.70-0.93) | 28.88(23.32-36.07) | 2.07(1.67-2.59) | 77.73(39.03-131.99) | 3.74(1.48-6.04) |
| Greenland | 6.67(2.75-11.83) | 46.90(19.34-83.19) | 2.82(1.08-4.70) | 23.99(9.19-40.01) | -57.73(-80.10--29.35) | -1.96(-2.20--1.73) |
| Grenada | 6.66(5.07-9.20) | 19.94(15.16-27.54) | 2.41(1.94-2.99) | 11.04(8.87-13.69) | -63.84(-74.18--51.98) | -1.35(-1.56--1.13) |
| Guam | 10.00(7.36-13.25) | 23.98(17.64-31.77) | 5.69(3.74-8.43) | 15.54(10.23-23.04) | -43.16(-63.06--13.04) | -0.46(-0.85--0.06) |
| Guatemala | 616.04(413.68-823.90) | 15.17(10.19-20.29) | 864.15(638.88-1206.52) | 17.51(12.95-24.45) | 40.27(-16.01-156.66) | 0.41(-0.08-0.91) |
| Guinea | 1089.89(432.29-2571.77) | 39.61(15.71-93.46) | 989.43(498.16-1929.35) | 16.37(8.24-31.91) | -9.22(-56.73-131.59) | -2.72(-2.83--2.60) |
| Guinea-Bissau | 169.98(77.88-379.03) | 35.24(16.14-78.57) | 123.11(48.59-247.24) | 13.71(5.41-27.53) | -27.58(-70.31-101.94) | -2.92(-3.21--2.64) |
| Guyana | 206.45(165.25-253.69) | 70.24(56.22-86.31) | 107.22(77.94-139.51) | 50.25(36.52-65.38) | -48.06(-64.88--24.76) | 0.06(-0.28-0.40) |
| Haiti | 2076.30(673.88-6245.39) | 76.53(24.84-230.19) | 2684.66(1197.49-4859.29) | 61.68(27.51-111.64) | 29.30(-32.50-175.16) | -0.28(-0.46--0.10) |
| Honduras | 193.47(117.07-324.40) | 8.76(5.30-14.68) | 135.23(58.23-300.01) | 4.13(1.78-9.15) | -30.10(-77.44-59.74) | -2.11(-2.22--2.00) |
| Hungary | 144.64(122.60-189.89) | 6.79(5.75-8.91) | 39.95(31.05-50.52) | 2.88(2.24-3.64) | -72.38(-81.02--62.31) | -2.81(-3.11--2.51) |
| Iceland | 2.27(1.92-2.65) | 3.58(3.03-4.18) | 2.42(2.02-2.93) | 3.59(2.99-4.34) | 6.78(-13.89-37.25) | 0.10(-1.47-1.69) |
| India | 58390.11(26263.64-107509.73) | 17.88(8.04-32.93) | 35946.17(24372.60-48406.88) | 9.81(6.65-13.21) | -38.44(-66.21-15.04) | -1.63(-1.81--1.44) |
| Indonesia | 8796.01(4263.70-23539.59) | 12.99(6.29-34.75) | 7390.05(4915.00-11757.10) | 10.98(7.30-17.47) | -15.98(-61.32-93.09) | -0.54(-0.67--0.41) |
| Iran (Islamic Republic of) | 2687.30(890.06-5440.35) | 10.59(3.51-21.43) | 365.61(211.29-549.03) | 1.81(1.05-2.72) | -86.40(-95.62--61.59) | -2.93(-3.97--1.88) |
| Iraq | 7404.76(3495.96-11814.38) | 89.90(42.44-143.44) | 4516.81(2484.94-7056.26) | 33.55(18.46-52.42) | -39.00(-70.11-20.29) | -2.87(-3.14--2.60) |
| Ireland | 35.40(31.40-40.14) | 3.60(3.20-4.09) | 51.80(42.78-63.74) | 5.19(4.29-6.39) | 46.33(19.51-80.88) | 1.79(-0.36-3.98) |
| Israel | 42.46(34.99-50.70) | 2.77(2.28-3.31) | 48.11(38.25-59.20) | 1.83(1.46-2.25) | 13.31(-16.94-56.58) | -0.55(-2.58-1.53) |
| Italy | 1360.16(1274.07-1447.27) | 14.74(13.80-15.68) | 108.43(86.93-134.94) | 1.43(1.14-1.78) | -92.03(-93.57--90.25) | -8.51(-9.60--7.40) |
| Jamaica | 74.67(49.25-97.64) | 8.94(5.90-11.69) | 32.35(24.34-43.02) | 5.54(4.17-7.37) | -56.68(-71.47--27.29) | -0.91(-1.35--0.47) |
| Japan | 2471.89(2355.76-2601.52) | 10.71(10.20-11.27) | 1614.14(1411.67-1858.93) | 10.45(9.14-12.04) | -34.70(-42.71--26.05) | -1.09(-2.06--0.11) |
| Jordan | 143.66(88.02-217.93) | 8.80(5.39-13.34) | 114.58(77.28-155.63) | 3.15(2.13-4.28) | -20.24(-52.58-36.13) | -3.54(-3.82--3.27) |
| Kazakhstan | 113.95(79.38-153.35) | 2.19(1.53-2.95) | 517.08(376.11-694.47) | 9.53(6.93-12.80) | 353.78(178.44-643.73) | 6.30(4.68-7.94) |
| Kenya | 988.22(380.30-1761.53) | 8.85(3.40-15.77) | 510.66(294.49-907.37) | 2.74(1.58-4.86) | -48.33(-74.66-79.36) | -3.30(-3.49--3.11) |
| Kiribati | 9.06(4.37-18.59) | 30.68(14.79-62.94) | 9.89(4.33-16.46) | 23.54(10.31-39.19) | 9.14(-43.24-118.35) | -0.78(-0.90--0.66) |
| Kuwait | 116.29(91.18-146.40) | 20.98(16.45-26.41) | 119.85(97.24-149.60) | 14.18(11.50-17.69) | 3.07(-28.14-52.32) | -1.42(-2.02--0.81) |
| Kyrgyzstan | 318.22(250.47-381.42) | 18.97(14.93-22.74) | 241.55(215.01-270.56) | 10.62(9.45-11.90) | -24.09(-38.26--3.12) | -1.14(-2.35-0.09) |
| Lao People's Democratic Republic | 429.51(129.34-1750.15) | 23.30(7.02-94.96) | 386.77(208.46-728.81) | 16.84(9.08-31.74) | -9.95(-61.57-173.77) | -1.04(-1.12--0.95) |
| Latvia | 11.49(8.99-14.06) | 2.02(1.58-2.47) | 0.72(0.52-1.01) | 0.24(0.18-0.34) | -93.70(-95.67--90.51) | -7.22(-8.02--6.42) |
| Lebanon | 134.10(67.93-230.38) | 12.82(6.49-22.03) | 67.25(44.92-102.75) | 5.26(3.51-8.04) | -49.85(-77.17-27.96) | -2.60(-2.89--2.30) |
| Lesotho | 77.99(49.92-116.07) | 11.43(7.31-17.00) | 59.09(36.01-91.98) | 9.37(5.71-14.59) | -24.24(-58.41-21.91) | -0.33(-0.51--0.15) |
| Liberia | 509.77(185.44-1384.94) | 45.11(16.41-122.54) | 283.48(116.08-552.05) | 12.97(5.31-25.25) | -44.39(-76.62-73.61) | -3.81(-4.05--3.56) |
| Libya | 768.84(173.50-2359.95) | 42.46(9.58-130.32) | 250.19(46.98-872.86) | 16.77(3.15-58.52) | -67.46(-86.80--42.35) | -2.74(-2.91--2.56) |
| Lithuania | 14.50(12.63-16.77) | 1.75(1.52-2.02) | 1.18(0.83-1.60) | 0.29(0.20-0.39) | -91.87(-94.10--89.06) | -6.49(-7.33--5.64) |
| Luxembourg | 1.94(1.69-2.24) | 2.94(2.56-3.39) | 3.32(2.63-4.29) | 3.28(2.60-4.24) | 70.91(33.57-127.61) | 0.12(-0.76-1.01) |
| Madagascar | 2332.41(648.37-4922.37) | 42.75(11.88-90.22) | 1508.52(741.27-2578.81) | 12.86(6.32-21.98) | -35.32(-70.46-114.99) | -3.64(-3.78--3.49) |
| Malawi | 1309.07(296.41-2889.49) | 28.77(6.52-63.51) | 454.61(244.52-757.02) | 5.60(3.01-9.32) | -65.27(-83.64-61.66) | -5.32(-5.56--5.09) |
| Malaysia | 977.06(638.26-1416.04) | 14.86(9.71-21.54) | 460.24(336.62-615.10) | 6.04(4.42-8.08) | -52.90(-70.82--19.74) | -2.85(-3.24--2.46) |
| Maldives | 22.32(8.98-47.59) | 21.25(8.55-45.31) | 8.00(5.34-12.16) | 7.99(5.33-12.14) | -64.14(-85.86-21.94) | -2.75(-2.92--2.58) |
| Mali | 1103.65(447.77-2685.60) | 26.72(10.84-65.03) | 1228.08(540.82-2667.88) | 10.61(4.67-23.05) | 11.27(-45.68-158.57) | -3.16(-3.34--2.98) |
| Malta | 9.68(8.11-11.44) | 11.06(9.26-13.07) | 9.68(7.45-12.43) | 15.12(11.63-19.42) | -0.05(-25.84-33.29) | 0.93(-0.35-2.22) |
| Marshall Islands | 2.80(1.36-5.24) | 12.77(6.18-23.87) | 1.95(0.92-3.65) | 11.17(5.28-20.93) | -30.41(-61.59-37.14) | -0.55(-0.80--0.30) |
| Mauritania | 191.37(95.76-373.84) | 20.70(10.36-40.44) | 153.16(56.31-281.61) | 8.27(3.04-15.20) | -19.97(-61.67-70.00) | -3.35(-3.69--3.00) |
| Mauritius | 3.75(3.23-4.36) | 1.14(0.98-1.32) | 8.46(6.87-10.40) | 4.08(3.31-5.02) | 125.41(72.36-199.27) | 6.29(3.50-9.15) |
| Mexico | 1173.07(1045.87-1335.67) | 3.51(3.13-4.00) | 1742.43(1341.80-2279.81) | 5.43(4.18-7.11) | 48.54(8.47-102.57) | 3.07(2.32-3.83) |
| Micronesia (Federated States of) | 7.15(3.46-12.97) | 15.56(7.52-28.24) | 2.58(1.32-4.59) | 8.42(4.31-15.01) | -63.92(-80.34--30.45) | -2.07(-2.20--1.95) |
| Monaco | 0.32(0.17-0.52) | 9.03(4.89-14.85) | 0.16(0.10-0.23) | 3.12(2.03-4.57) | -51.11(-74.22--10.43) | -4.44(-5.09--3.78) |
| Mongolia | 266.32(165.49-399.25) | 29.59(18.39-44.36) | 69.92(45.25-104.63) | 6.43(4.16-9.63) | -73.75(-85.61--50.00) | -4.49(-5.12--3.87) |
| Montenegro | 33.96(22.44-49.24) | 21.01(13.88-30.47) | 5.56(3.14-9.62) | 4.99(2.82-8.63) | -83.63(-91.49--68.97) | -4.68(-5.09--4.27) |
| Morocco | 2722.42(468.71-9455.07) | 27.82(4.79-96.62) | 853.87(125.43-2710.06) | 8.72(1.28-27.68) | -68.64(-89.58--14.45) | -3.40(-3.74--3.05) |
| Mozambique | 1195.56(206.19-2914.23) | 19.27(3.32-46.97) | 672.72(323.59-1216.56) | 4.72(2.27-8.53) | -43.73(-76.58-165.76) | -4.45(-4.65--4.25) |
| Myanmar | 3421.01(1196.94-11329.89) | 23.15(8.10-76.68) | 2862.20(1730.69-5092.22) | 18.33(11.08-32.61) | -16.33(-63.44-115.97) | -0.77(-0.92--0.62) |
| Namibia | 69.88(43.56-102.40) | 11.63(7.25-17.05) | 63.50(34.15-108.73) | 7.69(4.14-13.17) | -9.13(-54.21-57.95) | -1.03(-1.27--0.78) |
| Nauru | 0.68(0.28-1.23) | 16.23(6.74-29.08) | 0.56(0.29-0.93) | 14.11(7.28-23.44) | -18.03(-57.49-56.99) | -0.56(-0.93--0.20) |
| Nepal | 2118.99(757.85-4726.87) | 25.15(9.00-56.10) | 1070.32(653.38-1634.14) | 11.60(7.08-17.71) | -49.49(-78.21-53.53) | -2.30(-2.53--2.08) |
| Netherlands | 70.51(61.30-81.08) | 2.59(2.25-2.98) | 78.86(65.68-94.75) | 2.94(2.45-3.53) | 11.85(-10.64-37.59) | 0.73(-1.36-2.86) |
| New Zealand | 291.64(263.01-323.49) | 36.45(32.87-40.43) | 149.46(129.22-172.23) | 15.22(13.16-17.54) | -48.75(-56.39--40.23) | -2.45(-3.06--1.83) |
| Nicaragua | 183.12(79.91-301.81) | 10.05(4.39-16.57) | 74.64(44.08-118.37) | 3.77(2.23-5.98) | -59.24(-82.76-10.52) | -2.73(-3.02--2.44) |
| Niger | 1355.83(455.83-3514.95) | 33.37(11.22-86.51) | 1563.13(725.68-2922.69) | 12.25(5.69-22.90) | 15.29(-51.06-233.45) | -3.80(-4.16--3.44) |
| Nigeria | 13738.01(7180.14-33195.08) | 35.11(18.35-84.84) | 13990.56(8341.05-28405.69) | 13.77(8.21-27.96) | 1.84(-33.26-73.38) | -3.25(-3.54--2.96) |
| Niue | 0.10(0.05-0.16) | 12.24(6.48-19.90) | 0.12(0.06-0.21) | 32.10(16.00-53.48) | 25.88(-27.81-126.06) | 0.35(-0.66-1.38) |
| North Macedonia | 95.64(48.72-175.35) | 18.16(9.25-33.29) | 9.61(5.68-17.17) | 2.93(1.73-5.24) | -89.96(-96.16--72.16) | -5.12(-5.44--4.80) |
| Northern Mariana Islands | 0.73(0.47-1.06) | 5.98(3.85-8.75) | 0.53(0.34-0.76) | 4.71(2.99-6.79) | -27.02(-53.80-20.09) | -0.31(-0.82-0.21) |
| Norway | 90.58(83.64-97.49) | 11.35(10.48-12.21) | 27.02(22.83-32.63) | 2.92(2.47-3.53) | -70.17(-74.43--64.75) | -3.54(-4.22--2.86) |
| Oman | 217.56(96.29-421.78) | 25.89(11.46-50.19) | 102.02(63.34-154.25) | 8.34(5.18-12.61) | -53.10(-79.85-24.60) | -2.24(-3.05--1.43) |
| Pakistan | 14781.36(6787.99-26304.65) | 30.02(13.78-53.42) | 20024.11(11338.20-31201.59) | 23.44(13.27-36.52) | 35.47(-15.51-136.18) | 0.44(-0.05-0.93) |
| Palau | 0.57(0.26-0.99) | 12.49(5.79-21.67) | 0.23(0.12-0.35) | 6.90(3.76-10.73) | -60.45(-77.87--16.53) | -1.85(-2.02--1.68) |
| Palestine | 137.53(69.66-246.67) | 14.20(7.19-25.47) | 123.15(60.44-189.20) | 6.60(3.24-10.13) | -10.46(-63.86-86.47) | -2.30(-2.43--2.16) |
| Panama | 43.56(35.43-54.11) | 5.22(4.25-6.49) | 61.56(48.48-78.83) | 5.34(4.20-6.84) | 41.30(2.10-95.92) | -0.06(-0.46-0.35) |
| Papua New Guinea | 531.98(231.56-1059.73) | 31.29(13.62-62.33) | 1135.76(485.36-2079.50) | 29.00(12.39-53.09) | 113.50(11.67-307.47) | -0.10(-0.18--0.02) |
| Paraguay | 162.40(100.97-223.72) | 9.73(6.05-13.40) | 83.12(52.39-137.25) | 4.14(2.61-6.84) | -48.82(-72.46--10.20) | -2.90(-3.12--2.68) |
| Peru | 764.70(306.18-1534.88) | 9.21(3.69-18.49) | 155.47(93.64-250.69) | 1.63(0.98-2.63) | -79.67(-92.33--39.20) | -5.62(-5.87--5.37) |
| Philippines | 2775.75(1363.20-4616.71) | 11.01(5.41-18.31) | 2244.33(1643.45-3085.40) | 6.60(4.83-9.08) | -19.15(-51.75-59.47) | -1.28(-1.43--1.13) |
| Poland | 2600.97(2422.06-2817.52) | 27.16(25.29-29.42) | 302.50(252.33-368.11) | 5.14(4.29-6.25) | -88.37(-90.29--85.80) | -4.85(-5.06--4.63) |
| Portugal | 38.35(33.44-44.49) | 1.81(1.58-2.10) | 23.97(19.57-29.18) | 1.76(1.44-2.14) | -37.51(-49.36--21.59) | -0.03(-1.85-1.83) |
| Puerto Rico | 16.56(14.10-19.79) | 1.66(1.42-1.99) | 8.72(6.96-10.78) | 1.96(1.57-2.43) | -47.34(-58.35--33.70) | 0.26(-1.30-1.84) |
| Qatar | 14.25(8.12-24.47) | 11.40(6.49-19.57) | 16.19(10.41-27.02) | 3.28(2.11-5.47) | 13.63(-40.80-101.30) | -3.64(-3.81--3.46) |
| Republic of Korea | 2940.48(1980.77-4146.58) | 25.86(17.42-36.47) | 544.21(389.06-698.77) | 8.96(6.40-11.50) | -81.49(-88.62--69.96) | -3.21(-3.41--3.00) |
| Republic of Moldova | 80.46(64.09-98.63) | 6.51(5.19-7.98) | 6.86(5.18-9.06) | 1.31(0.99-1.73) | -91.48(-94.09--86.99) | -5.12(-5.93--4.30) |
| Romania | 3514.64(2739.52-4152.31) | 63.12(49.20-74.57) | 649.54(558.76-751.54) | 21.58(18.56-24.97) | -81.52(-85.53--74.62) | -3.28(-3.59--2.96) |
| Russian Federation | 3280.67(3059.91-3533.99) | 9.45(8.82-10.18) | 979.70(823.66-1144.48) | 3.76(3.16-4.39) | -70.14(-73.71--66.45) | -2.89(-3.88--1.88) |
| Rwanda | 1216.77(355.70-2800.39) | 35.86(10.48-82.54) | 322.33(178.02-516.39) | 6.49(3.58-10.39) | -73.51(-88.65--3.50) | -6.20(-6.64--5.76) |
| Saint Kitts and Nevis | 0.56(0.44-0.78) | 3.97(3.13-5.55) | 0.45(0.32-0.64) | 4.59(3.21-6.51) | -19.38(-49.06-17.18) | 0.69(0.34-1.05) |
| Saint Lucia | 12.68(10.27-15.40) | 24.60(19.92-29.88) | 4.32(3.33-5.65) | 14.56(11.22-19.03) | -65.91(-75.90--53.72) | -1.53(-1.70--1.36) |
| Saint Vincent and the Grenadines | 6.16(4.86-7.64) | 14.99(11.82-18.59) | 3.22(2.58-4.11) | 12.91(10.32-16.48) | -47.70(-62.22--27.79) | -0.85(-1.29--0.40) |
| Samoa | 10.05(5.37-16.67) | 14.10(7.53-23.40) | 7.02(3.76-11.86) | 8.78(4.70-14.84) | -30.13(-64.54-36.55) | -1.47(-1.61--1.33) |
| San Marino | 0.35(0.21-0.55) | 8.42(5.09-13.32) | 0.17(0.11-0.24) | 3.86(2.59-5.50) | -50.84(-74.94--8.74) | -2.10(-2.36--1.85) |
| Sao Tome and Principe | 14.71(6.55-30.59) | 25.97(11.56-53.98) | 6.26(1.33-17.48) | 8.04(1.71-22.46) | -57.47(-87.79-37.07) | -3.62(-4.03--3.21) |
| Saudi Arabia | 2368.74(1153.84-4187.06) | 36.15(17.61-63.89) | 366.23(174.49-808.48) | 4.84(2.31-10.69) | -84.54(-95.11--43.79) | -7.00(-7.38--6.62) |
| Senegal | 1148.33(612.33-2491.71) | 31.45(16.77-68.24) | 818.64(292.15-1589.89) | 12.87(4.59-25.00) | -28.71(-70.73-70.41) | -2.71(-3.00--2.42) |
| Serbia | 261.79(143.06-433.18) | 12.07(6.60-19.97) | 28.18(13.71-59.84) | 2.12(1.03-4.51) | -89.24(-96.37--68.14) | -6.24(-6.79--5.68) |
| Seychelles | 3.80(2.77-5.12) | 16.00(11.69-21.60) | 2.08(1.56-2.72) | 8.89(6.65-11.64) | -45.18(-62.43--21.83) | -0.98(-1.35--0.61) |
| Sierra Leone | 941.97(335.90-2476.25) | 51.97(18.53-136.61) | 746.20(299.81-1646.08) | 20.87(8.38-46.03) | -20.78(-63.42-88.57) | -3.25(-3.47--3.02) |
| Singapore | 672.89(605.35-749.07) | 103.62(93.22-115.35) | 165.62(138.06-201.63) | 20.39(17.00-24.83) | -75.39(-79.96--69.79) | -4.66(-5.00--4.31) |
| Slovakia | 85.55(64.66-113.50) | 6.45(4.88-8.56) | 29.56(21.46-41.24) | 3.45(2.51-4.81) | -65.44(-77.22--48.21) | -1.42(-1.68--1.16) |
| Slovenia | 4.53(3.93-5.19) | 1.10(0.95-1.25) | 1.84(1.42-2.40) | 0.59(0.45-0.77) | -59.45(-68.38--48.88) | -2.45(-2.82--2.07) |
| Solomon Islands | 20.76(9.38-37.16) | 13.33(6.03-23.87) | 22.85(10.72-41.15) | 8.79(4.12-15.82) | 10.08(-45.61-130.41) | -1.42(-1.58--1.25) |
| Somalia | 1497.36(429.67-3409.11) | 38.44(11.03-87.51) | 1444.57(656.61-2708.48) | 13.99(6.36-26.22) | -3.53(-52.30-185.38) | -2.98(-3.23--2.73) |
| South Africa | 1971.65(963.76-3019.55) | 14.48(7.08-22.18) | 826.92(614.69-1211.34) | 5.44(4.04-7.97) | -58.06(-75.12--11.83) | -3.03(-3.17--2.89) |
| South Sudan | 1034.28(169.90-2355.44) | 39.41(6.47-89.76) | 868.67(259.04-2130.57) | 20.23(6.03-49.61) | -16.01(-55.79-95.30) | -2.17(-2.69--1.64) |
| Spain | 95.80(84.25-107.74) | 1.22(1.08-1.37) | 169.47(139.03-207.87) | 2.61(2.15-3.21) | 76.90(42.59-123.88) | 2.63(0.38-4.93) |
| Sri Lanka | 4860.91(2838.52-6196.73) | 87.85(51.30-111.99) | 743.89(507.97-1062.53) | 14.57(9.95-20.82) | -84.70(-90.58--71.79) | -7.20(-8.02--6.37) |
| Sudan | 3167.37(396.69-14475.99) | 35.62(4.46-162.79) | 2166.54(373.55-7234.05) | 13.06(2.25-43.61) | -31.60(-68.43-126.36) | -2.90(-3.14--2.65) |
| Suriname | 18.92(9.38-29.38) | 14.52(7.20-22.55) | 13.37(7.85-20.14) | 9.33(5.48-14.06) | -29.31(-59.72-25.52) | -1.16(-1.33--0.99) |
| Sweden | 354.93(306.95-408.46) | 22.98(19.87-26.45) | 126.28(103.73-157.46) | 6.94(5.70-8.65) | -64.42(-71.35--55.64) | -3.52(-4.02--3.03) |
| Switzerland | 25.51(20.03-33.49) | 2.21(1.73-2.90) | 19.84(16.30-23.84) | 1.49(1.22-1.79) | -22.24(-44.80-3.14) | -0.98(-2.64-0.71) |
| Syrian Arab Republic | 1684.67(362.38-4875.20) | 28.45(6.12-82.32) | 273.92(41.96-1145.72) | 7.48(1.15-31.28) | -83.74(-95.73--58.44) | -4.79(-5.31--4.26) |
| Taiwan (Province of China) | 365.88(328.73-408.22) | 6.64(5.97-7.41) | 326.71(281.96-375.50) | 11.09(9.57-12.74) | -10.71(-25.11-5.53) | 4.48(3.51-5.45) |
| Tajikistan | 9.00(4.06-15.75) | 0.39(0.17-0.68) | 11.13(6.72-17.84) | 0.31(0.19-0.50) | 23.67(-39.76-164.03) | -0.64(-0.77--0.51) |
| Thailand | 1471.27(899.51-2482.06) | 8.73(5.34-14.72) | 657.14(441.95-856.46) | 6.73(4.53-8.77) | -55.34(-78.12--18.71) | -1.07(-1.42--0.71) |
| Timor-Leste | 72.07(25.04-264.74) | 21.67(7.53-79.59) | 87.30(48.97-161.18) | 16.77(9.41-30.96) | 21.12(-52.13-247.51) | -0.94(-1.14--0.74) |
| Togo | 458.95(227.39-874.89) | 26.04(12.90-49.64) | 334.92(148.96-586.68) | 10.12(4.50-17.73) | -27.02(-64.12-58.49) | -3.17(-3.37--2.96) |
| Tokelau | 0.06(0.03-0.11) | 10.78(5.16-19.15) | 0.14(0.06-0.26) | 36.87(15.88-66.60) | 122.35(3.57-399.86) | -0.30(-1.96-1.38) |
| Tonga | 3.94(2.16-6.19) | 9.43(5.18-14.81) | 2.55(1.33-4.53) | 6.53(3.42-11.62) | -35.38(-67.93-25.32) | -1.12(-1.24--1.00) |
| Trinidad and Tobago | 120.93(102.13-141.45) | 29.76(25.13-34.81) | 48.38(37.85-62.10) | 17.76(13.90-22.79) | -59.99(-69.86--46.04) | -1.12(-1.33--0.91) |
| Tunisia | 498.15(100.07-1700.51) | 16.04(3.22-54.76) | 107.71(19.23-353.92) | 3.89(0.70-12.80) | -78.38(-93.89--44.22) | -3.97(-4.18--3.76) |
| Turkmenistan | 254.86(181.59-329.15) | 16.98(12.10-21.93) | 226.84(184.55-282.22) | 14.88(12.11-18.52) | -11.00(-39.55-36.45) | 0.02(-1.30-1.35) |
| Tuvalu | 0.78(0.34-1.41) | 22.45(9.72-40.56) | 0.34(0.19-0.56) | 9.02(5.15-14.92) | -56.92(-79.85--0.26) | -2.77(-3.05--2.49) |
| Türkiye | 1550.06(561.50-3425.52) | 7.57(2.74-16.72) | 412.93(204.95-651.37) | 2.23(1.11-3.52) | -73.36(-91.26--17.51) | -3.50(-3.65--3.35) |
| Uganda | 2860.16(710.77-6525.73) | 33.97(8.44-77.51) | 1651.30(838.05-3105.72) | 8.32(4.22-15.66) | -42.27(-72.96-95.83) | -4.61(-4.81--4.40) |
| Ukraine | 550.36(438.82-672.32) | 4.84(3.86-5.91) | 261.51(211.41-322.60) | 4.12(3.33-5.08) | -52.48(-65.06--33.84) | 0.62(-0.05-1.30) |
| United Arab Emirates | 135.66(67.40-254.12) | 23.02(11.44-43.12) | 73.62(47.95-124.39) | 5.50(3.58-9.29) | -45.74(-71.36-5.64) | -3.06(-3.54--2.58) |
| United Kingdom | 2679.42(2437.92-2992.75) | 24.54(22.32-27.40) | 814.71(695.65-949.85) | 6.91(5.90-8.06) | -69.59(-75.65--63.55) | -3.77(-4.22--3.30) |
| United Republic of Tanzania | 3135.65(818.77-6651.63) | 25.97(6.78-55.08) | 1854.75(1017.01-3027.21) | 7.60(4.17-12.41) | -40.85(-74.42-119.72) | -3.87(-4.06--3.69) |
| United States of America | 15927.04(14864.28-17001.03) | 28.49(26.59-30.41) | 9693.55(8641.61-10888.83) | 16.31(14.54-18.32) | -39.14(-46.81--31.16) | -1.81(-2.22--1.39) |
| United States Virgin Islands | 7.53(5.17-11.05) | 23.56(16.19-34.59) | 1.19(0.75-1.95) | 8.87(5.60-14.57) | -84.22(-91.03--72.06) | -2.40(-2.73--2.06) |
| Uruguay | 220.40(192.25-255.04) | 26.92(23.49-31.16) | 31.38(23.85-40.21) | 4.76(3.62-6.10) | -85.76(-89.76--81.26) | -6.10(-6.57--5.63) |
| Uzbekistan | 854.78(579.94-1105.02) | 9.99(6.78-12.92) | 419.05(318.09-544.08) | 4.15(3.15-5.39) | -50.98(-66.46--18.63) | -1.56(-2.44--0.66) |
| Vanuatu | 11.93(6.40-19.67) | 17.51(9.39-28.89) | 14.16(7.52-24.35) | 12.15(6.45-20.89) | 18.75(-33.19-116.37) | -1.13(-1.26--1.01) |
| Venezuela (Bolivarian Republic of) | 687.99(592.35-848.30) | 9.70(8.35-11.96) | 439.23(322.82-591.79) | 6.63(4.87-8.93) | -36.16(-55.70--10.03) | -1.38(-1.85--0.91) |
| Viet Nam | 2463.24(1499.96-4055.07) | 9.29(5.66-15.29) | 1451.27(810.11-2639.18) | 5.86(3.27-10.66) | -41.08(-70.68-11.86) | -0.85(-1.12--0.58) |
| Yemen | 1979.38(281.23-8715.48) | 27.90(3.96-122.85) | 1887.84(318.94-6257.04) | 13.69(2.31-45.38) | -4.62(-61.75-205.98) | -2.04(-2.29--1.78) |
| Zambia | 941.73(214.59-2377.50) | 25.08(5.72-63.32) | 419.91(222.72-688.61) | 5.08(2.69-8.32) | -55.41(-80.93-108.34) | -5.16(-5.48--4.83) |
| Zimbabwe | 528.10(347.03-795.36) | 10.96(7.21-16.51) | 1003.06(574.07-1553.85) | 15.94(9.12-24.69) | 89.94(9.57-214.32) | 2.20(1.73-2.68) |

DALYs, disability-adjusted life-years; EAPCs, estimated annual percentage changes; UI, uncertainty interval. a EAPC is expressed as 95% CI.
